# Supplementary material for: Gray matter correlates of childhood maltreatment lack replicability in a multi-cohort brain-wide association study
Source: Nat Commun. 2025 Sep 16;16:8290. doi: 10.1038/s41467-025-62374-w (PMC12441139; doi:10.1038/s41467-025-62374-w)

Supplements

Table S1: *Demographic and clinical characteristics across all three cohorts*

Table S2: *Summary statistics for reported experiences of childhood maltreatment in the CTQ across cohorts*

Table S3: *Pairwise cohort comparison in sample characteristics including HC and MDD participants*

Table S4: *Pairwise cohort comparison of clinical characteristics of the MDD subgroups*

Table S5: *Differences in CTQ scales across HC and MDD groups in pooled sample and cohort-wise*

Table S6: *Results summary for pooled cohorts (n=3225) at a significance level of $p_{FWE}<.05$ –* ***sex-stratified for female subsample***

Table S7: *Results summary for pooled cohorts (n=3225) at a significance level of $p_{FWE}<.05$ –* ***sex-stratified for male subsample***

Table S8: *Results summary of single cohorts at a cohort-wise significance level of* $\boldsymbol{p_{unc}<.001}$

Table S9: *Results summary of single cohorts at a cohort-wise significance level of* $\boldsymbol{p_{unc}<.001}$ ***– sex-stratified for female subsample***

Table S10: *Results summary of single cohorts at a cohort-wise significance level of* $\boldsymbol{p_{unc}<.001}$ ***– sex-stratified for male subsample***

Table S11: *Results summary of single cohorts at a cohort-wise significance level of* $\boldsymbol{p_{unc}<.01}$

Table S12: *Results summary of single cohorts at a cohort-wise significance level of* $\boldsymbol{p_{unc}<.01}$ ***– sex-stratified for female subsample***

Table S13: *Results summary of single cohorts at a cohort-wise significance level of* $\boldsymbol{p_{unc}<.01}$ ***– sex-stratified for male subsample***

Table S14: *Replicability across cohorts indicated by spatial overlap in significance at a cohort-wise level of* $\boldsymbol{p_{unc}<.001}$ ***– sex-stratified for female subsample***

Table S15: *Replicability across cohorts indicated by spatial overlap in significance at a cohort-wise level of* $\boldsymbol{p_{unc}<.001}$ ***– sex-stratified for male subsample***

Table S16: *Replicability across cohorts indicated by spatial overlap in significance at a cohort-wise level of* $\boldsymbol{p_{unc}<.01}$

Table S17: *Replicability across cohorts indicated by spatial overlap in significance at a cohort-wise level of* $\boldsymbol{p_{unc}<.01}$ ***– sex-stratified for female subsample***

Table S18: *Replicability across cohorts indicated by spatial overlap in significance at a level of* $\boldsymbol{p_{unc}<.01}$ ***– sex-stratified for male subsample***

Table S19: *Results summary of pooled and single cohorts for parcellation-based Freesurfer analysis and global gray matter density analysis significant at $p_{FDR}<.05$*

Table S20: *Replicability across cohorts indicated by spatial overlap in significance at a level of $p_{FDR}<.05$ for parcellation-based Freesurfer and global gray matter density analysis*

Figure S1: *Age distribution across cohorts and diagnosis groups*

Figure S2: *Association between CTQ scales, demographic variables and clinical variables within the **MACS** cohort*

Figure S3: *Association between CTQ scales, demographic variables and clinical variables within the **MNC** cohort*

Figure S4: *Association between CTQ scales, demographic variables and clinical variables within the **BiDirect** cohort*

Figure S5: *Distributions of CTQ scales across HC and MDD groups stratified by cohorts*

Figure S6: *Significant clusters across cohort-wise analyses (replicability analysis) for **model 1** – CTQ sum in HC and MDD samples controlling for MDD diagnosis*

Figure S7: *Significant clusters across cohort-wise analyses (replicability analysis) for **model 2** – CTQ sum as a predictor in HC and MDD samples without controlling for MDD diagnosis*

Figure S8: *Significant clusters across cohort-wise analyses (replicability analysis) for **model 3** – CTQ sum as a predictor in HC subsamples*

Figure S9: *Significant clusters across cohort-wise analyses (replicability analysis) for **model 4** – CTQ sum as a predictor in MDD subsamples*

Figure S10: *Significant clusters across cohort-wise analyses (replicability analysis) for **model 5** – CTQ abuse subscales as a predictor in HC and MDD samples controlling for MDD diagnosis*

Figure S11: *Significant clusters across cohort-wise analyses (replicability analysis) for **model 6** – CTQ neglect subscales as a predictor in HC and MDD samples controlling for MDD diagnosis*

Figure S12: *Significant clusters across cohort-wise analyses (replicability analysis) for **model 7** – CTQ subscale emotional abuse as a predictor in HC and MDD samples controlling for MDD diagnosis*

Figure S13: *Significant clusters across cohort-wise analyses (replicability analysis) for **model 8** – CTQ subscale physical abuse as a predictor in HC and MDD samples controlling for MDD diagnosis*

Figure S14: *Significant clusters across cohort-wise analyses (replicability analysis) for **model 9** – CTQ subscale sexual abuse as a predictor in HC and MDD samples controlling for MDD diagnosis*

Figure S15: *Significant clusters across cohort-wise analyses (replicability analysis) for **model 10** – CTQ subscale emotional neglect as a predictor in HC and MDD samples controlling for MDD diagnosis*

Figure S16: *Significant clusters across cohort-wise analyses (replicability analysis) for **model 11** – CTQ subscale physical neglect as a predictor in HC and MDD samples controlling for MDD diagnosis*

Figure S17: *Significant clusters across cohort-wise analyses (replicability analysis) for **model 12** – CTQ extreme severity compared to none in HC and MDD samples controlling for MDD diagnosis*

Figure S18: *Significant clusters across cohort-wise analyses (replicability analysis) for **model 13** – CTQ extreme severity compared to none in HC and MDD samples without controlling for MDD diagnosis*

Figure S19: *Significant clusters across cohort-wise analyses (replicability analysis) for **model 14** – CTQ extreme severity compared to none in HC subsamples*

Figure S20: *Significant clusters across cohort-wise analyses (replicability analysis) for **model 15** – CTQ extreme severity compared to none in MDD subsamples*

Figure S21: *Significant clusters across cohort-wise analyses (replicability analysis) for* ***model 16*** *– CTQ sum as a predictor in medication-naïve MDD subsamples*

Figure S22: *Significant clusters across cohort-wise analyses (replicability analysis) for* ***model 17*** *– interaction term between CTQ sum and age as a predictor in HC subsamples*

Figure S23: *Significant clusters across cohort-wise analyses (replicability analysis) for* ***model 18*** *– interaction term between CTQ sum and age as a predictor in MDD subsamples*

Figure S24: *Significant clusters across cohort-wise analyses (replicability analysis) for* ***model 1*** *– CTQ sum in HC and MDD samples controlling for MDD diagnosis –* ***sex-stratified for female subsample***

Figure S25: *Significant clusters across cohort-wise analyses (replicability analysis) for* ***model 2*** *– CTQ sum as a predictor in HC and MDD samples without controlling for MDD diagnosis –* ***sex-stratified for female subsample***

Figure S26: *Significant clusters across cohort-wise analyses (replicability analysis) for* ***model 3*** *– CTQ sum as a predictor in HC subsamples –* ***sex-stratified for female subsample***

Figure S27: *Significant clusters across cohort-wise analyses (replicability analysis) for* ***model 4*** *– CTQ sum as a predictor in MDD subsamples –* ***sex-stratified for female subsample***

Figure S28: *Significant clusters across cohort-wise analyses (replicability analysis) for* ***model 5*** *– CTQ abuse subscales as a predictor in HC and MDD samples controlling for MDD diagnosis –* ***sex-stratified for female subsample***

Figure S29: *Significant clusters across cohort-wise analyses (replicability analysis) for* ***model 6*** *– CTQ neglect subscales as a predictor in HC and MDD samples controlling for MDD diagnosis –* ***sex-stratified for female subsample***

Figure S30: *Significant clusters across cohort-wise analyses (replicability analysis) for* ***model 7*** *– CTQ subscale emotional abuse as a predictor in HC and MDD samples controlling for MDD diagnosis –* ***sex-stratified for female subsample***

Figure S31: *Significant clusters across cohort-wise analyses (replicability analysis) for* ***model 8*** *– CTQ subscale physical abuse as a predictor in HC and MDD samples controlling for MDD diagnosis –* ***sex-stratified for female subsample***

Figure S32: *Significant clusters across cohort-wise analyses (replicability analysis) for* ***model 9*** *– CTQ subscale sexual abuse as a predictor in HC and MDD samples controlling for MDD diagnosis –* ***sex-stratified for female subsample***

Figure S33: *Significant clusters across cohort-wise analyses (replicability analysis) for* ***model 10*** *– CTQ subscale emotional neglect as a predictor in HC and MDD samples controlling for MDD diagnosis –* ***sex-stratified for female subsample***

Figure S34: *Significant clusters across cohort-wise analyses (replicability analysis) for* ***model 11*** *– CTQ subscale physical neglect as a predictor in HC and MDD samples controlling for MDD diagnosis –* ***sex-stratified for female subsample***

Figure S35: *Significant clusters across cohort-wise analyses (replicability analysis) for* ***model 12*** *– CTQ extreme severity compared to none in HC and MDD samples controlling for MDD diagnosis –* ***sex-stratified for female subsample***

Figure S36: *Significant clusters across cohort-wise analyses (replicability analysis) for* ***model 13*** *– CTQ extreme severity compared to none in HC and MDD samples without controlling for MDD diagnosis –* ***sex-stratified for female subsample***

Figure S37: *Significant clusters across cohort-wise analyses (replicability analysis) for* ***model 14*** *– CTQ extreme severity compared to none in HC subsamples –* ***sex-stratified for female subsample***

Figure S38: *Significant clusters across cohort-wise analyses (replicability analysis) for* ***model 15*** *– CTQ extreme severity compared to none in MDD subsamples –* ***sex-stratified for female subsample***

Figure S39: *Significant clusters across cohort-wise analyses (replicability analysis) for* ***model 16*** *– CTQ sum as a predictor in medication-naïve MDD subsamples –* ***sex-stratified for female subsample***

Figure S40: *Significant clusters across cohort-wise analyses (replicability analysis) for* ***model 17*** *– interaction term between CTQ sum and age as a predictor in HC subsamples –* ***sex-stratified for female subsample***

Figure S41: *Significant clusters across cohort-wise analyses (replicability analysis) for* ***model 18*** *– interaction term between CTQ sum and age as a predictor in MDD subsamples –* ***sex-stratified for female subsample***

Figure S42: *Significant clusters across cohort-wise analyses (replicability analysis) for* ***model 1*** *– CTQ sum in HC and MDD samples controlling for MDD diagnosis –* ***sex-stratified for male subsample***

Figure S43: *Significant clusters across cohort-wise analyses (replicability analysis) for* ***model 2*** *– CTQ sum as a predictor in HC and MDD samples without controlling for MDD diagnosis –* ***sex-stratified for male subsample***

Figure S44: *Significant clusters across cohort-wise analyses (replicability analysis) for* ***model 3*** *– CTQ sum as a predictor in HC subsamples –* ***sex-stratified for male subsample***

Figure S45: *Significant clusters across cohort-wise analyses (replicability analysis) for* ***model 4*** *– CTQ sum as a predictor in MDD subsamples –* ***sex-stratified for male subsample***

Figure S46: *Significant clusters across cohort-wise analyses (replicability analysis) for* ***model 5*** *– CTQ abuse subscales as a predictor in HC and MDD samples controlling for MDD diagnosis –* ***sex-stratified for male subsample***

Figure S47: *Significant clusters across cohort-wise analyses (replicability analysis) for* ***model 6*** *– CTQ neglect subscales as a predictor in HC and MDD samples controlling for MDD diagnosis –* ***sex-stratified for male subsample***

Figure S48: *Significant clusters across cohort-wise analyses (replicability analysis) for* ***model 7*** *– CTQ subscale emotional abuse as a predictor in HC and MDD samples controlling for MDD diagnosis –* ***sex-stratified for male subsample***

Figure S49: *Significant clusters across cohort-wise analyses (replicability analysis) for* ***model 8*** *– CTQ subscale physical abuse as a predictor in HC and MDD samples controlling for MDD diagnosis –* ***sex-stratified for male subsample***

Figure S50: *Significant clusters across cohort-wise analyses (replicability analysis) for* ***model 9*** *– CTQ subscale sexual abuse as a predictor in HC and MDD samples controlling for MDD diagnosis –* ***sex-stratified for male subsample***

Figure S51: *Significant clusters across cohort-wise analyses (replicability analysis) for* ***model 10*** *– CTQ subscale emotional neglect as a predictor in HC and MDD samples controlling for MDD diagnosis –* ***sex-stratified for male subsample***

Figure S52: *Significant clusters across cohort-wise analyses (replicability analysis) for* ***model 11*** *– CTQ subscale physical neglect as a predictor in HC and MDD samples controlling for MDD diagnosis –* ***sex-stratified for male subsample***

Figure S53: *Significant clusters across cohort-wise analyses (replicability analysis) for* ***model 12*** *– CTQ extreme severity compared to none in HC and MDD samples controlling for MDD diagnosis –* ***sex-stratified for male subsample***

Figure S54: *Significant clusters across cohort-wise analyses (replicability analysis) for* ***model 13*** *– CTQ extreme severity compared to none in HC and MDD samples without controlling for MDD diagnosis –* ***sex-stratified for male subsample***

Figure S55: *Significant clusters across cohort-wise analyses (replicability analysis) for* ***model 14*** *– CTQ extreme severity compared to none in HC subsamples –* ***sex-stratified for male subsample***

Figure S56: *Significant clusters across cohort-wise analyses (replicability analysis) for* ***model 15*** *– CTQ extreme severity compared to none in MDD subsamples –* ***sex-stratified for male subsample***

Figure S57: *Significant clusters across cohort-wise analyses (replicability analysis) for* ***model 16*** *– CTQ sum as a predictor in medication-naïve MDD subsamples –* ***sex-stratified for male subsample***

Figure S58: *Significant clusters across cohort-wise analyses (replicability analysis) for* ***model 17*** *– interaction term between CTQ sum and age as a predictor in HC subsamples –* ***sex-stratified for male subsample***

Figure S59: *Significant clusters across cohort-wise analyses (replicability analysis) for* ***model 18*** *– interaction term between CTQ sum and age as a predictor in MDD subsamples –* ***sex-stratified for male subsample***

Figure S60: *Permuted null distribution of significance overlap at uncorrected thresholds of* $p<.01$ *and* $p<.001$*, exemplary for Model 1 (not stratified by sex)*

Cohort characteristics and inclusion criteria

The existing Marburg-Münster Affective Disorders Cohort Study (MACS), Münster Neuroimaging Cohort (MNC) and BiDirect study were utilized for analysis. The cohorts include adult participants with age 18-65 years (35-65 years for the BiDirect study) and were all created to investigate affective disorders. Largest groups within each of the three cohorts comprise healthy control participants (HC) free from any lifetime history of mental disorders and participants with a major depressive disorder (MDD) diagnosis. Additional smaller patient groups with bipolar disorder (MACS and MNC), psychosis spectrum disorders (schizoaffective and schizophrenia; MACS), as well as comorbid cardiovascular diseases (BiDirect) were specifically recruited for the cohorts, and psychiatric comorbidities were generally permitted with some exceptions regarding brain organic disorders or substance use disorders. MACS and BiDirect recruited patient populations from psychiatric hospitals and outpatient centers in and around Münster, Germany (MACS and BiDirect) and Marburg, Germany (MACS). For the MNC, inpatient populations were included only, recruited also at psychiatric hospitals in Münster, Germany.

Image preprocessing for parcellation-based regional measures

Automated segmentation of the T1-weighted anatomical images was conducted using the Freesurfer (version 5.3; https://surfer.nmr.mgh.harvard.edu/; Fischl et al., 2002). Obtained regional segmentations were quality-checked visually by trained personnel. Poor segmentation of a region led to a case-wise exclusion from the analysis. Cortical parcellations were done based on the Desikan-Killiany atlas (Desikan et al., 2006) obtaining cortical surface and cortical thickness measures, each for a total of 68 regions, while subcortical volumes were obtained for 14 regions based on the Aseg atlas (Fischl et al., 2002). This resulted in a total of 150 regional measures of gray matter structure. In addition, mean cortical thickness and cortical surface were calculated for the left and right hemispheres respectively.

Scanner harmonization procedure

Data from different MRI scanners was included in the current analysis. In addition, data collection of the available cohorts took place over the course of several years (MNC being the oldest and still ongoing study, with study start in October 2009). Thus, hardware changes had to be conducted over the course of the study within the MACS and MNC study, potentially contributing to further scanner hardware effects. Notably, the three cohorts utilized here were already closely aligned with regard to scanning sequence parameters, thus reducing scanner effects to a minimum. The neuroCombat harmonization tool aims to neutralize scanner effects by estimating regional location (i.e., mean) and scaling (i.e., variance) parameters across all scanner groups (Fortin et al., 2018; https://github.com/Jfortin1/neuroCombat). By default, these parameters estimated using empirical bayes, thus pooling information across voxels. These parameters are then applied to adjust voxel intensities to achieve comparable distributions across scanner groups. This procedure allows the specification of “biological covariates”, for which associated variability will be preserved from the harmonization. This is particularly important when scanner groups differ with regard to variables that are associated with the biological outcome (e.g., age). As recommended in the Combat manual we included CTQ sum (as the primary variable of interest), as well as all utilized covariates (i.e., age, sex, total intracranial volume and MDD diagnosis) as biological covariates to avoid that the variability of gray matter intensities associated with these variables is ‘harmonized out’. The parcellation-based Freesurfer measures were harmonized in the same way as the voxel-based images. TIV was dropped

as a biological covariate for the harmonization of all cortical thickness measures. Voxel-wise harmonization was done using the neuroCombat toolbox in python (Combat version 0.2.12; python version 3.9.12), while the parcellation-based regional measures where harmonized using the neuroCombat implementation in R (Combat version 1.0.13; R version 4.3.0), as this version is able to handle case-wise missing values which were present in the parcellation-based as opposed to the voxel-wise data.

Measures for clinical characterization

A Structured Clinical Interview for the DSM-IV (SCID) (Wittchen et al., 1997) was used within MACS and MNC, while the Mini International Neuropsychiatric Interview (MINI) (Lecrubier et al., 1997) was used within the BiDirect cohort. Both measures, the SCID and the MINI, were used to deduct lifetime psychiatric diagnoses. The current remission status of MDD patients was assessed using the SCID for MACS and MNC. For the BiDirect cohort a CES-D cutoff was used as a proxy to categorize patients into remitted (CES-D < 20) or acute.

For additional clinical characterization regarding the current depression severity the Beck Depression Inventory (BDI) (Beck & Steer, 1987) was available for MACS and MNC cohorts, while the Center of Epidemiological Studies Depression Scale (CES-D) (Eaton et al., 2004) was available for the BiDirect cohort. The 17-item version of the Hamilton Depression Rating Scale (HDRS-17) was available in all three cohorts as a standardized clinical rating instrument for depression severity (Hamilton, 1960). Further, an additional interview-based characterization of the previous course of disease was available for MACS and MNC regarding estimates for the total number of psychiatric hospitalization and the number of previous depressive episodes. No comparable measure for the previous disease course was available for the BiDirect sample. For a summary of the clinical characterization of all cohorts see Table S1.

References

Beck, A. T., & Steer, R. A. (1987). *Beck Depression Inventory: manual*. The Psychological Corporation, Harcourt Brace Jovanovich.

Benjamini, Y., & Hochberg, Y. (1995). Controlling the False Discovery Rate: A Practical and Powerful Approach to Multiple Testing. *Journal of the Royal Statistical Society*, *57*(1), 289–300.

Desikan, R. S., Ségonne, F., Fischl, B., Quinn, B. T., Dickerson, B. C., Blacker, D., Buckner, R. L., Dale, A. M., Maguire, R. P., Hyman, B. T., Albert, M. S., & Killiany, R. J. (2006). An automated labeling system for subdividing the human cerebral cortex on MRI scans into gyral based regions of interest. *NeuroImage*, *31*(3), 968–980. https://doi.org/10.1016/j.neuroimage.2006.01.021

Eaton, W. W., Muntaner, C., Smith, C., Tien, A., & Ybarra, M. (2004). Center for epidemiologic studies depression scale: Review and revision. In M. E. Maruish (Ed.), *The use of psychological testing for treatment planning and outcomes assessment*. Lawrence Erlbaum. https://doi.org/10.1037/10517-024

Fischl, B., Salat, D. H., Busa, E., Albert, M. S., Dieterich, M., Haselgrove, C., van der Kouwe, A., Killiany, R., Kennedy, D., Klaveness, S., Montillo, A., Makris, N., Rosen, B., & Dale, A. M. (2002). Whole Brain Segmentation: Automated Labeling of Neuroanatomical Structures in the Human Brain. *Neuron*, *33*(3), 341–355. https://doi.org/10.1016/S0896-6273(02)00569-X

Fortin, J. P., Cullen, N., Sheline, Y. I., Taylor, W. D., Aselcioglu, I., Cook, P. A., Adams, P., Cooper, C.,

Fava, M., McGrath, P. J., McInnis, M., Phillips, M. L., Trivedi, M. H., Weissman, M. M., & Shinohara, R. T. (2018). Harmonization of cortical thickness measurements across scanners and sites. *NeuroImage*, *167*, 104–120. https://doi.org/10.1016/j.neuroimage.2017.11.024

Hamilton, M. (1960). A rating scale for depression. *Journal of Neurology, Neurosurgery, and Psychiatry*, *23*, 56–62.

Lecrubier, Y., Sheehan, D. V, Weiller, E., Amorim, P., Bonora, I., Harnett Sheehan, K., Janavs, J., & Dunbar, G. C. (1997). The Mini International Neuropsychiatric Interview (MINI). A short diagnostic structured interview: reliability and validity according to the CIDI. *European Psychiatry*, *12*(5), 224–231. https://doi.org/https://doi.org/10.1016/S0924-9338(97)83296-8

Notter, M., Gale, D., Herholz, P., Markello, R., Notter-Bielser, M.-L., & Whitaker, K. (2019). AtlasReader: A Python package to generate coordinate tables, region labels, and informative figures from statistical MRI images. *Journal of Open Source Software*, *4*(34), 1257. https://doi.org/10.21105/joss.01257

Wittchen, H.-U., Wunderlich, U., Gruschwitz, S., & Zaudig, M. (1997). *SKID-I. Strukturiertes Klinisches Interview für DSM-IV*. Hogrefe.

Table S1

Demographic and clinical characteristics across all three cohorts

	HC			MDD		
	count	mean	SD	count	mean	SD
MACS (n=1752)						
Age	930	34.23	12.96	822	36.50	13.13
Sex (f/m)	597/333			537/285	-	-
Education years	927	13.98	2.57	818	13.20	2.71
Remission (y/n)	-	-	-	452/369	-	-
HDRS-17	926	1.38	2.04	819	8.43	6.47
BDI	926	4.11	4.28	811	17.59	11.12
CES-D	-	-	-	-	-	-
Antidepressant medication (y/n)	0/930	-	-	488/334	-	-
Age of onset	-	-	-	817	25.82	12.49
# Depressive episodes	-	-	-	782	3.87	6.08
# Inpatient treatments	-	-	-	813	1.55	1.98
	count	mean	SD	count	mean	SD
MNC (n=916)						
Age	647	34.79	12.12	269	36.99	13.36
Sex (f/m)	359/288	-	-	148/121	-	-
Education years	643	14.64	2.51	268	13.73	2.71
Remission (y/n)	-	-	-	1/268	-	-
HDRS-17	520	0.85	1.40	267	19.21	4.12
BDI	647	2.32	3.12	266	28.20	9.14
CES-D	-	-	-	-	-	-
Antidepressant medication (y/n)	0/644	-	-	230/39	-	-
Age of onset	-	-	-	260	25.56	12.78
# Depressive episodes	-	-	-	269	4.42	5.39
# Inpatient treatments	-	-	-	249	2.45	2.71
	count	mean	SD	count	mean	SD
BiDirect (n=557)						
Age	321	52.61	7.97	236	50.63	7.06
Sex (f/m)	171/150	-	-	143/93	-	-
Education years	321	15.21	2.59	234	14.35	2.40
Remission (y/n)	-	-	-	75/161	-	-
HDRS-17	-	-	-	236	13.08	6.61
BDI	-	-	-	-	-	-
CES-D	321	7.84	5.90	236	26.33	12.36
Antidepressant medication (y/n)	2/319	-	-	202/34	-	-
Age of onset	-	-	-	-	-	-
# Depressive episodes	-	-	-	225	5.89	9.49
# Inpatient treatments	-	-	-	233	1.56	1.30

Note. HC, healthy controls; MDD, major depressive disorder; MACS, Marburg Münster Affective Disorders Cohort Study; MNC, Münster Neuroimaging Cohort; BiDirect, BiDirect study cohort; f, female; m, male; y, yes; n, no; HDRS-17, 17-item Hamilton Depression Rating Scale; BDI, Beck Depression Inventory; CES-D, Center for Epidemiologic Studies Depression Scale.

Table S2

Summary statistics for reported experiences of childhood maltreatment in the CTQ across cohorts

	HC			MDD		
	count	mean	SD	count	mean	SD
MACS (n=1725)						
CTQ sum	930	32.68	8.82	822	45.13	15.65
EA subscale sum	930	7.06	2.88	822	10.96	5.11
PA subscale sum	930	5.60	1.54	822	6.78	3.14
SA subscale sum	930	5.29	1.48	822	6.38	3.43
EN subscale sum	930	8.53	3.72	822	13.18	5.36
PN subscale sum	930	6.20	1.89	822	7.82	3.09
	count	mean	SD	count	mean	SD
MNC (n=916)						
CTQ sum	647	32.90	8.14	269	46.91	17.06
EA subscale sum	647	6.98	2.70	269	11.16	5.44
PA subscale sum	647	5.59	1.72	269	7.40	3.90
SA subscale sum	647	5.22	0.94	269	6.46	3.82
EN subscale sum	647	8.80	3.64	269	13.60	5.54
PN subscale sum	647	6.31	2.00	269	8.29	3.27
	count	mean	SD	count	mean	SD
BiDirect (n=557)						
CTQ sum	321	32.69	7.64	236	44.97	17.14
EA subscale sum	321	6.42	2.17	236	10.06	5.20
PA subscale sum	321	5.85	1.51	236	6.90	3.45
SA subscale sum	321	5.19	0.86	236	6.36	3.59
EN subscale sum	321	8.95	3.97	236	13.88	5.68
PN subscale sum	321	6.27	1.97	236	7.78	3.00

Note. CTQ, Childhood Trauma Questionnaire; HC, healthy controls; MDD, major depressive disorder; MACS, Marburg Münster Affective Disorders Cohort Study; MNC, Münster Neuroimaging Cohort; BiDirect, BiDirect study cohort; EA, emotional abuse; PA, physical abuse; SA, sexual abuse; PN, physical neglect; EN, emotional neglect.

Table S3

Pairwise cohort comparison in sample characteristics including HC and MDD participants

	MACS-MNC		MACS-BiDirect		MNC-BiDirect	
	Test statistic	p-value	Test statistic	p-value	Test statistic	p-value
Sex	21.9432	2.81E-06	25.7993	3.79E-07	0.9449	.331
MDD diagnosis	75.9345	2.93E-18	3.3405	.068	25.4202	4.61E-07
Age	813601	.554	811138	5.15E-123	79976	1.84E-108
Education years	934558	3.72E-15	599155	2.16E-18	235718	.024
CTQ sum	753976	0.010	475857	.378	245900	.244
CTQ EA	727820	6.20E-05	414523	5.39E-08	271148	.038
CTQ PA	772544	.054	521764	.004	227639	3.21E-05
CTQ SA	776172	.021	476570	.176	252777	.605
CTQ EN	763920	.041	501968	.304	235560	.013
CTQ PN	788585	.445	482996	.707	253297	.811

Note. Cohort differences in the distribution of categorical variables (sex and MDD diagnosis) were tested using chi^2 tests (the chi^2 value is given as a test statistic). Cohort mean differences in continuous variables were tested using Mann-Whitney-U tests (U-values are given as test statistic) due to normality violations in all continuous variables according to Shapiro-Wilk tests significant at $p<.05$. MDD, major depressive disorder; MACS, Marburg Münster Affective Disorders Cohort Study; MNC, Münster Neuroimaging Cohort; BiDirect, BiDirect study cohort; EA, emotional abuse; PA, physical abuse; SA, sexual abuse; PN, physical neglect; EN, emotional neglect.

Table S4

Pairwise cohort comparison of clinical characteristics of the MDD subgroups

	MACS-MNC		MACS-BiDirect		MNC-BiDirect	
	Test statistic	p-value	Test statistic	p-value	Test statistic	p-value
Remission	247.2073	1.06E-55	38.7962	4.70E-10	94.5569	2.38E-22
HDRS-17	20555.5	1.06E-88	58872	4.78E-20	48559	8.60E-26
BDI	50433	6.33E-39	-	-	-	-
Age of onset	108473	.6044	-	-	-	-
# Depressive episodes	91229	8.66E-04	75183	6.35E-04	29558.5	0.651
# Inpatient treatments	67923.5	3.77E-16	82246	.001	36381	1.28E-07

Note. Cohort differences in the distribution of categorical variables (remission) were tested using chi^2 tests (the chi^2 value is given as a test statistic). Cohort mean differences in continuous variables were tested using Mann-Whitney-U tests (U-values are given as test statistic) due to normality violations in all continuous variables according to Shapiro-Wilk tests significant at $p<.05$. CES-D values were not compared across cohorts as they were only available for the BiDirect cohort. MDD, major depressive disorder; MACS, Marburg Münster Affective Disorders Cohort Study; MNC, Münster Neuroimaging Cohort; BiDirect, BiDirect study cohort; HDRS-17, 17-item Hamilton Depression Rating Scale; BDI, Beck Depression Inventory.

Table S5

Differences in CTQ scales across HC and MDD groups in pooled sample and cohort-wise

Cohort	CTQ scale	U Statistic	p-value	Effect size r
Pooled	CTQ sum	569455.5	3.40E-155	0.548
	CTQ EA	628867.5	7.23E-134	0.501
	CTQ PA	974777.5	2.01E-39	0.226
	CTQ SA	1052632.5	3.84E-40	0.164
	CTQ EN	608515.5	3.57E-139	0.517
	CTQ PN	794500	1.11E-77	0.369
MACS	CTQ sum	169297	1.82E-90	0.557
	CTQ EA	187176	6.50E-78	0.510
	CTQ PA	296571	1.68E-22	0.224
	CTQ SA	315886.5	6.26E-24	0.174
	CTQ EN	181692	6.13E-81	0.525
	CTQ PN	241310	5.62E-44	0.369
MNC	CTQ sum	37554.5	5.22E-42	0.568
	CTQ EA	42979.5	8.49E-35	0.506
	CTQ PA	62214	1.14E-17	0.285
	CTQ SA	73644	6.86E-11	0.154
	CTQ EN	41962	2.29E-35	0.518
	CTQ PN	51077	6.12E-25	0.413
BiDirect	CTQ sum	18761	2.07E-24	0.505
	CTQ EA	20450	1.26E-21	0.460
	CTQ PA	32585.5	0.001	0.140
	CTQ SA	32396	4.64E-07	0.145
	CTQ EN	18389	1.93E-25	0.515
	CTQ PN	24819	3.42E-13	0.345

Note. Mann-Whitney U tests were calculated to test if differences in CTQ scales were significant across HC and MDD groups. A rank-biserial correlation coefficient is given as an effect size for this difference.

Table S6

Results summary for pooled cohorts (n=3225) at a significance level of p_{FWE}<.05 – ***sex-stratified for female subsample***

		Partial R^2		
Model	k significant	Min	Max	Main regions
Model 1	0	-	-	-
Model 2	632	0.010	0.014	Temporal Mid/Sup R, Cerebellum R, Insula L
Model 3	212	0.017	0.021	Insula L+R, Temporal Inf Orb 2 L, Temporal Pole Sup R, Temporal Mid/Sup R
Model 4	0	-	-	-
Model 5	0	-	-	-
Model 6	0	-	-	-
Model 7	0	-	-	-
Model 8	0	-	-	-
Model 9	0	-	-	-
Model 10	0	-	-	-
Model 11	0	-	-	-
Model 12	1847	0.017	0.025	Cerebellum L+R, Temporal Mid L, Rectus R, Caudate R, Insula R, Temporal Pole Sup R
Model 13	6343	0.017	0.029	Temporal Sup L+R, Temporal Pole Sup R, Lingual L, Amygdala R, Rectus L, Lingual R
Model 14	566	0.029	0.041	Insula L+R, Cerebellum L, Temporal Mid L, Frontal Inf Orb 2 L, Temporal Mid/Sup L+R
Model 15	15	0.041	0.045	'no label'
Model 16	0	-	-	-
Model 17	0	-	-	-
Model 18	0	-	-	-

Note. Number of significant voxels are shown, as well as their minimum and maximum effect size for each analysis. Cluster labelling was conducted based on the aal atlas using the atlasreader python package (Notter et al., 2019). Main regions are reported. Results were derived from general linear models with a one-sided test of the respective maltreatment predictor, at p_{FWE}<.05. L, left; R, right; Mid, middle; Sup, superior; Inf, inferior; Orb orbital.

Table S7

Results summary for pooled cohorts (n=3225) at a significance level of p_{FWE}<.05 – ***sex-stratified for male subsample***

		partial R^2		
Model	k significant	Min	Max	Main regions
Model 1	0	-	-	-
Model 2	312	0.015	0.020	Fusiform L+R, Lingual L, Temporal Sup R
Model 3	0	-	-	-
Model 4	0	-	-	-
Model 5	0	-	-	-
Model 6	0	-	-	-
Model 7	0	-	-	-
Model 8	0	-	-	-
Model 9	0	-	-	-
Model 10	0	-	-	-
Model 11	0	-	-	-
Model 12	0	-	-	-
Model 13	9	0.028	0.030	Fusiform L
Model 14	0	-	-	-
Model 15	0	-	-	-
Model 16	0	-	-	-
Model 17	0	-	-	-
Model 18	0	-	-	-

Note. Number of significant voxels are shown, as well as their minimum and maximum effect size for each analysis. Cluster labelling was conducted based on the aal atlas using the atlasreader python package (Notter et al., 2019). Main regions are reported. Results were derived from general linear models with a one-sided test of the respective maltreatment predictor, at p_{FWE}<.05. L, left; R, right; Sup, superior.

Table S8

Results summary of single cohorts at a cohort-wise significance level of $\boldsymbol{p_{unc}<.001}$

Model	Cohort	k significant	Partial R^2 Min	Partial R^2 Max	Main regions
Model 1	MACS	0	-	-	-
Model 1	MNC	8	0.01	0.011	Precentral L, Supramarginal R
Model 1	BiDirect	1299	0.017	0.029	Cerebellum L, Temporal Sup R
Model 2	MACS	1614	0.005	0.011	Temporal Mid/Sup R, Rectus R
Model 2	MNC	378	0.01	0.018	Thalamus L, Hippocampus L
Model 2	BiDirect	3388	0.017	0.037	Cerebellum L, Lingual L
Model 3	MACS	63	0.01	0.014	Frontal Mid 2 R, Frontal Inf Oper R
Model 3	MNC	646	0.015	0.026	Precentral L, Cerebellum R
Model 3	BiDirect	36	0.03	0.032	Rectus L, Cerebellum L
Model 4	MACS	42	0.012	0.014	Cerebellum R, Cerebellum L
Model 4	MNC	0	-	-	-
Model 4	BiDirect	630	0.04	0.071	Cerebellum L, Temporal Sup R
Model 5	MACS	0	-	-	-
Model 5	MNC	5	0.011	0.011	Postcentral R
Model 5	BiDirect	377	0.017	0.023	Cerebellum L, Temporal Sup R
Model 6	MACS	0	-	-	-
Model 6	MNC	38	0.01	0.014	Supramarginal R
Model 6	BiDirect	1461	0.017	0.028	Cerebellum L, Fusiform R
Model 7	MACS	0	-	-	-
Model 7	MNC	0	-	-	-
Model 7	BiDirect	375	0.017	0.023	Cerebellum L, Precuneus L
Model 8	MACS	282	0.005	0.007	Cerebellum R, Precentral R
Model 8	MNC	63	0.01	0.012	Calcarine L, Postcentral R
Model 8	BiDirect	1283	0.017	0.026	Cerebellum R, Precentral L
Model 9	MACS	0	-	-	-
Model 9	MNC	102	0.01	0.012	Frontal Mid 2 R, Precentral R
Model 9	BiDirect	491	0.017	0.024	Cerebellum L, Lingual L
Model 10	MACS	21	0.005	0.006	Cerebellum R
Model 10	MNC	10	0.01	0.013	Supramarginal R
Model 10	BiDirect	356	0.017	0.026	Cerebellum L, Temporal Mid L
Model 11	MACS	0	-	-	-
Model 11	MNC	29	0.01	0.011	'no label'
Model 11	BiDirect	3233	0.017	0.032	Cerebellum L, Supramarginal R
Model 12	MACS	0	-	-	-
Model 12	MNC	246	0.019	0.025	Frontal Sup 2 R, Cerebellum R
Model 12	BiDirect	956	0.031	0.042	Cerebellum L, Supramarginal R
Model 13	MACS	2403	0.01	0.017	Temporal Sup R, Rectus R
Model 13	MNC	675	0.019	0.033	Thalamus R, Temporal Sup R
Model 13	BiDirect	3031	0.031	0.057	Cerebellum L, Supramarginal R
Model 14	MACS	45	0.017	0.02	Temporal Inf R, Lingual L
Model 14	MNC	481	0.026	0.037	Temporal Sup R, Cerebellum R

Model 14	BiDirect	146	0.052	0.074	Rectus L, Precentral L
Model 15	MACS	59	0.022	0.027	Cerebellum L
Model 15	MNC	1135	0.064	0.135	Frontal Sup 2 R, Rectus R
Model 15	BiDirect	261	0.074	0.113	Frontal Inf Orb 2 R, Parietal Sup R
Model 16	MACS	0	-	-	-
Model 16	MNC	48	0.236	0.286	Temporal Mid L, Occipital Mid L
Model 16	BiDirect	90	0.269	0.362	Angular R, Occipital Mid R
Model 17	MACS	200	0.012	0.020	Lingual L, Precentral L
Model 17	MNC	102	0.017	0.023	Parietal Inf R, Temporal Inf R
Model 17	BiDirect	462	0.034	0.051	Temporal Mid L, Temporal Mid R
Model 18	MACS	1039	0.013	0.024	Temporal Mid L, Temporal Mid R
Model 18	MNC	184	0.040	0.053	Cingulate Mid R, Lingual L
Model 18	BiDirect	1938	0.046	0.079	Precuneus R, Angular L

Note. Number of significant voxels are shown, as well as their minimum and maximum effect size for each analysis. Cluster labelling was conducted based on the aal atlas using the atlasreader python package (Notter et al., 2019). Main regions of largest clusters are reported. Results were derived from general linear models with a one-sided test of the respective maltreatment predictor, at uncorrected $p<.001$. MACS, Marburg Münster Affective Disorders Cohort Study; MNC, Münster Neuroimaging Cohort; BiDirect, BiDirect study cohort.

Table S9

Results summary of single cohorts at a cohort-wise significance level of ***p_{unc}<.001 – sex-stratified for female subsample***

Model	Cohort	k significant	Partial R^2		Main regions
			Min	Max	
Model 1	MACS	0	-	-	-
Model 1	MNC	60	0.019	0.023	Lingual R, Cerebellum R
Model 1	BiDirect	535	0.033	0.051	Temporal Inf L, Cerebellum L
Model 2	MACS	1367	0.008	0.017	Temporal Sup R, Cerebellum R
Model 2	MNC	270	0.019	0.025	Precentral L, Lingual L
Model 2	BiDirect	1001	0.032	0.050	Cerebellum L, Lingual L
Model 3	MACS	358	0.016	0.027	Temporal Mid/Sup L, Frontal Mid 2 R
Model 3	MNC	1838	0.027	0.051	Temporal Sup R, Temporal Pole Sup L
Model 3	BiDirect	26	0.063	0.070	Parahippocampal R, Temporal Mid L
Model 4	MACS	0	-	-	-
Model 4	MNC	70	0.064	0.072	Postcentral R, Occipital Mid L
Model 4	BiDirect	263	0.066	0.086	OFC Post R, Frontal Inf Orb 2 R
Model 5	MACS	0	-	-	-
Model 5	MNC	192	0.019	0.032	Frontal Mid 2 R, Temporal Pole Sup R
Model 5	BiDirect	128	0.033	0.041	Temporal Sup R, Temporal Inf L
Model 6	MACS	65	0.008	0.010	Temporal Sup R
Model 6	MNC	116	0.019	0.023	Rectus R, Lingual R
Model 6	BiDirect	628	0.033	0.050	OFC Post R, Temporal Inf L
Model 7	MACS	0	-	-	-
Model 7	MNC	284	0.019	0.025	Frontal Mid 2 R, Temporal Pole Sup R
Model 7	BiDirect	247	0.033	0.044	OFC Post R, Temporal Mid R
Model 8	MACS	99	0.008	0.010	Cerebellum L, Precentral R
Model 8	MNC	960	0.019	0.030	Calcarine L, Cuneus L
Model 8	BiDirect	474	0.033	0.051	Paracentral Lobule R, Postcentral R
Model 9	MACS	0	-	-	-
Model 9	MNC	42	0.019	0.024	Thalamus R
Model 9	BiDirect	1045	0.033	0.051	Cerebellum R, Parietal Inf L
Model 10	MACS	393	0.008	0.012	Temporal Sup R
Model 10	MNC	116	0.019	0.023	Rectus R, Cingulate Mid L
Model 10	BiDirect	317	0.033	0.047	OFC Post R, Frontal Inf Orb 2 R
Model 11	MACS	0	-	-	-
Model 11	MNC	164	0.019	0.026	Occipital Inf R, Fusiform R
Model 11	BiDirect	1037	0.033	0.060	Frontal Inf Orb 2 L, Temporal Sup R
Model 12	MACS	819	0.015	0.022	Cerebellum L, Cerebellum R
Model 12	MNC	1381	0.033	0.051	Parietal Sup L, Cerebellum L
Model 12	BiDirect	1107	0.054	0.084	Cerebellum L, Temporal Inf L
Model 13	MACS	5010	0.015	0.031	Temporal Sup R, Cerebellum R
Model 13	MNC	209	0.033	0.047	Cerebellum R, Precentral L
Model 13	BiDirect	1133	0.054	0.081	Cerebellum L, Supramarginal R
Model 14	MACS	101	0.026	0.035	Insula R, Temporal Mid L
Model 14	MNC	1023	0.046	0.066	Temporal Sup R, Parietal Sup R
Model 14	BiDirect	1160	0.111	0.199	Temporal Inf R, Temporal Mid L
Model 15	MACS	8	0.033	0.036	'no label'

Model 15	MNC	269	0.111	0.162	Frontal Sup 2 R, Occipital Mid R
Model 15	BiDirect	865	0.103	0.149	Cerebellum L, Frontal Sup 2 R
Model 16	MACS	0	-	-	-
Model 16	MNC	35	0.359	0.416	Occipital Mid L, Temporal Inf R
Model 16	BiDirect	0	-	-	-
Model 17	MACS	22	0.018	0.021	Temporal Inf L, Lingual L
Model 17	MNC	87	0.030	0.041	Temporal Inf L, Occipital Inf R
Model 17	BiDirect	205	0.072	0.100	Occipital Mid R, Temporal Mid L
Model 18	MACS	223	0.020	0.029	Temporal Inf L, Temporal Sup R
Model 18	MNC	660	0.073	0.103	Parietal Inf L, Insula L
Model 18	BiDirect	145	0.075	0.116	Temporal Mid L, Temporal Inf R

Note. Number of significant voxels are shown, as well as their minimum and maximum effect size for each analysis. Cluster labelling was conducted based on the aal atlas using the atlasreader python package (Notter et al., 2019). Main regions of largest clusters are reported. Results were derived from general linear models with a one-sided test of the respective maltreatment predictor, at uncorrected p<.001. MACS, Marburg Münster Affective Disorders Cohort Study; MNC, Münster Neuroimaging Cohort; BiDirect, BiDirect study cohort.

Table S10

Results summary of single cohorts at a cohort-wise significance level of $\boldsymbol{p_{unc}}$***<.001 – sex-stratified for male subsample***

Model	Cohort	k significant	Partial R^2		Main regions
			Min	Max	
Model 1	MACS	10	0.015	0.017	Paracentral Lobule R
Model 1	MNC	87	0.023	0.033	Hippocampus L, Rectus L
Model 1	BiDirect	718	0.036	0.061	Postcentral R, Temporal Mid L
Model 2	MACS	522	0.015	0.022	Temporal Mid R, Paracentral Lobule R
Model 2	MNC	268	0.023	0.047	Rectus L, Thalamus L
Model 2	BiDirect	989	0.036	0.065	Postcentral R, Fusiform R
Model 3	MACS	71	0.029	0.034	Postcentral R, Calcarine L
Model 3	MNC	159	0.033	0.041	Cerebellum R
Model 3	BiDirect	163	0.055	0.075	Postcentral R, Cerebellum R
Model 4	MACS	0	-	-	-
Model 4	MNC	0	-	-	-
Model 4	BiDirect	1317	0.101	0.177	Precuneus R, Occipital Sup R
Model 5	MACS	110	0.015	0.021	Paracentral Lobule R
Model 5	MNC	92	0.023	0.028	Hippocampus L, Supramarginal R
Model 5	BiDirect	1766	0.036	0.074	Postcentral L, Cingulate Ant L
Model 6	MACS	47	0.016	0.023	Frontal Sup 2 L, Postcentral L
Model 6	MNC	23	0.023	0.029	Rectus L
Model 6	BiDirect	133	0.036	0.050	Postcentral R, Cerebellum R
Model 7	MACS	100	0.015	0.019	Temporal Sup R, Postcentral R
Model 7	MNC	26	0.023	0.034	Rectus L
Model 7	BiDirect	1441	0.036	0.064	Postcentral L, Temporal Mid L
Model 8	MACS	193	0.015	0.024	Paracentral Lobule R
Model 8	MNC	93	0.023	0.028	Thalamus L, Cerebellum L
Model 8	BiDirect	1945	0.036	0.069	Occipital Sup R, Precentral L
Model 9	MACS	0	-	-	-
Model 9	MNC	2219	0.023	0.037	Occipital Mid R, Supramarginal R
Model 9	BiDirect	1	0.036	0.036	Frontal Mid 2 R
Model 10	MACS	39	0.015	0.022	Frontal Sup 2 L
Model 10	MNC	4	0.024	0.025	'no label'
Model 10	BiDirect	20	0.036	0.038	Precuneus R, Occipital Mid L
Model 11	MACS	39	0.015	0.017	Postcentral R, Paracentral Lobule R
Model 11	MNC	0	-	-	-
Model 11	BiDirect	587	0.036	0.066	Cerebellum R, Postcentral R
Model 12	MACS	0	-	-	-
Model 12	MNC	154	0.043	0.050	Frontal Sup 2 R, Fusiform R
Model 12	BiDirect	345	0.071	0.112	Cuneus R, Frontal Inf Oper R
Model 13	MACS	7	0.028	0.030	Paracentral Lobule R, Cuneus L
Model 13	MNC	575	0.043	0.082	Rectus L, Thalamus L
Model 13	BiDirect	590	0.070	0.102	Lingual R, Frontal Inf Tri R
Model 14	MACS	106	0.050	0.070	Fusiform R, Occipital Mid L
Model 14	MNC	7	0.060	0.064	Temporal Inf R, Cerebellum R
Model 14	BiDirect	44	0.098	0.119	Insula L, Postcentral R
Model 15	MACS	0	-	-	-

Model 15	MNC	715	0.146	0.240	Frontal Sup 2 R, Frontal Sup Med L
Model 15	BiDirect	162	0.242	0.336	Occipital Sup R, Calcarine L
Model 16	MACS	4	0.082	0.087	Supp Motor Area R
Model 16	MNC	310	0.596	0.852	Frontal Inf Orb 2 L, Temporal Pole Sup L
Model 16	BiDirect	188	0.563	0.791	Precuneus R, Temporal Inf R
Model 17	MACS	59	0.032	0.043	Lingual L, Precentral R
Model 17	MNC	46	0.037	0.049	Parietal Inf R, Parahippocampal L
Model 17	BiDirect	140	0.063	0.079	Precentral L, Occipital Mid L
Model 18	MACS	547	0.038	0.054	Temporal Mid L, Frontal Sup 2 L
Model 18	MNC	105	0.089	0.102	Putamen L, Postcentral L
Model 18	BiDirect	12319	0.115	0.274	Precuneus R, Frontal Sup 2 R

Note. Number of significant voxels are shown, as well as their minimum and maximum effect size for each analysis. Cluster labelling was conducted based on the aal atlas using the atlasreader python package (Notter et al., 2019). Main regions of largest clusters are reported. Results were derived from general linear models with a one-sided test of the respective maltreatment predictor, at uncorrected $p<.001$. MACS, Marburg Münster Affective Disorders Cohort Study; MNC, Münster Neuroimaging Cohort; BiDirect, BiDirect study cohort.

Table S11

Results summary of single cohorts at a cohort-wise significance level of $\boldsymbol{p_{unc}<.01}$

Model	Cohort	k significant	Partial R^2		Main regions
			Min	Max	
Model 1	MACS	244	0.003	0.004	Cerebellum R, Temporal Sup R
Model 1	MNC	749	0.006	0.011	Precentral L, Supramarginal R
Model 1	BiDirect	10648	0.010	0.029	Cerebellum L, Supramarginal R
Model 2	MACS	10400	0.003	0.011	Temporal Sup R, Rectus R
Model 2	MNC	3444	0.006	0.018	Hippocampus L, Thalamus L
Model 2	BiDirect	16924	0.010	0.037	Cerebellum L, Supramarginal R
Model 3	MACS	1542	0.006	0.014	Frontal Sup 2 R, Frontal Sup Medial L
Model 3	MNC	5230	0.008	0.026	Cerebellum R, Precentral L
Model 3	BiDirect	2007	0.017	0.032	Cerebellum L, Temporal Mid L
Model 4	MACS	1018	0.007	0.014	Cerebellum L, Cerebellum R
Model 4	MNC	555	0.020	0.035	Occipital Mid L, Frontal Inf Tri R
Model 4	BiDirect	8650	0.023	0.071	Cerebellum L, Frontal Inf Orb 2 R
Model 5	MACS	309	0.003	0.004	Cerebellum L, Precentral R
Model 5	MNC	1501	0.006	0.011	Occipital Mid L, Precentral L
Model 5	BiDirect	8002	0.010	0.023	Cerebellum L, Temporal Sup R
Model 6	MACS	152	0.003	0.004	Cerebellum R, Temporal Sup R
Model 6	MNC	1251	0.006	0.014	Precentral L, Supramarginal R
Model 6	BiDirect	10527	0.010	0.028	Cerebellum L, Supramarginal R
Model 7	MACS	411	0.003	0.005	Precentral R, Temporal Sup R
Model 7	MNC	1052	0.006	0.010	Precentral L, Frontal Mid 2 R
Model 7	BiDirect	6301	0.010	0.023	Cerebellum L, Temporal Sup R
Model 8	MACS	4652	0.003	0.007	Cerebellum R, Cerebellum L
Model 8	MNC	3082	0.006	0.012	Calcarine L, Postcentral R
Model 8	BiDirect	13399	0.010	0.026	Cerebellum L, Postcentral L
Model 9	MACS	0	-	-	-
Model 9	MNC	2888	0.006	0.012	Frontal Mid 2 R, Thalamus L
Model 9	BiDirect	6123	0.010	0.024	Cerebellum L, Temporal Pole Mid R
Model 10	MACS	3234	0.003	0.006	Cerebellum L, Cerebellum R
Model 10	MNC	630	0.006	0.013	Precentral L, Frontal Mid 2 L
Model 10	BiDirect	6556	0.010	0.026	Cerebellum L, Cerebellum R
Model 11	MACS	25	0.003	0.004	Occipital Sup L, Calcarine L
Model 11	MNC	1919	0.006	0.011	Temporal Sup R, OFC Med L
Model 11	BiDirect	18007	0.010	0.032	Supramarginal R, Temporal Sup L
Model 12	MACS	833	0.005	0.010	Cerebellum R, Cerebellum L
Model 12	MNC	7423	0.011	0.025	Occipital Mid L, Supramarginal R
Model 12	BiDirect	7313	0.018	0.042	Cerebellum L, Supramarginal R
Model 13	MACS	18463	0.005	0.017	Temporal Sup/Mid R, Cerebellum L
Model 13	MNC	7885	0.011	0.033	Temporal Sup R, Caudate L
Model 13	BiDirect	14534	0.018	0.057	Cerebellum L, Supramarginal R
Model 14	MACS	1313	0.010	0.020	Temporal Inf R, Precuneus R
Model 14	MNC	8318	0.015	0.037	Caudate L, Temporal Sup R
Model 14	BiDirect	4534	0.030	0.074	Frontal Inf Tri L, Frontal Mid 2 L

Model 15	MACS	1182	0.012	0.027	Cerebellum L, Cerebellum R
Model 15	MNC	9257	0.037	0.135	Frontal Sup 2 R, Rectus R
Model 15	BiDirect	4980	0.043	0.113	Cerebellum L, Temporal Sup R
Model 16	MACS	5	0.016	0.017	no_label', Precuneus R
Model 16	MNC	1917	0.141	0.286	Temporal Mid L, Frontal Mid 2 R
Model 16	BiDirect	1189	0.163	0.362	Occipital Mid R, Angular R
Model 17	MACS	3318	0.007	0.020	Caudate L, Lingual L
Model 17	MNC	1256	0.010	0.023	Parietal Inf R, Fusiform R
Model 17	BiDirect	5585	0.021	0.051	Temporal Mid L, Cingulate Ant R
Model 18	MACS	7593	0.008	0.024	Temporal Sup R, Temporal Mid L
Model 18	MNC	2552	0.025	0.053	Cingulate Mid R, Frontal Inf Tri L
Model 18	BiDirect	15437	0.028	0.079	Precuneus R, Angular L

Note. Number of significant voxels are shown, as well as their minimum and maximum effect size for each analysis. Cluster labelling was conducted based on the aal atlas using the atlasreader python package (Notter et al., 2019). Main regions of largest clusters are reported. Results were derived from general linear models with a one-sided test of the respective maltreatment predictor, at uncorrected $p<.01$. MACS, Marburg Münster Affective Disorders Cohort Study; MNC, Münster Neuroimaging Cohort; BiDirect, BiDirect study cohort.

Table S12

Results summary of single cohorts at a cohort-wise significance level of ***p_{unc}<.01 – sex-stratified for female subsample***

			Partial R^2		
Model	Cohort	k significant	Min	Max	Main regions
Model 1	MACS	341	0.005	0.008	Temporal Sup R, Precentral R
Model 1	MNC	4962	0.011	0.023	Lingual R, Calcarine L
Model 1	BiDirect	6369	0.019	0.051	Cerebellum L, Temporal Sup R
Model 2	MACS	10960	0.005	0.017	Temporal Sup R, Cerebellum R
Model 2	MNC	5658	0.011	0.025	Lingual L, Precentral L
Model 2	BiDirect	8760	0.019	0.050	Temporal Sup R, Cerebellum L
Model 3	MACS	3885	0.009	0.027	Temporal Sup L, Frontal Mid 2 R
Model 3	MNC	15196	0.015	0.051	Cerebellum L, Temporal Sup R
Model 3	BiDirect	2355	0.036	0.070	Temporal Mid/Sup L, Lingual R
Model 4	MACS	364	0.010	0.017	Cerebellum R, Cerebellum R
Model 4	MNC	2183	0.037	0.072	Postcentral R, Occipital Mid L
Model 4	BiDirect	5523	0.038	0.086	OFC Post R, Temporal Sup R
Model 5	MACS	703	0.005	0.008	Cerebellum L, Precentral R
Model 5	MNC	5732	0.011	0.032	Frontal Mid 2 R, Temporal Pole Sup R
Model 5	BiDirect	4823	0.019	0.041	Temporal Sup R, Temporal Inf L
Model 6	MACS	890	0.005	0.010	Temporal Sup R, Cerebellum 3 L
Model 6	MNC	4008	0.011	0.023	Occipital Inf R, Cingulate Mid L
Model 6	BiDirect	7074	0.019	0.050	Cerebellum L, OFC Post R
Model 7	MACS	684	0.005	0.007	Cerebellum L, Cerebellum 8 R
Model 7	MNC	6563	0.011	0.025	Temporal Sup L, Frontal Mid 2 R
Model 7	BiDirect	5404	0.019	0.044	OFC Post R, Temporal Inf L
Model 8	MACS	3442	0.005	0.010	Cerebellum L, Cerebellum R
Model 8	MNC	8804	0.011	0.030	Calcarine L, Precentral L
Model 8	BiDirect	7382	0.019	0.051	Paracentral Lobule R, Temporal Sup L
Model 9	MACS	0	-	-	-
Model 9	MNC	2282	0.011	0.024	Thalamus L, Frontal Mid 2 R
Model 9	BiDirect	8756	0.019	0.051	Cerebellum L, Parietal Inf L
Model 10	MACS	2770	0.005	0.012	Temporal Sup R, Cerebellum 4 5 L
Model 10	MNC	3216	0.011	0.023	Cingulate Mid L, Rectus R
Model 10	BiDirect	4491	0.019	0.047	Cerebellum L, OFC Post R
Model 11	MACS	50	0.005	0.006	Temporal Pole Sup R, Precentral R
Model 11	MNC	4802	0.011	0.026	Occipital Inf R, Precuneus L
Model 11	BiDirect	15863	0.019	0.060	Temporal Sup R, OFC Post R
Model 12	MACS	5224	0.008	0.022	Cerebellum L, Temporal Mid L
Model 12	MNC	11483	0.019	0.051	Cerebellum L, Parietal Sup L
Model 12	BiDirect	6591	0.031	0.084	Cerebellum L, Temporal Inf L
Model 13	MACS	25060	0.008	0.031	Cerebellum L, Temporal Sup R
Model 13	MNC	5853	0.019	0.047	Precentral L, Parietal Sup L
Model 13	BiDirect	6656	0.031	0.081	Cerebellum L, Fusiform R
Model 14	MACS	4409	0.015	0.035	Cerebellum R, Temporal Mid L
Model 14	MNC	13393	0.026	0.066	Cerebellum L, Temporal Sup R
Model 14	BiDirect	7063	0.064	0.199	Temporal Inf/Mid R, Temporal Mid/Inf L
Model 15	MACS	2650	0.019	0.036	Cerebellum R, Cerebellum L

Model 15	MNC	6336	0.064	0.162	Postcentral R, Occipital Mid R
Model 15	BiDirect	4354	0.060	0.149	Cerebellum L, OFC Post R
Model 16	MACS	3	0.025	0.028	Temporal Mid L
Model 16	MNC	1617	0.222	0.416	Occipital Mid L, Postcentral R
Model 16	BiDirect	432	0.294	0.444	Temporal Mid R, Temporal Inf L
Model 17	MACS	2165	0.011	0.021	Caudate L, Lingual L
Model 17	MNC	2165	0.019	0.041	Temporal Inf L, Calcarine L
Model 17	BiDirect	4781	0.045	0.100	Temporal Mid L, Temporal Sup R
Model 18	MACS	3217	0.012	0.029	Temporal Sup R, Temporal Inf L
Model 18	MNC	5661	0.045	0.103	Rolandic Oper L, Parietal Inf L
Model 18	BiDirect	3706	0.047	0.116	Occipital Mid L, Frontal Sup 2 L

Note. Number of significant voxels are shown, as well as their minimum and maximum effect size for each analysis. Cluster labelling was conducted based on the aal atlas using the atlasreader python package (Notter et al., 2019). Main regions of largest clusters are reported. Results were derived from general linear models with a one-sided test of the respective maltreatment predictor, at uncorrected $p<.01$. MACS, Marburg Münster Affective Disorders Cohort Study; MNC, Münster Neuroimaging Cohort; BiDirect, BiDirect study cohort.

Table S13

Results summary of single cohorts at a cohort-wise significance level of $\boldsymbol{p_{unc}}$***<.01 – sex-stratified for male subsample***

Model	Cohort	k significant	Partial R²		Main regions
			Min	Max	
Model 1	MACS	707	0.009	0.017	Postcentral R, Paracentral Lobule R
Model 1	MNC	1452	0.013	0.033	Hippocampus L, Supramarginal R
Model 1	BiDirect	8774	0.021	0.061	Cerebellum R, Fusiform R
Model 2	MACS	5785	0.009	0.022	Paracentral Lobule R, Temporal Mid R
Model 2	MNC	3009	0.013	0.047	Hippocampus L, Thalamus L
Model 2	BiDirect	10655	0.021	0.065	Cerebellum R, Fusiform R
Model 3	MACS	2899	0.016	0.034	Occipital Mid L, Precentral R
Model 3	MNC	1445	0.019	0.041	Cerebellum R, Postcentral R
Model 3	BiDirect	2678	0.032	0.075	Postcentral R, Cerebellum R
Model 4	MACS	131	0.019	0.026	Supp Motor Area R, Temporal Mid R
Model 4	MNC	1190	0.045	0.073	Frontal Sup Med L, Supramarginal R
Model 4	BiDirect	12785	0.059	0.177	Occipital Sup R, Parietal Inf L
Model 5	MACS	792	0.009	0.021	Paracentral Lobule R, Cingulate Mid R
Model 5	MNC	2591	0.013	0.028	Hippocampus L, Cerebellum L
Model 5	BiDirect	14755	0.021	0.074	Cerebellum R, Postcentral L
Model 6	MACS	641	0.009	0.023	Postcentral L, Frontal Sup 2 L
Model 6	MNC	811	0.013	0.029	OFC Med L, Frontal Sup 2 R
Model 6	BiDirect	4054	0.021	0.050	Postcentral R, Cerebellum R
Model 7	MACS	2591	0.009	0.019	Temporal Mid R, Postcentral R
Model 7	MNC	676	0.013	0.034	Cerebellum L, Hippocampus L
Model 7	BiDirect	13369	0.021	0.064	Postcentral L, Precuneus R
Model 8	MACS	929	0.009	0.024	Paracentral Lobule R, Supp Motor Area R
Model 8	MNC	2202	0.013	0.028	Hippocampus L, Thalamus L
Model 8	BiDirect	14045	0.021	0.069	Postcentral L, Cerebellum L
Model 9	MACS	54	0.009	0.012	Paracentral Lobule R, Frontal Sup 2 L
Model 9	MNC	19142	0.013	0.037	Lingual L, Supramarginal R
Model 9	BiDirect	289	0.021	0.036	Frontal Mid 2 R, Frontal Mid 2 L
Model 10	MACS	1063	0.009	0.022	Cerebellum L, Fusiform R
Model 10	MNC	721	0.013	0.025	Lingual L, OFC Med L
Model 10	BiDirect	3344	0.021	0.038	Cerebellum R, Precuneus R
Model 11	MACS	2258	0.009	0.017	Paracentral Lobule R, Occipital Mid L
Model 11	MNC	573	0.013	0.022	OFC Med L, Hippocampus L
Model 11	BiDirect	7053	0.021	0.066	Cerebellum R, Cerebellum L
Model 12	MACS	26	0.016	0.019	Supramarginal R, Paracentral Lobule R
Model 12	MNC	3261	0.025	0.050	Frontal Sup 2 R, Lingual L
Model 12	BiDirect	4882	0.041	0.112	Occipital Sup R, Frontal Inf Tri R
Model 13	MACS	2624	0.016	0.030	Fusiform L, Paracentral Lobule R
Model 13	MNC	6234	0.024	0.082	Rectus L, Hippocampus L
Model 13	BiDirect	7067	0.040	0.102	Frontal Inf Tri R, Lingual R
Model 14	MACS	1203	0.029	0.070	Fusiform R, Lingual L
Model 14	MNC	2577	0.034	0.064	Cerebellum R, Thalamus R
Model 14	BiDirect	2641	0.056	0.119	Postcentral R, Occipital Inf R
Model 15	MACS	43	0.036	0.047	Supp Motor Area R, Postcentral L

Model 15	MNC	7320	0.086	0.240	Rectus L, Frontal Sup 2 R
Model 15	BiDirect	4788	0.145	0.336	Occipital Sup R, Cuneus L
Model 16	MACS	737	0.047	0.087	Frontal Sup 2 L, Frontal Sup Medial L
Model 16	MNC	4744	0.402	0.852	Frontal Mid 2 R, Temporal Pole Sup L
Model 16	BiDirect	3521	0.375	0.791	Precuneus R, Caudate L
Model 17	MACS	1678	0.020	0.043	Paracentral Lobule L, Precuneus L
Model 17	MNC	1717	0.023	0.049	Parahippocampal R, Parahippocampal L
Model 17	BiDirect	1963	0.039	0.079	Frontal Inf Oper L, Parietal Sup L
Model 18	MACS	6679	0.023	0.054	Frontal Sup 2 L, Temporal Mid L
Model 18	MNC	2660	0.055	0.102	Insula L, Postcentral L
Model 18	BiDirect	47660	0.072	0.274	Frontal Mid 2 L, Precuneus R

Note. Number of significant voxels are shown, as well as their minimum and maximum effect size for each analysis. Cluster labelling was conducted based on the aal atlas using the atlasreader python package (Notter et al., 2019). Main regions of largest clusters are reported. Results were derived from general linear models with a one-sided test of the respective maltreatment predictor, at uncorrected p<.01. MACS, Marburg Münster Affective Disorders Cohort Study; MNC, Münster Neuroimaging Cohort; BiDirect, BiDirect study cohort.

Table S14

Replicability across cohorts indicated by spatial overlap in significance at a cohort-wise level of $\boldsymbol{p_{unc}}$***<.001 – sex-stratified for female subsample***

	MACS-MNC				MACS-BiDirect				MNC-BiDirect				MACS-MNC-BiDirect			
Model	k overlap	p-value	p-value (FDR)	DICE	k overlap	p-value	p-value (FDR)	DICE	k overlap	p-value	p-value (FDR)	DICE	k overlap	p-value	p-value (FDR)	DICE
Model 1	0	1	1	0	0	1	1	0	0	1	1	0	0	1	1	-
Model 2	0	1	1	0	0	1	1	0	0	1	1	0	0	1	1	-
Model 3	0	1	1	0	0	1	1	0	0	1	1	0	0	1	1	-
Model 4	0	1	1	0	0	1	1	0	0	1	1	0	0	1	1	-
Model 5	0	1	1	0	0	1	1	0	0	1	1	0	0	1	1	-
Model 6	0	1	1	0	0	1	1	0	0	1	1	0	0	1	1	-
Model 7	0	1	1	0	0	1	1	0	0	1	1	0	0	1	1	-
Model 8	0	1	1	0	0	1	1	0	0	1	1	0	0	1	1	-
Model 9	0	1	1	0	0	1	1	0	0	1	1	0	0	1	1	-
Model 10	0	1	1	0	0	1	1	0	0	1	1	0	0	1	1	-
Model 11	0	1	1	0	0	1	1	0	0	1	1	0	0	1	1	-
Model 12	0	1	1	0	0	1	1	0	0	1	1	0	0	1	1	-
Model 13	0	1	1	0	0	1	1	0	0	1	1	0	0	1	1	-
Model 14	0	1	1	0	4	0.011	0.791	0.006	0	1	1	0	0	1	1	-
Model 15	0	1	1	0	0	1	1	0	0	1	1	0	0	1	1	-
Model 16	0	1	1	0	0	1	1	0	0	1	1	0	0	1	1	-
Model 17	0	1	1	0	0	1	1	0	0	1	1	0	0	1	1	-
Model 18	0	1	1	0	0	1	1	0	0	1	1	0	0	1	1	-

Note. The overlap in significant voxels is presented across all statistical models and all cohort combinations. The DICE score is presented for pairwise combinations with any voxels overlapping. Cohort-wise significance were derived from general linear models with a one-sided test of the respective maltreatment predictor, at uncorrected p<.001. P-values for overlap significance are based on permutation-based null distribution of cohort-wise overlap. FDR-correction was applied using the Benjamini-Hochberg procedure across all p-values in the table (Benjamini & Hochberg, 1995). MACS, Marburg Münster Affective Disorders Cohort Study; MNC, Münster Neuroimaging Cohort; BiDirect, BiDirect study cohort.

Table S15

Replicability across cohorts indicated by spatial overlap in significance at a cohort-wise level of ***p_{unc}<.001 – sex-stratified for male subsample***

	MACS-MNC				MACS-BiDirect				MNC-BiDirect				MACS-MNC-BiDirect			
Model	k overlap	p-value	p-value (FDR)	DICE	k overlap	p-value	p-value (FDR)	DICE	k overlap	p-value	p-value (FDR)	DICE	k overlap	p-value	p-value (FDR)	DICE
Model 1	0	1	1	0	0	1	1	0	0	1	1	0	0	1	1	-
Model 2	0	1	1	0	0	1	1	0	0	1	1	0	0	1	1	-
Model 3	0	1	1	0	0	1	1	0	0	1	1	0	0	1	1	-
Model 4	0	1	1	0	0	1	1	0	0	1	1	0	0	1	1	-
Model 5	0	1	1	0	0	1	1	0	0	1	1	0	0	1	1	-
Model 6	0	1	1	0	0	1	1	0	0	1	1	0	0	1	1	-
Model 7	0	1	1	0	0	1	1	0	0	1	1	0	0	1	1	-
Model 8	0	1	1	0	0	1	1	0	0	1	1	0	0	1	1	-
Model 9	0	1	1	0	0	1	1	0	0	1	1	0	0	1	1	-
Model 10	0	1	1	0	0	1	1	0	0	1	1	0	0	1	1	-
Model 11	0	1	1	0	0	1	1	0	0	1	1	0	0	1	1	-
Model 12	0	1	1	0	0	1	1	0	0	1	1	0	0	1	1	-
Model 13	0	1	1	0	0	1	1	0	0	1	1	0	0	1	1	-
Model 14	0	1	1	0	0	1	1	0	0	1	1	0	0	1	1	-
Model 15	0	1	1	0	0	1	1	0	0	1	1	0	0	1	1	-
Model 16	0	1	1	0	0	1	1	0	0	1	1	0	0	1	1	-
Model 17	0	1	1	0	0	1	1	0	0	1	1	0	0	1	1	-
Model 18	0	1	1	0	91	0.001	0.072	0.014	3	0.016	0.575	0.000	0	1	1	-

Note. The overlap in significant voxels is presented across all statistical models and all cohort combinations. The DICE score is presented for pairwise combinations with any voxels overlapping. Cohort-wise significance were derived from general linear models with a one-sided test of the respective maltreatment predictor, at uncorrected p<.001. P-values for overlap significance are based on permutation-based null distribution of cohort-wise overlap. FDR-correction was applied using the Benjamini-Hochberg procedure across all p-values in the table (Benjamini & Hochberg, 1995). MACS, Marburg Münster Affective Disorders Cohort Study; MNC, Münster Neuroimaging Cohort; BiDirect, BiDirect study cohort.

Table S16

Replicability across cohorts indicated by spatial overlap in significance at a cohort-wise level of $\boldsymbol{p_{unc}<.01}$

	MACS-MNC				MACS-BiDirect				MNC-BiDirect				MACS-MNC-BiDirect			
Model	k overlap	p-value	p-value (FDR)	DICE	k overlap	p-value	p-value (FDR)	DICE	k overlap	p-value	p-value (FDR)	DICE	k overlap	p-value	p-value (FDR)	DICE
Model 1	0	1	1	0	0	1	1	0	6	0.380	0.989	0.001	0	1	1	-
Model 2	73	0.132	0.678	0.011	329	0.038	0.248	0.024	223	0.032	0.248	0.022	4	0.010	0.144	-
Model 3	8	0.318	0.880	0.002	11	0.308	0.880	0.006	12	0.303	0.880	0.003	0	1	1	-
Model 4	0	1	1	0	0	1	1	0	0	1	1	0	0	1	1	-
Model 5	0	1	1	0	190	0.065	0.360	0.046	14	0.295	0.880	0.003	0	1	1	-
Model 6	0	1	1	0	0	1	1	0	18	0.292	0.880	0.003	0	1	1	-
Model 7	0	1	1	0	38	0.216	0.848	0.011	0	1	1	0	0	1	1	-
Model 8	50	0.190	0.825	0.013	286	0.037	0.248	0.032	17	0.262	0.880	0.002	0	1	1	-
Model 9	0	1	1	0	0	1	1	0	0	1	1	0	0	1	1	-
Model 10	0	1	1	0	48	0.185	0.825	0.010	0	1	1	0	0	1	1	-
Model 11	0	1	1	0	0	1	1	0	609	0.007	0.144	0.061	0	1	1	-
Model 12	0	1	1	0	0	1	1	0	346	0.026	0.234	0.047	0	1	1	-
Model 13	739	0.009	0.144	0.056	1329	0.004	0.144	0.081	472	0.019	0.195	0.042	12	0.013	0.156	-
Model 14	2	0.436	1	0.000	0	1	1	0	127	0.059	0.354	0.020	0	1	1	-
Model 15	36	0.224	0.848	0.007	0	1	1	0	46	0.195	0.825	0.006	0	1	1	-
Model 16	0	1	1	0	0	1	1	0	0	1	1	0	0	1	1	-
Model 17	4	0.558	1	0.007	0	1	1	0.006	15	0.385	0.989	0.003	0	1	1	-
Model 18	9	0.463	1	0.001	424	0.007	0.144	0.035	42	0.245	0.880	0.003	0	1	1	-

Note. The overlap in significant voxels is presented across all statistical models and all cohort combinations. The DICE score is presented for pairwise combinations with any voxels overlapping. Cohort-wise significance were derived from general linear models with a one-sided test of the respective maltreatment predictor, at uncorrected p<.01. P-values for overlap significance are based on permutation-based null distribution of cohort-wise overlap. FDR-correction was applied using the Benjamini-Hochberg procedure across all p-values in the table (Benjamini & Hochberg, 1995). MACS, Marburg Münster Affective Disorders Cohort Study; MNC, Münster Neuroimaging Cohort; BiDirect, BiDirect study cohort.

Table S17

Replicability across cohorts indicated by spatial overlap in significance at a cohort-wise level of ***p_{unc}<.01 – sex-stratified for female subsample***

	MACS-MNC				MACS-BiDirect				MNC-BiDirect				MACS-MNC-BiDirect			
Model	k overlap	p-value	p-value (FDR)	DICE	k overlap	p-value	p-value (FDR)	DICE	k overlap	p-value	p-value (FDR)	DICE	k overlap	p-value	p-value (FDR)	DICE
Model 1	0	1	1	0	0	1	1	0	0	1	1	0	0	1	1	-
Model 2	102	0.118	0.618	0.012	464	0.018	0.432	0.047	28	0.231	0.722	0.004	0	1	1	-
Model 3	51	0.168	0.618	0.005	0	1	1	0	0	1	1	0	0	1	1	-
Model 4	0	1	1	0	4	0.411	1	0.001	77	0.125	0.618	0.020	0	1	1	-
Model 5	0	1	1	0	215	0.049	0.524	0.078	38	0.189	0.618	0.007	0	1	1	-
Model 6	0	1	1	0	0	1	1	0	2	0.455	1	0.000	0	1	1	-
Model 7	0	1	1	0	153	0.067	0.535	0.050	64	0.160	0.618	0.011	0	1	1	-
Model 8	0	1	1	0	95	0.097	0.618	0.018	53	0.140	0.618	0.007	0	1	1	-
Model 9	0	1	1	0	0	1	1	0	0	1	1	0	0	1	1	-
Model 10	1	0.472	1	0.000	0	1	1	0	0	1	1	0	0	1	1	-
Model 11	0	1	1	0	0	1	1	0	44	0.183	0.618	0.004	0	1	1	-
Model 12	47	0.182	0.618	0.006	75	0.123	0.618	0.013	83	0.118	0.618	0.009	0	1	1	-
Model 13	185	0.060	0.535	0.012	1655	0.002	0.144	0.104	0	1	1	0	0	1	1	-
Model 14	239	0.032	0.524	0.027	430	0.014	0.432	0.075	172	0.051	0.524	0.017	0	1	1	-
Model 15	226	0.040	0.524	0.050	6	0.411	1	0.002	51	0.179	0.618	0.010	0	1	1	-
Model 16	0	1	1	0	0	1	1	0	0	1	1	0	0	1	1	-
Model 17	0	1	1	0	0	1	1	0	4	0.587	1	0.001	0	1	1	-
Model 18	65	0.163	0.618	0.015	13	0.412	1	0.004	42	0.251	0.752	0.009	0	1	1	-

Note. The overlap in significant voxels is presented across all statistical models and all cohort combinations. The DICE score is presented for pairwise combinations with any voxels overlapping. Cohort-wise significance were derived from general linear models with a one-sided test of the respective maltreatment predictor, at uncorrected p<.01. P-values for overlap significance are based on permutation-based null distribution of cohort-wise overlap. FDR-correction was applied using the Benjamini-Hochberg procedure across all p-values in the table (Benjamini & Hochberg, 1995). MACS, Marburg Münster Affective Disorders Cohort Study; MNC, Münster Neuroimaging Cohort; BiDirect, BiDirect study cohort.

Table S18

Replicability across cohorts indicated by spatial overlap in significance at a level of ***p_{unc}<.01 – sex-stratified for male subsample***

	MACS-MNC				MACS-BiDirect				MNC-BiDirect				MACS-MNC-BiDirect			
Model	k overlap	p-value	p-value (FDR)	DICE	k overlap	p-value	p-value (FDR)	DICE	k overlap	p-value	p-value (FDR)	DICE	k overlap	p-value	p-value (FDR)	DICE
Model 1	0	1	1	0	0	1	1	0	3	0.462	1	0.001	0	1	1	-
Model 2	93	0.102	1	0.021	144	0.090	1	0.018	47	0.195	1	0.007	0	1	1	-
Model 3	0	1	1	0	0	1	1	0	0	1	1	0	0	1	1	-
Model 4	0	1	1	0	0	1	1	0	4	0.418	1	0.001	0	1	1	-
Model 5	0	1	1	0	0	1	1	0	13	0.333	1	0.001	0	1	1	-
Model 6	0	1	1	0	0	1	1	0	9	0.372	1	0.004	0	1	1	-
Model 7	0	1	1	0	12	0.340	1	0.002	0	1	1	0	0	1	1	-
Model 8	0	1	1	0	3	0.436	1	0.000	128	0.089	1	0.016	0	1	1	-
Model 9	0	1	1	0	0	1	1	0	12	0.249	1	0.001	0	1	1	-
Model 10	0	1	1	0	0	1	1	0	0	1	1	0	0	1	1	-
Model 11	0	1	1	0	25	0.266	1	0.005	0	1	1	0	0	1	1	-
Model 12	0	1	1	0	0	1	1	0	76	0.123	1	0.019	0	1	1	-
Model 13	64	0.153	1	0.014	62	0.159	1	0.013	90	0.109	1	0.014	0	1	1	-
Model 14	0	1	1	0	0	1	1	0	0	1	1	0	0	1	1	-
Model 15	0	1	1	0	0	1	1	0	40	0.239	1	0.007	0	1	1	-
Model 16	0	1	1	0	0	1	1	0	0	1	1	0	0	1	1	-
Model 17	0	1	1	0	13	0.460	1	0.007	64	0.178	1	0.035	0	1	1	-
Model 18	13	0.401	1	0.003	949	0.003	0.216	0.035	409	0.014	0.503	0.016	0	1	1	-

Note. The overlap in significant voxels is presented across all statistical models and all cohort combinations. The DICE score is presented for pairwise combinations with any voxels overlapping. Cohort-wise significance were derived from general linear models with a one-sided test of the respective maltreatment predictor, at uncorrected p<.01. P-values for overlap significance are based on permutation-based null distribution of cohort-wise overlap. FDR-correction was applied using the Benjamini-Hochberg procedure across all p-values in the table (Benjamini & Hochberg, 1995). MACS, Marburg Münster Affective Disorders Cohort Study; MNC, Münster Neuroimaging Cohort; BiDirect, BiDirect study cohort.

Table S19

Results summary of pooled and single cohorts for parcellation-based Freesurfer analysis and global gray matter density analysis significant at $p_{FDR}<.05$

Sex-stratification	Cohort	Model	Region	t_value	partial R²	p-FDR
-	Pooled	Model 2	R_bankssts_thickavg	-3.443	0.004	0.011
-	Pooled	Model 2	R_middletemporal_thickavg	-3.718	0.005	0.005
-	Pooled	Model 2	R_bankssts_surfavg	-3.037	0.003	0.037
-	Pooled	Model 2	global gray matter density	-4.908	0.007	0
-	Pooled	Model 5	global gray matter density	-3.536	0.004	0.031
-	Pooled	Model 7	global gray matter density	-3.8	0.004	0.011
-	Pooled	Model 8	global gray matter density	-3.726	0.004	0.015
-	Pooled	Model 12	L_inferiorparietal_surfavg	-3.185	0.006	0.037
-	Pooled	Model 12	R_bankssts_surfavg	-3.353	0.007	0.031
-	Pooled	Model 12	global gray matter density	-3.778	0.008	0.012
-	Pooled	Model 13	R_bankssts_surfavg	-3.23	0.007	0.048
-	Pooled	Model 13	global gray matter density	-5.598	0.017	0
-	MACS	Model 2	global gray matter density	-3.138	0.006	0.044
-	MACS	Model 13	global gray matter density	-3.836	0.015	0.005
-	MNC	Model 2	L_precentral_thickavg	-3.658	0.017	0.02
-	MNC	Model 7	L_postcentral_thickavg	-3.634	0.017	0.022
-	MNC	Model 14	R_fusiform_surfavg	-3.344	0.036	0.032
-	BiDirect	Model 2	L_parsorbitalis_thickavg	-3.023	0.018	0.039
-	BiDirect	Model 2	L_precuneus_thickavg	-3.417	0.023	0.025
-	BiDirect	Model 2	R_entorhil_thickavg	-3.029	0.019	0.039
-	BiDirect	Model 2	R_fusiform_thickavg	-3.258	0.021	0.029
-	BiDirect	Model 4	L_lingual_thickavg	-3.336	0.05	0.044
-	BiDirect	Model 4	L_precuneus_thickavg	-3.256	0.047	0.044
-	BiDirect	Model 7	L_lingual_thickavg	-3.103	0.019	0.049
-	BiDirect	Model 7	L_precuneus_thickavg	-3.535	0.025	0.021
-	BiDirect	Model 7	R_parsorbitalis_thickavg	-3.464	0.024	0.021
female	Pooled	Model 2	R_bankssts_surfavg	-3.11	0.006	0.039
female	Pooled	Model 2	R_superiortemporal_surfavg	-3.067	0.006	0.039
female	Pooled	Model 2	global gray matter density	-4.637	0.011	0
female	Pooled	Model 12	Rpal	-3.418	0.012	0.024
female	Pooled	Model 12	global gray matter density	-4.727	0.02	0
female	Pooled	Model 13	Rpal	-3.312	0.011	0.036
female	Pooled	Model 13	L_inferiorparietal_surfavg	-3.041	0.009	0.043
female	Pooled	Model 13	R_bankssts_surfavg	-3.158	0.01	0.041
female	Pooled	Model 13	R_middletemporal_surfavg	-2.95	0.009	0.043
female	Pooled	Model 13	R_superiortemporal_surfavg	-2.934	0.009	0.043
female	Pooled	Model 13	global gray matter density	-5.982	0.031	0
female	Pooled	Model 14	Lcaud	-2.969	0.015	0.048
female	Pooled	Model 14	Rcaud	-2.96	0.015	0.048
female	Pooled	Model 14	L_insula_surfavg	-3.171	0.018	0.039
female	Pooled	Model 14	R_cuneus_surfavg	-3.511	0.022	0.017
female	Pooled	Model 14	R_parahippocampal_surfavg	-2.889	0.014	0.05

female	Pooled	Model 14	global gray matter density	-3.874	0.023	0.008
female	MACS	Model 13	global gray matter density	-3.754	0.021	0.014
female	MNC	Model 1	Lthickness	-3.029	0.021	0.016
female	MNC	Model 1	RThickness	-3.08	0.022	0.015
female	MNC	Model 1	L_paracentral_thickavg	-2.716	0.017	0.039
female	MNC	Model 1	L_postcentral_thickavg	-3.892	0.035	0.002
female	MNC	Model 1	L_precentral_thickavg	-4.217	0.04	0.001
female	MNC	Model 1	L_precuneus_thickavg	-3.779	0.033	0.002
female	MNC	Model 1	R_bankssts_thickavg	-3.064	0.023	0.015
female	MNC	Model 1	R_lingual_thickavg	-3.173	0.023	0.013
female	MNC	Model 1	R_postcentral_thickavg	-4.121	0.039	0.001
female	MNC	Model 1	R_precentral_thickavg	-3.803	0.033	0.002
female	MNC	Model 1	R_precuneus_thickavg	-3.932	0.036	0.002
female	MNC	Model 1	R_superiorparietal_thickavg	-3.225	0.024	0.012
female	MNC	Model 1	R_superiortemporal_thickavg	-2.617	0.017	0.049
female	MNC	Model 2	Rpal	-2.619	0.016	0.043
female	MNC	Model 2	Lthickness	-2.726	0.017	0.036
female	MNC	Model 2	RThickness	-11.847	0.245	0
female	MNC	Model 2	L_paracentral_thickavg	-2.858	0.019	0.025
female	MNC	Model 2	L_pericalcarine_thickavg	-2.689	0.017	0.037
female	MNC	Model 2	L_postcentral_thickavg	-3.21	0.024	0.017
female	MNC	Model 2	L_precentral_thickavg	-3.626	0.03	0.009
female	MNC	Model 2	L_precuneus_thickavg	-3.47	0.028	0.009
female	MNC	Model 2	R_cuneus_thickavg	-2.982	0.021	0.02
female	MNC	Model 2	R_lingual_thickavg	-3.431	0.027	0.009
female	MNC	Model 2	R_postcentral_thickavg	-3.441	0.027	0.009
female	MNC	Model 2	R_precentral_thickavg	-2.921	0.02	0.023
female	MNC	Model 2	R_precuneus_thickavg	-3.039	0.021	0.02
female	MNC	Model 2	R_superiorparietal_thickavg	-3.085	0.022	0.02
female	MNC	Model 2	L_insula_surfavg	-3.142	0.023	0.019
female	MNC	Model 3	R_cuneus_surfavg	-3.439	0.039	0.045
female	MNC	Model 4	Lthickness	-3.197	0.075	0.012
female	MNC	Model 4	RThickness	-3.403	0.084	0.009
female	MNC	Model 4	L_lingual_thickavg	-2.492	0.048	0.049
female	MNC	Model 4	L_parsopercularis_thickavg	-2.645	0.053	0.037
female	MNC	Model 4	L_postcentral_thickavg	-4.275	0.128	0.001
female	MNC	Model 4	L_precentral_thickavg	-3.673	0.099	0.006
female	MNC	Model 4	L_precuneus_thickavg	-3.328	0.083	0.01
female	MNC	Model 4	L_superiortemporal_thickavg	-2.564	0.063	0.045
female	MNC	Model 4	L_supramargil_thickavg	-3.042	0.074	0.015
female	MNC	Model 4	R_bankssts_thickavg	-2.866	0.064	0.023
female	MNC	Model 4	R_caudalmiddlefrontal_thickavg	-2.505	0.048	0.049
female	MNC	Model 4	R_lingual_thickavg	-3.387	0.084	0.009
female	MNC	Model 4	R_parsopercularis_thickavg	-3.034	0.07	0.015
female	MNC	Model 4	R_postcentral_thickavg	-4.311	0.129	0.001
female	MNC	Model 4	R_precentral_thickavg	-3.458	0.088	0.009
female	MNC	Model 4	R_precuneus_thickavg	-3.028	0.07	0.015

female	MNC	Model 4	R_supramargil_thickavg	-3.15	0.075	0.013
female	MNC	Model 4	R_transversetemporal_thickavg	-2.754	0.057	0.03
female	MNC	Model 4	R_insula_thickavg	-3.19	0.079	0.012
female	MNC	Model 5	Rpal	-2.661	0.016	0.024
female	MNC	Model 5	Lthickness	-3.66	0.03	0.002
female	MNC	Model 5	RThickness	-3.657	0.03	0.002
female	MNC	Model 5	L_caudalmiddlefrontal_thickavg	-2.485	0.014	0.033
female	MNC	Model 5	L_inferiorparietal_thickavg	-2.912	0.02	0.015
female	MNC	Model 5	L_paracentral_thickavg	-2.653	0.016	0.024
female	MNC	Model 5	L_parsopercularis_thickavg	-3.204	0.024	0.009
female	MNC	Model 5	L_pericalcarine_thickavg	-2.317	0.012	0.05
female	MNC	Model 5	L_postcentral_thickavg	-4.082	0.038	0.001
female	MNC	Model 5	L_precentral_thickavg	-4.243	0.041	0.001
female	MNC	Model 5	L_precuneus_thickavg	-4.25	0.041	0.001
female	MNC	Model 5	L_superiorfrontal_thickavg	-2.886	0.019	0.015
female	MNC	Model 5	L_superiorparietal_thickavg	-2.957	0.02	0.015
female	MNC	Model 5	L_superiortemporal_thickavg	-2.526	0.017	0.031
female	MNC	Model 5	L_supramargil_thickavg	-3.094	0.023	0.012
female	MNC	Model 5	L_transversetemporal_thickavg	-2.585	0.015	0.028
female	MNC	Model 5	R_bankssts_thickavg	-3.587	0.031	0.003
female	MNC	Model 5	R_caudalmiddlefrontal_thickavg	-2.9	0.019	0.015
female	MNC	Model 5	R_cuneus_thickavg	-2.559	0.016	0.029
female	MNC	Model 5	R_inferiorparietal_thickavg	-2.836	0.019	0.015
female	MNC	Model 5	R_lateraloccipital_thickavg	-2.786	0.018	0.017
female	MNC	Model 5	R_lingual_thickavg	-3.571	0.029	0.003
female	MNC	Model 5	R_postcentral_thickavg	-4.441	0.044	0.001
female	MNC	Model 5	R_precentral_thickavg	-3.976	0.036	0.001
female	MNC	Model 5	R_precuneus_thickavg	-3.993	0.037	0.001
female	MNC	Model 5	R_superiorfrontal_thickavg	-2.848	0.019	0.015
female	MNC	Model 5	R_superiorparietal_thickavg	-3.566	0.029	0.003
female	MNC	Model 5	R_superiortemporal_thickavg	-2.929	0.021	0.015
female	MNC	Model 5	R_supramargil_thickavg	-2.838	0.019	0.015
female	MNC	Model 5	R_transversetemporal_thickavg	-2.838	0.018	0.015
female	MNC	Model 5	L_parahippocampal_surfavg	-2.344	0.013	0.048
female	MNC	Model 6	L_precentral_thickavg	-3.343	0.026	0.032
female	MNC	Model 7	Lthickness	-3.064	0.021	0.014
female	MNC	Model 7	RThickness	-3.408	0.026	0.006
female	MNC	Model 7	L_caudalmiddlefrontal_thickavg	-2.44	0.014	0.042
female	MNC	Model 7	L_inferiorparietal_thickavg	-2.821	0.018	0.02
female	MNC	Model 7	L_paracentral_thickavg	-2.52	0.015	0.04
female	MNC	Model 7	L_parsopercularis_thickavg	-3.072	0.022	0.014
female	MNC	Model 7	L_postcentral_thickavg	-4.335	0.043	0.001
female	MNC	Model 7	L_precentral_thickavg	-4.176	0.039	0.001
female	MNC	Model 7	L_precuneus_thickavg	-3.557	0.029	0.005
female	MNC	Model 7	L_superiorparietal_thickavg	-2.781	0.018	0.021
female	MNC	Model 7	L_superiortemporal_thickavg	-2.459	0.016	0.042
female	MNC	Model 7	L_supramargil_thickavg	-3.046	0.022	0.014

female	MNC	Model 7	L_transversetemporal_thickavg	-2.539	0.015	0.039
female	MNC	Model 7	R_bankssts_thickavg	-3.945	0.037	0.001
female	MNC	Model 7	R_caudalmiddlefrontal_thickavg	-2.856	0.019	0.02
female	MNC	Model 7	R_inferiorparietal_thickavg	-2.923	0.02	0.017
female	MNC	Model 7	R_lateraloccipital_thickavg	-2.987	0.021	0.016
female	MNC	Model 7	R_lingual_thickavg	-2.445	0.014	0.042
female	MNC	Model 7	R_middletemporal_thickavg	-2.469	0.014	0.042
female	MNC	Model 7	R_postcentral_thickavg	-4.344	0.043	0.001
female	MNC	Model 7	R_precentral_thickavg	-4.001	0.036	0.001
female	MNC	Model 7	R_precuneus_thickavg	-3.494	0.028	0.005
female	MNC	Model 7	R_superiorfrontal_thickavg	-2.389	0.013	0.047
female	MNC	Model 7	R_superiorparietal_thickavg	-3.195	0.023	0.012
female	MNC	Model 7	R_superiortemporal_thickavg	-2.698	0.018	0.026
female	MNC	Model 7	R_supramargil_thickavg	-2.826	0.019	0.02
female	MNC	Model 7	R_transversetemporal_thickavg	-2.941	0.02	0.017
female	MNC	Model 8	Lthickness	-2.877	0.019	0.026
female	MNC	Model 8	RThickness	-2.906	0.019	0.026
female	MNC	Model 8	L_pericalcarine_thickavg	-3.05	0.021	0.026
female	MNC	Model 8	L_postcentral_thickavg	-3.317	0.025	0.024
female	MNC	Model 8	L_precentral_thickavg	-3.223	0.024	0.025
female	MNC	Model 8	L_precuneus_thickavg	-2.939	0.02	0.026
female	MNC	Model 8	L_superiorfrontal_thickavg	-2.899	0.019	0.026
female	MNC	Model 8	R_postcentral_thickavg	-4.123	0.039	0.003
female	MNC	Model 8	R_precentral_thickavg	-2.923	0.02	0.026
female	MNC	Model 8	R_precuneus_thickavg	-2.874	0.019	0.026
female	MNC	Model 8	R_superiorparietal_thickavg	-2.938	0.02	0.026
female	MNC	Model 9	R_lingual_thickavg	-3.734	0.032	0.015
female	MNC	Model 11	L_precentral_thickavg	-3.657	0.031	0.017
female	MNC	Model 11	L_precuneus_thickavg	-3.239	0.024	0.023
female	MNC	Model 11	R_postcentral_thickavg	-3.031	0.021	0.031
female	MNC	Model 11	R_precentral_thickavg	-3.516	0.028	0.017
female	MNC	Model 11	R_precuneus_thickavg	-3.315	0.026	0.023
female	MNC	Model 12	Rpal	-3.233	0.042	0.019
female	MNC	Model 12	RThickness	-2.564	0.027	0.049
female	MNC	Model 12	L_parsorbitalis_thickavg	-2.583	0.028	0.049
female	MNC	Model 12	L_postcentral_thickavg	-2.979	0.037	0.028
female	MNC	Model 12	L_precentral_thickavg	-3.129	0.04	0.02
female	MNC	Model 12	L_precuneus_thickavg	-3.678	0.055	0.006
female	MNC	Model 12	L_superiortemporal_thickavg	-2.587	0.031	0.049
female	MNC	Model 12	L_transversetemporal_thickavg	-2.732	0.03	0.038
female	MNC	Model 12	R_cuneus_thickavg	-2.547	0.027	0.049
female	MNC	Model 12	R_lateraloccipital_thickavg	-2.843	0.033	0.029
female	MNC	Model 12	R_postcentral_thickavg	-3.87	0.059	0.006
female	MNC	Model 12	R_precentral_thickavg	-3.123	0.04	0.02
female	MNC	Model 12	R_precuneus_thickavg	-3.229	0.044	0.019
female	MNC	Model 12	R_superiorparietal_thickavg	-3.747	0.056	0.006
female	MNC	Model 12	R_supramargil_thickavg	-2.87	0.035	0.029

female	MNC	Model 12	R_cuneus_surfavg	-2.848	0.034	0.029
female	MNC	Model 12	global gray matter density	-2.94	0.029	0.028
female	MNC	Model 13	Rcaud	-2.74	0.03	0.043
female	MNC	Model 13	Rpal	-3.446	0.048	0.011
female	MNC	Model 13	RThickness	-10.417	0.311	0
female	MNC	Model 13	L_precentral_thickavg	-2.872	0.034	0.039
female	MNC	Model 13	L_precuneus_thickavg	-3.092	0.039	0.026
female	MNC	Model 13	R_cuneus_thickavg	-3.183	0.042	0.023
female	MNC	Model 13	R_lingual_thickavg	-2.818	0.032	0.042
female	MNC	Model 13	R_postcentral_thickavg	-3.688	0.054	0.009
female	MNC	Model 13	R_superiorparietal_thickavg	-3.526	0.05	0.011
female	MNC	Model 13	L_insula_surfavg	-2.905	0.035	0.039
female	MNC	Model 13	R_entorhil_surfavg	-2.77	0.039	0.043
female	MNC	Model 14	R_cuneus_surfavg	-3.68	0.078	0.018
female	MNC	Model 15	Lthickness	-2.996	0.117	0.026
female	MNC	Model 15	RThickness	-2.891	0.109	0.026
female	MNC	Model 15	L_parsopercularis_thickavg	-2.88	0.112	0.026
female	MNC	Model 15	L_parstriangularis_thickavg	-2.939	0.116	0.026
female	MNC	Model 15	L_postcentral_thickavg	-3.359	0.146	0.02
female	MNC	Model 15	L_precentral_thickavg	-2.95	0.116	0.026
female	MNC	Model 15	L_precuneus_thickavg	-3.286	0.141	0.02
female	MNC	Model 15	L_superiortemporal_thickavg	-3.645	0.2	0.01
female	MNC	Model 15	R_lingual_thickavg	-3.149	0.129	0.025
female	MNC	Model 15	R_postcentral_thickavg	-4	0.19	0.005
female	MNC	Model 15	R_supramargil_thickavg	-3.052	0.122	0.026
female	MNC	Model 15	R_transversetemporal_thickavg	-2.934	0.112	0.026
female	BiDirect	Model 7	L_lingual_thickavg	-3.55	0.046	0.03
female	BiDirect	Model 7	L_precuneus_thickavg	-3.248	0.039	0.04
female	BiDirect	Model 7	R_parsorbitalis_thickavg	-3.163	0.037	0.04
male	Pooled	Model 2	L_bankssts_thickavg	-3.438	0.012	0.045
male	MACS	Model 17	R_bankssts_surfavg	-3.616	0.048	0.046
male	MNC	Model 9	Lthal	-4.126	0.046	0.003
male	MNC	Model 9	Rthal	-3.882	0.04	0.004
male	MNC	Model 9	L_precuneus_surfavg	-3.025	0.025	0.036
male	MNC	Model 9	L_rostralmiddlefrontal_surfavg	-2.945	0.024	0.036
male	MNC	Model 9	R_bankssts_surfavg	-2.952	0.026	0.036
male	MNC	Model 9	R_rostralmiddlefrontal_surfavg	-3.028	0.025	0.036
male	MNC	Model 16	R_superiorparietal_thickavg	-3.699	0.578	0.008
male	MNC	Model 16	L_inferiorparietal_surfavg	-4.87	0.772	0
male	BiDirect	Model 16	Lcaud	-3.914	0.605	0.007

Note. All regions significant at p-FDR<.05 are shown. All Freesurfer-derived variables and voxel-wise derived global gray matter density are included.

Table S20

Replicability across cohorts indicated by spatial overlap in significance at a level of $p_{FDR}<.05$ for parcellation-based Freesurfer and global gray matter density analysis

		MACS-MNC		MACS-BiDirect		MNC-BiDirect		MACS-MNC-BiDirect	
Sex-stratification	Model	k-overlap	regions	k-overlap	regions	k-overlap	regions	k-overlap	regions
-	Model 1	0	-	0	-	0	-	0	-
-	Model 2	0	-	0	-	0	-	0	-
-	Model 3	0	-	0	-	0	-	0	-
-	Model 4	0	-	0	-	0	-	0	-
-	Model 5	0	-	0	-	0	-	0	-
-	Model 6	0	-	0	-	0	-	0	-
-	Model 7	0	-	0	-	0	-	0	-
-	Model 8	0	-	0	-	0	-	0	-
-	Model 9	0	-	0	-	0	-	0	-
-	Model 10	0	-	0	-	0	-	0	-
-	Model 11	0	-	0	-	0	-	0	-
-	Model 12	0	-	0	-	0	-	0	-
-	Model 13	0	-	0	-	0	-	0	-
-	Model 14	0	-	0	-	0	-	0	-
-	Model 15	0	-	0	-	0	-	0	-
-	Model 16	0	-	0	-	0	-	0	-
-	Model 17	0	-	0	-	0	-	0	-
-	Model 18	0	-	0	-	0	-	0	-
female	Model 1	0	-	0	-	0	-	0	-

female	Model 2	0	-	0	-	0	-	0	-
female	Model 3	0	-	0	-	0	-	0	-
female	Model 4	0	-	0	-	0	-	0	-
female	Model 5	0	-	0	-	0	-	0	-
female	Model 6	0	-	0	-	0	-	0	-
female	Model 7	0	-	0	-	1	L_precuneus_thickavg	0	-
female	Model 8	0	-	0	-	0	-	0	-
female	Model 9	0	-	0	-	0	-	0	-
female	Model 10	0	-	0	-	0	-	0	-
female	Model 11	0	-	0	-	0	-	0	-
female	Model 12	0	-	0	-	0	-	0	-
female	Model 13	0	-	0	-	0	-	0	-
female	Model 14	0	-	0	-	0	-	0	-
female	Model 15	0	-	0	-	0	-	0	-
female	Model 16	0	-	0	-	0	-	0	-
female	Model 17	0	-	0	-	0	-	0	-
female	Model 18	0	-	0	-	0	-	0	-
male	Model 1	0	-	0	-	0	-	0	-
male	Model 2	0	-	0	-	0	-	0	-
male	Model 3	0	-	0	-	0	-	0	-
male	Model 4	0	-	0	-	0	-	0	-
male	Model 5	0	-	0	-	0	-	0	-
male	Model 6	0	-	0	-	0	-	0	-
male	Model 7	0	-	0	-	0	-	0	-

male	Model 8	0	-	0	-	0	-	0	-
male	Model 9	0	-	0	-	0	-	0	-
male	Model 10	0	-	0	-	0	-	0	-
male	Model 11	0	-	0	-	0	-	0	-
male	Model 12	0	-	0	-	0	-	0	-
male	Model 13	0	-	0	-	0	-	0	-
male	Model 14	0	-	0	-	0	-	0	-
male	Model 15	0	-	0	-	0	-	0	-
male	Model 16	0	-	0	-	0	-	0	-
male	Model 17	0	-	0	-	0	-	0	-
male	Model 18	0	-	0	-	0	-	0	-

Note. Number of regions showing spatial overlap in significance ($p_{FDR}<.05$) across cohorts are shown. All Freesurfer-derived variables and voxel-wise derived global gray matter density are included.

Figure S1

Age distribution across cohorts and diagnosis groups

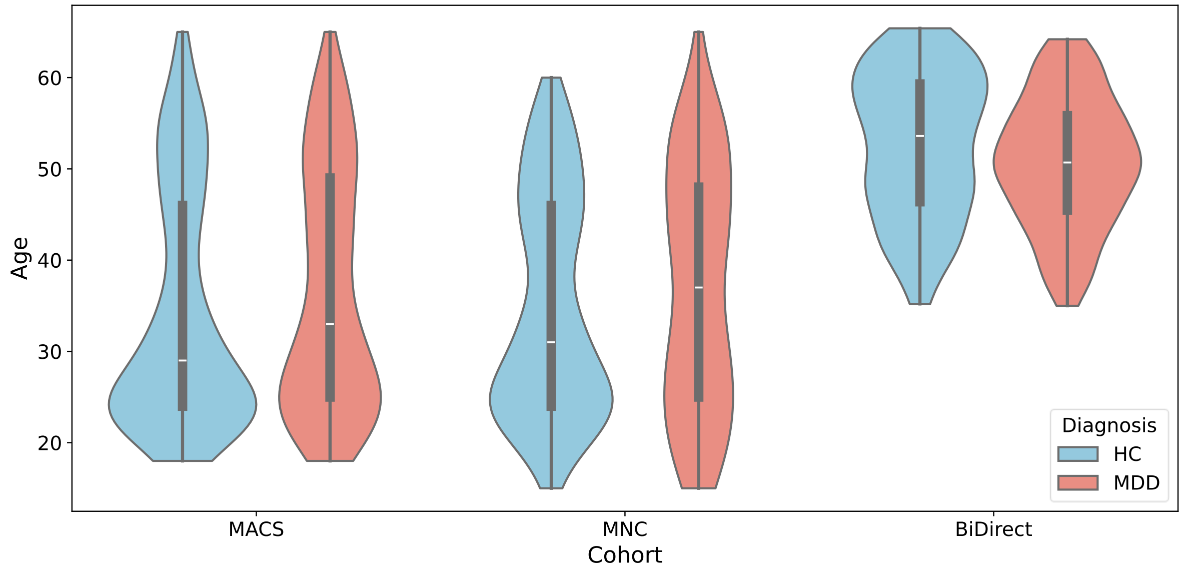

Note. Violin plots are shown depicting the distribution of age across cohorts and diagnosis groups. Boxplots within the violins depict the median (center line), the interquartile range (IQR; box from the 25th to 75th percentile), and whiskers extending to the most extreme values within 1.5 × IQR. HC, healthy controls; MDD, major depressive disorder.

Figure S2

Association between CTQ scales, demographic variables and clinical variables within the ***MACS*** *cohort*

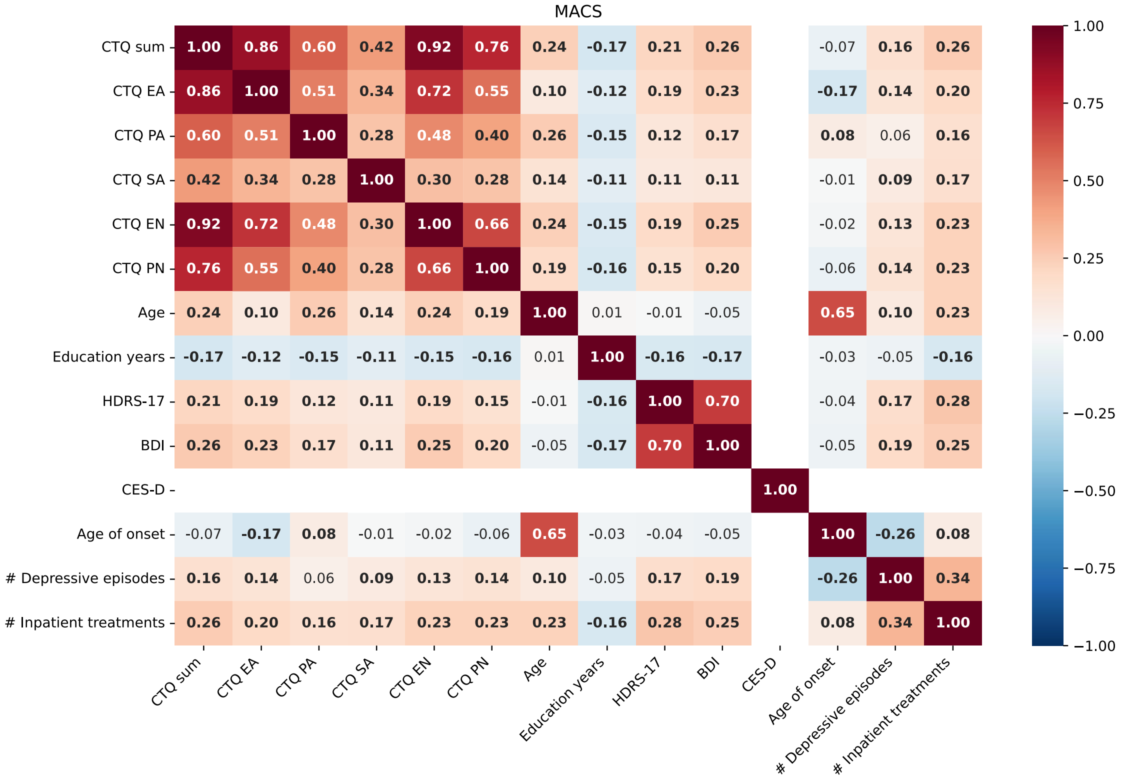

Note. Spearman correlations are shown. All correlations involving clinical variables (HDRS-17, BDI, Age of onset, number of depressive episodes and number of inpatient treatments) were only calculated within the MDD subsample. The BDI was only available within MACS and MNC, while the CES-D was only available for the BiDirect cohort. Significant associations at p<.05 are shown in bold font. CTQ, childhood trauma questionnaire; EA, emotional abuse; PA, physical abuse; SA, sexual abuse; EN, emotional neglect; PN, physical neglect; HDRS-17, 17-item Hamilton Depression Rating Scale; BDI, Beck Depression Inventory; CES-D, Center for Epidemiologic Studies Depression Scale.

Figure S3

*Association between CTQ scales, demographic variables and clinical variables within the **MNC** cohort*

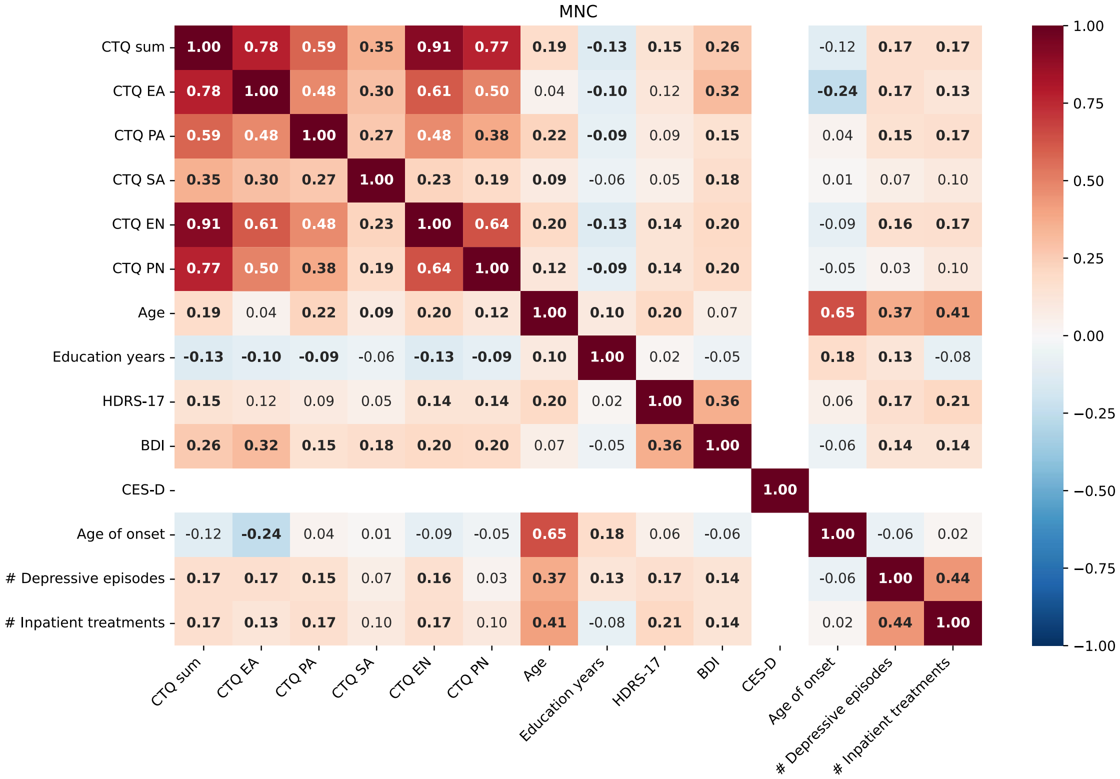

Note. Spearman correlations are shown. All correlations involving clinical variables (HDRS-17, BDI, Age of onset, number of depressive episodes and number of inpatient treatments) were only calculated within the MDD subsample. The BDI was only available within MACS and MNC, while the CES-D was only available for the BiDirect cohort. Significant associations at $p<.05$ are shown in bold font. CTQ, childhood trauma questionnaire; EA, emotional abuse; PA, physical abuse; SA, sexual abuse; EN, emotional neglect; PN, physical neglect; HDRS-17, 17-item Hamilton Depression Rating Scale; BDI, Beck Depression Inventory; CES-D, Center for Epidemiologic Studies Depression Scale.

Figure S4

Association between CTQ scales, demographic variables and clinical variables within the ***BiDirect*** *cohort*

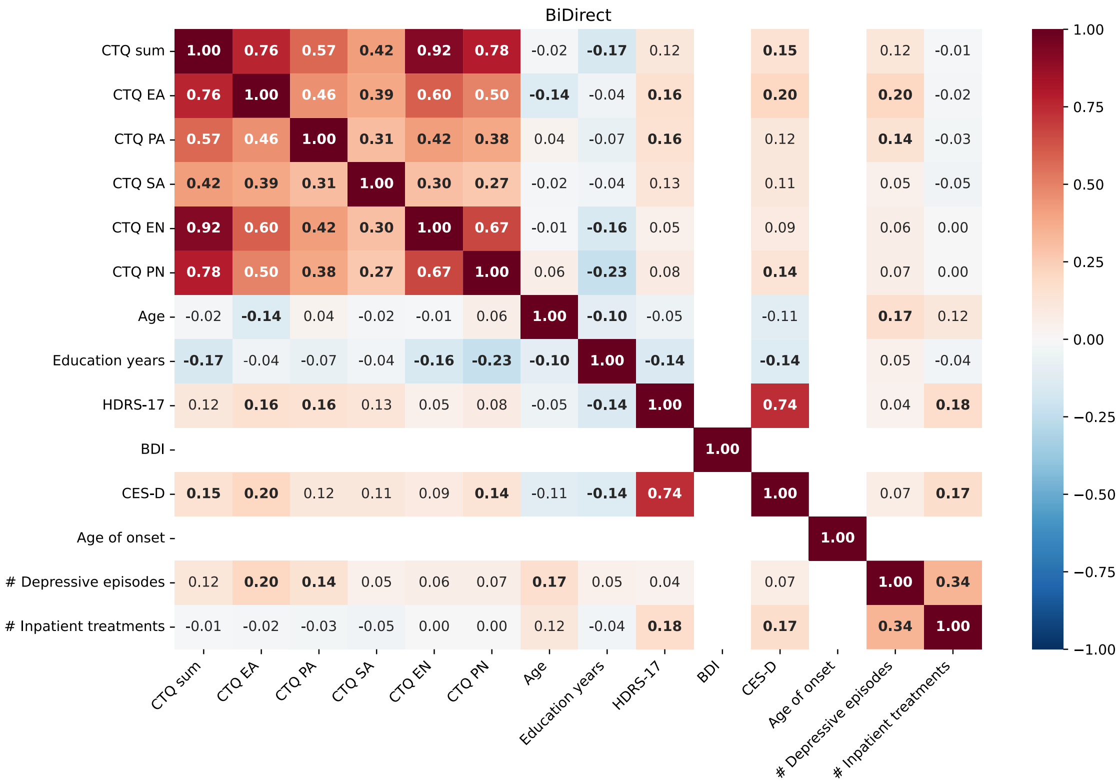

Note. Spearman correlations are shown. All correlations involving clinical variables (HDRS-17, BDI, Age of onset, number of depressive episodes and number of inpatient treatments) were only calculated within the MDD subsample. The BDI was only available within MACS and MNC, while the CES-D was only available for the BiDirect cohort. Significant associations at p<.05 are shown in bold font. CTQ, childhood trauma questionnaire; EA, emotional abuse; PA, physical abuse; SA, sexual abuse; EN, emotional neglect; PN, physical neglect; HDRS-17, 17-item Hamilton Depression Rating Scale; BDI, Beck Depression Inventory; CES-D, Center for Epidemiologic Studies Depression Scale.

Figure S5

Distributions of CTQ scales across HC and MDD groups stratified by cohorts

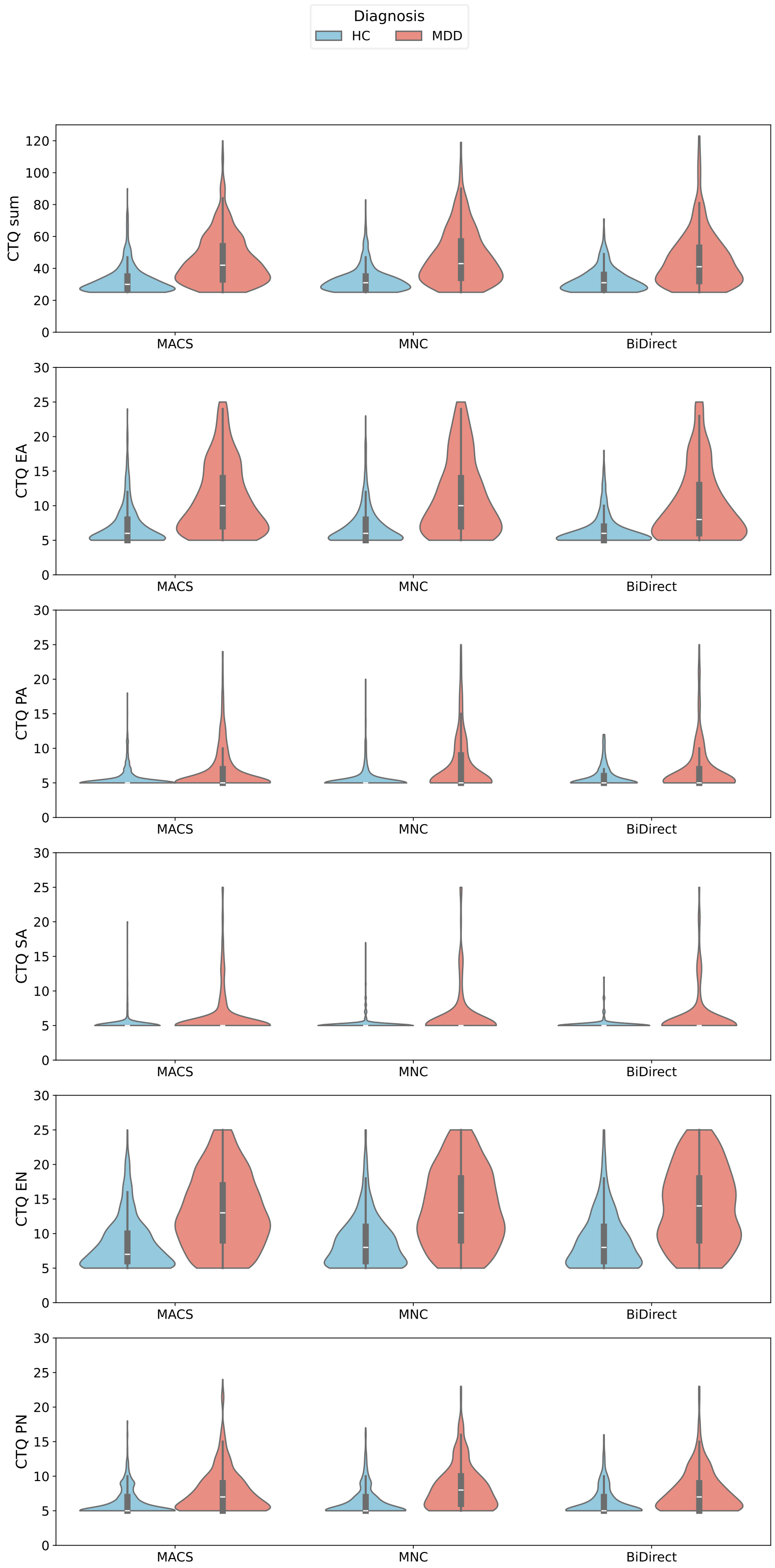

Note. Violin plots are shown depicting the distribution of the CTQ sum scale, as well as the five CTQ subscales. Boxplots within the violins depict the median (center line), the interquartile range (IQR; box from the 25th to 75th percentile), and whiskers extending to the most extreme values within 1.5 × IQR. HC, healthy controls; MDD, major depressive disorder; CTQ, childhood trauma questionnaire; EA, emotional abuse; PA, physical abuse; SA, sexual abuse; EN, emotional neglect; PN, physical neglect.

Figure S6

Significant clusters across cohort-wise analyses (replicability analysis) for ***model 1*** *– CTQ sum in HC and MDD samples controlling for MDD diagnosis*

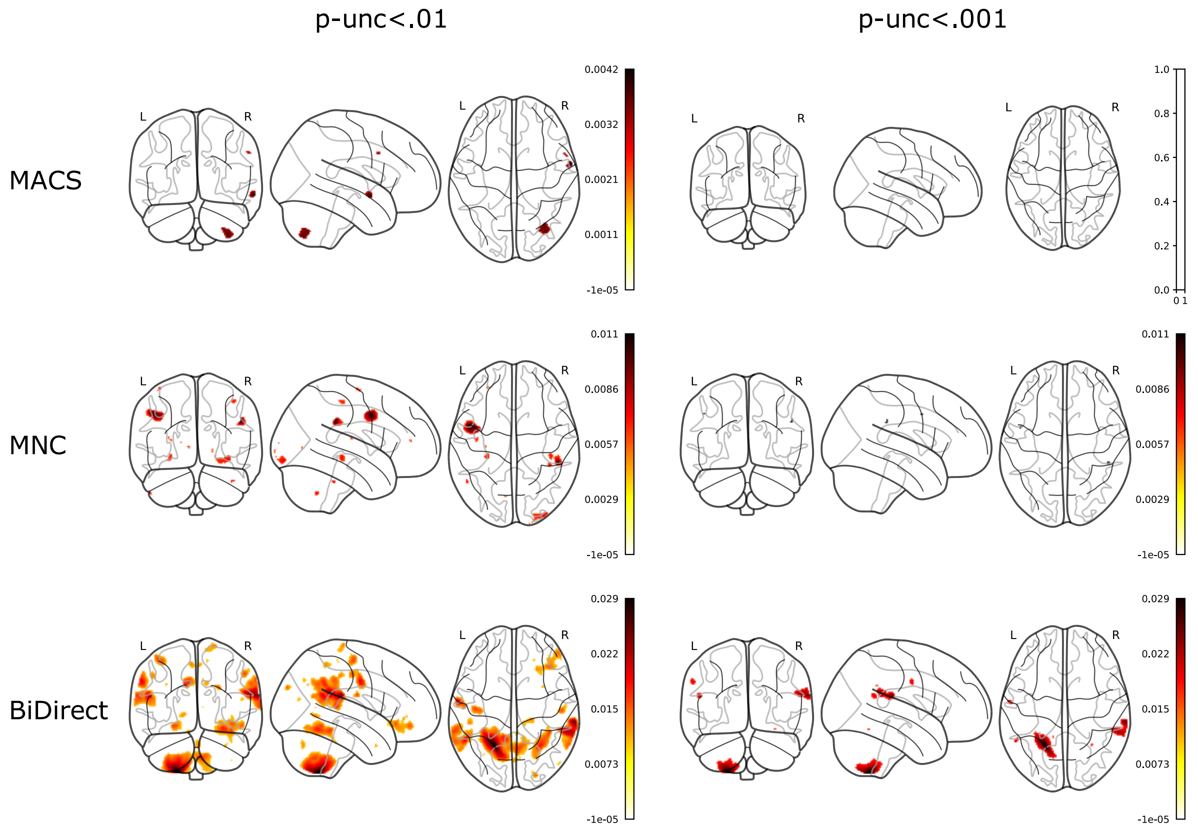

Note. Glass brains are shown with maximum intensity projections. Color bars represent the partial R^2 of the maltreatment predictor. Empty color bars indicate that no voxels survived the corresponding significance threshold. Results are shown thresholded using the uncorrected thresholds $p_{unc}<.01$ (left column) and $p_{unc}<.001$ (right column).

Figure S7

*Significant clusters across cohort-wise analyses (replicability analysis) for **model 2** – CTQ sum as a predictor in HC and MDD samples without controlling for MDD diagnosis*

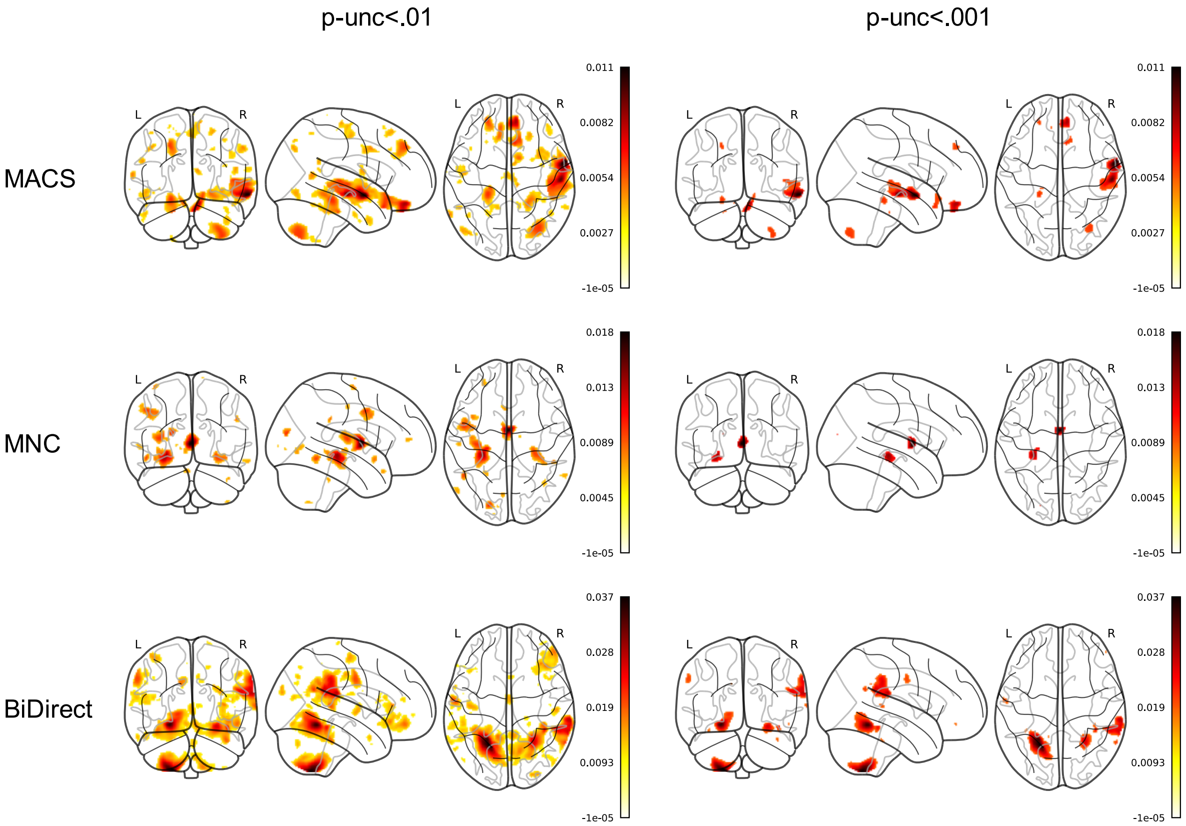

Note. Glass brains are shown with maximum intensity projections. Color bars represent the partial R^2 of the maltreatment predictor. Empty color bars indicate that no voxels survived the corresponding significance threshold. Results are shown thresholded using the uncorrected thresholds $p_{unc}<.01$ (left column) and $p_{unc}<.001$ (right column).

Figure S8

*Significant clusters across cohort-wise analyses (replicability analysis) for **model 3** – CTQ sum as a predictor in HC subsamples*

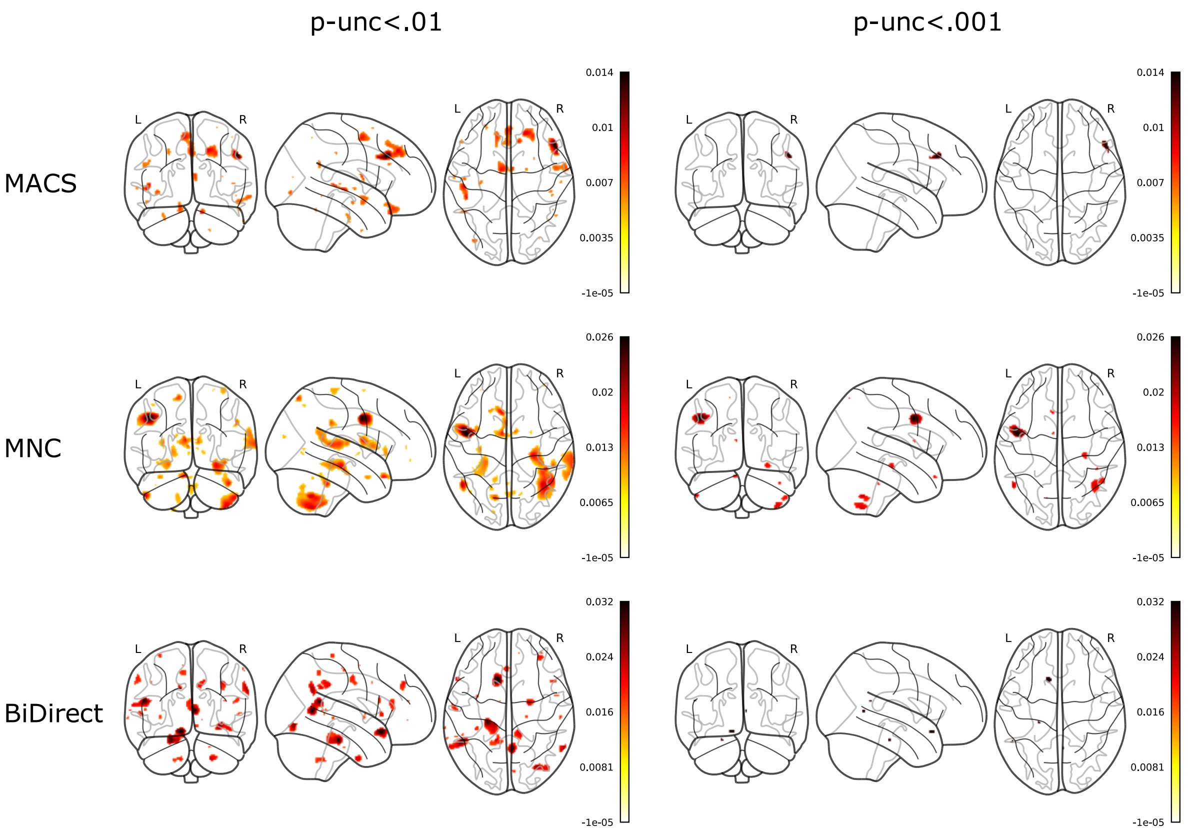

Note. Glass brains are shown with maximum intensity projections. Color bars represent the partial R^2 of the maltreatment predictor. Empty color bars indicate that no voxels survived the corresponding significance threshold. Results are shown thresholded using the uncorrected thresholds $p_{unc}<.01$ (left column) and $p_{unc}<.001$ (right column).

Figure S9

Significant clusters across cohort-wise analyses (replicability analysis) for ***model 4*** *– CTQ sum as a predictor in MDD subsamples*

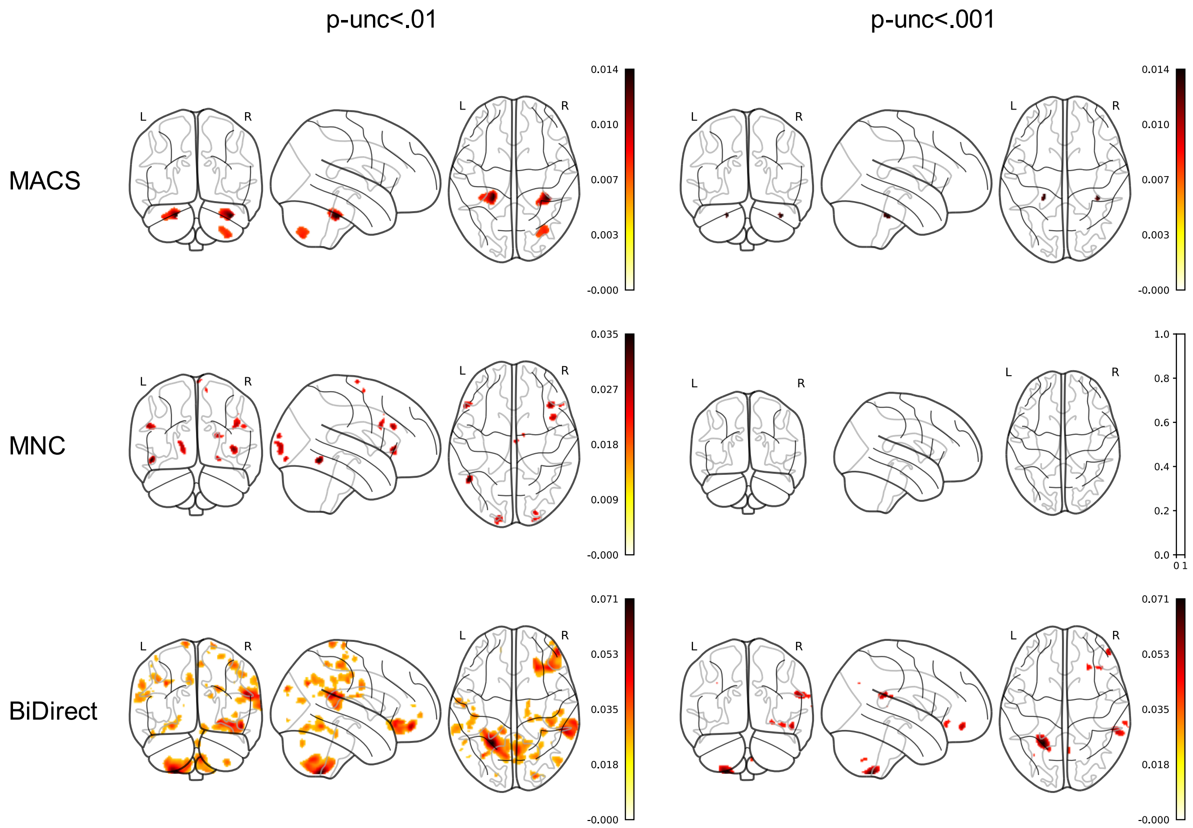

Note. Glass brains are shown with maximum intensity projections. Color bars represent the partial R^2 of the maltreatment predictor. Empty color bars indicate that no voxels survived the corresponding significance threshold. Results are shown thresholded using the uncorrected thresholds p_{unc}<.01 (left column) and p_{unc}<.001 (right column).

Figure S10

Significant clusters across cohort-wise analyses (replicability analysis) for ***model 5*** *– CTQ abuse subscales as a predictor in HC and MDD samples controlling for MDD diagnosis*

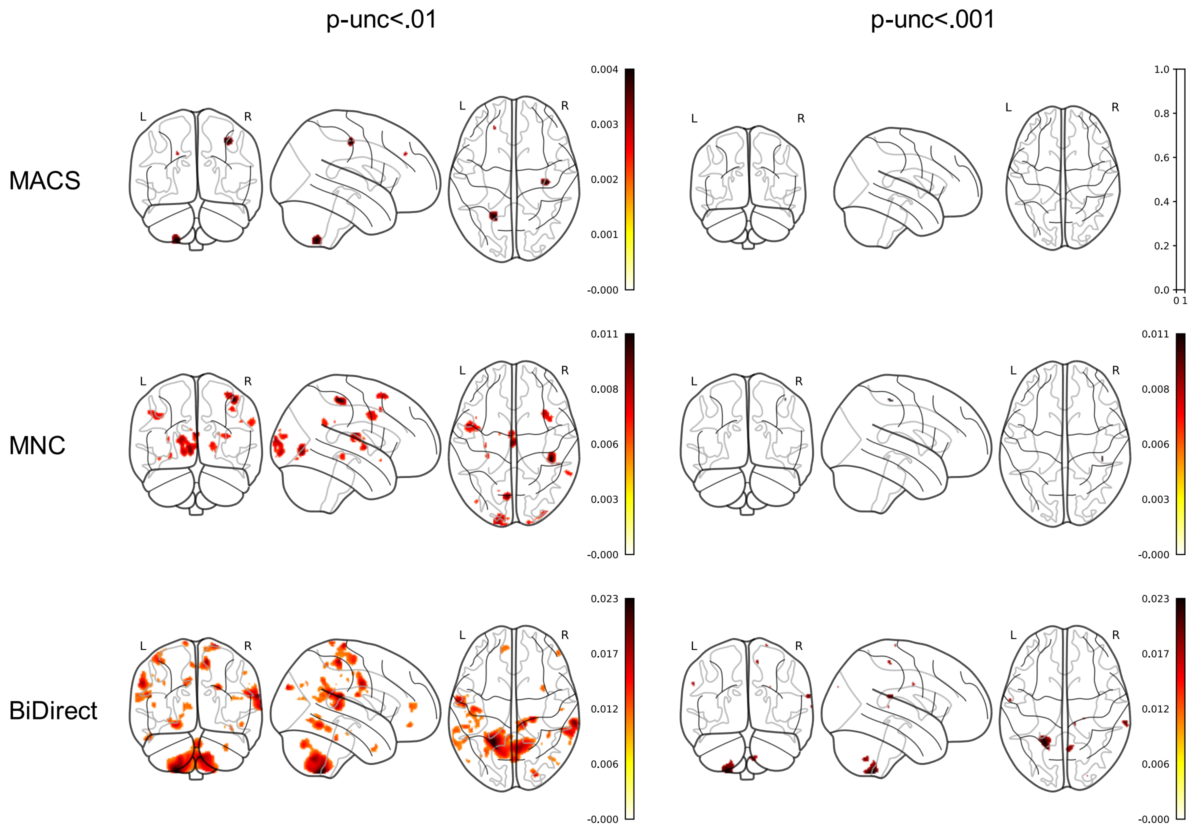

Note. Glass brains are shown with maximum intensity projections. Color bars represent the partial R^2 of the maltreatment predictor. Empty color bars indicate that no voxels survived the corresponding significance threshold. Results are shown thresholded using the uncorrected thresholds $p_{unc}<.01$ (left column) and $p_{unc}<.001$ (right column).

Figure S11

Significant clusters across cohort-wise analyses (replicability analysis) for ***model 6*** *– CTQ neglect subscales as a predictor in HC and MDD samples controlling for MDD diagnosis*

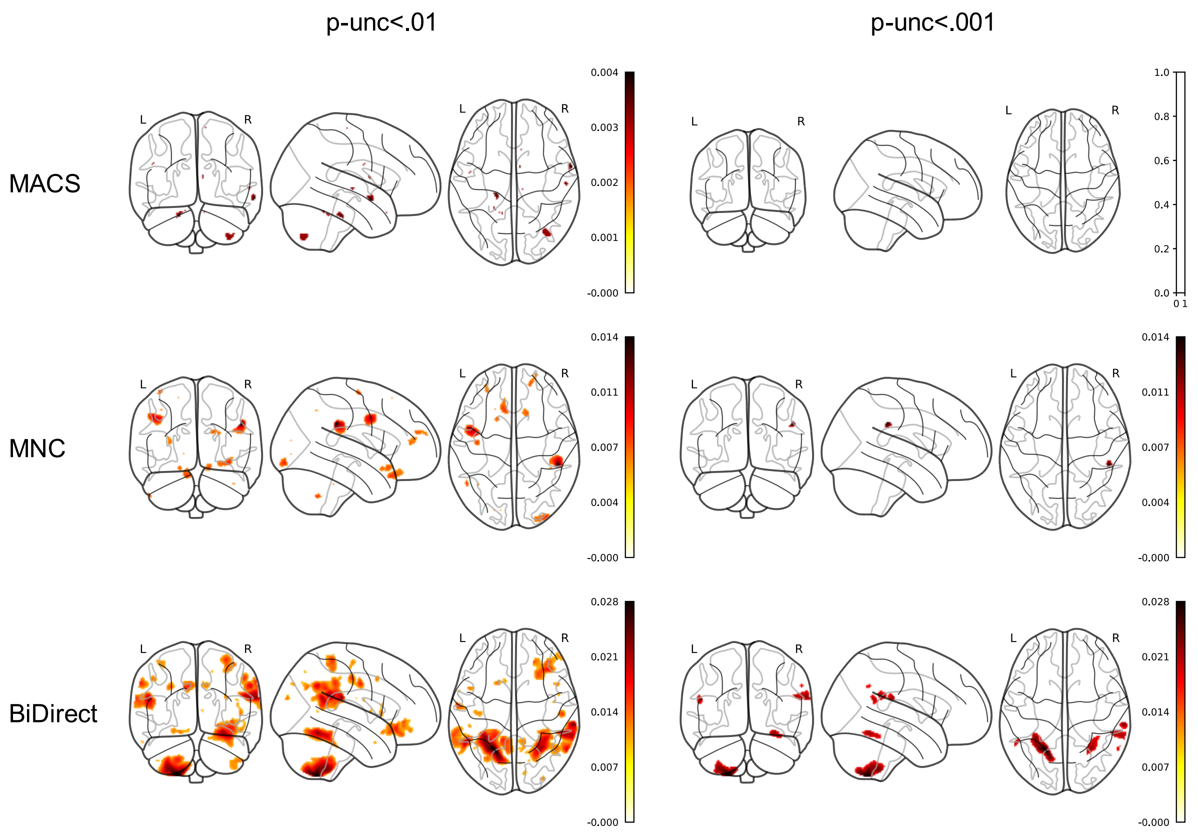

Note. Glass brains are shown with maximum intensity projections. Color bars represent the partial R^2 of the maltreatment predictor. Empty color bars indicate that no voxels survived the corresponding significance threshold. Results are shown thresholded using the uncorrected thresholds $p_{unc}<.01$ (left column) and $p_{unc}<.001$ (right column).

Figure S12

Significant clusters across cohort-wise analyses (replicability analysis) for ***model 7*** *– CTQ subscale emotional abuse as a predictor in HC and MDD samples controlling for MDD diagnosis*

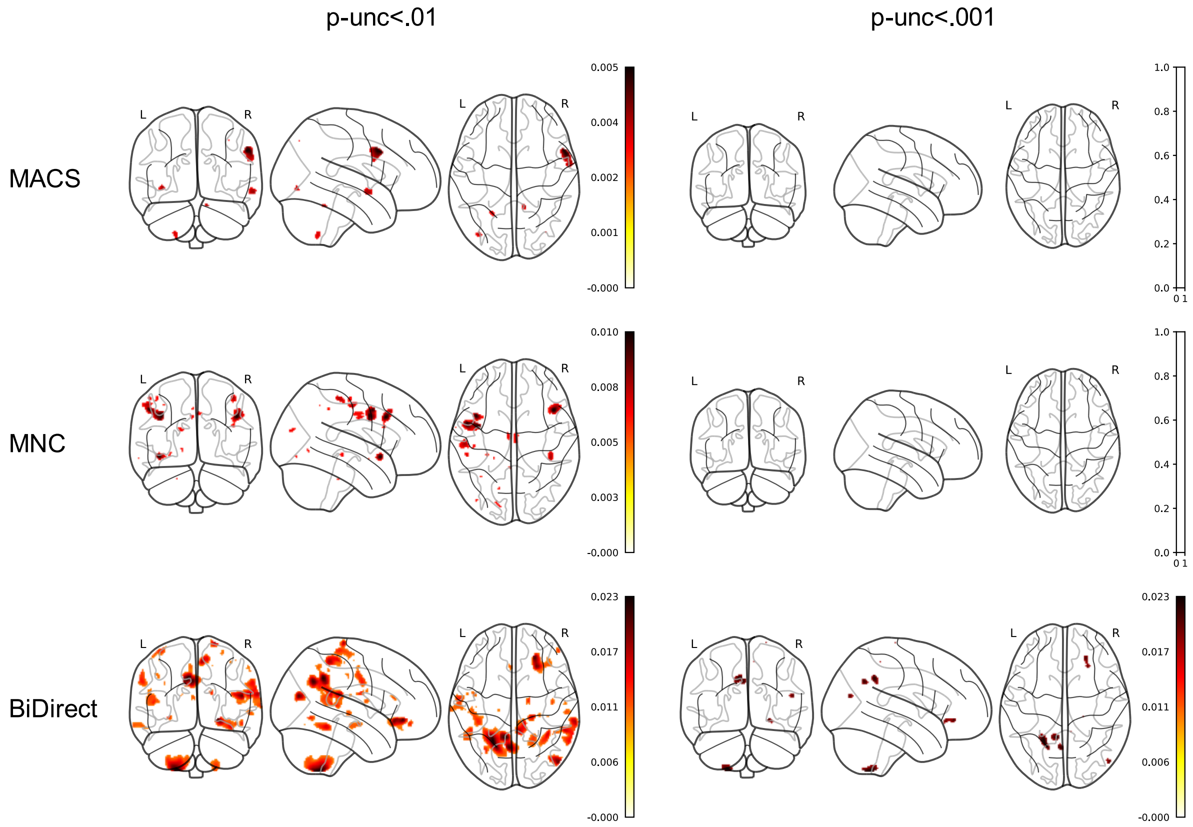

Note. Glass brains are shown with maximum intensity projections. Color bars represent the partial R^2 of the maltreatment predictor. Empty color bars indicate that no voxels survived the corresponding significance threshold. Results are shown thresholded using the uncorrected thresholds $p_{unc}<.01$ (left column) and $p_{unc}<.001$ (right column).

Figure S13

*Significant clusters across cohort-wise analyses (replicability analysis) for **model 8** – CTQ subscale physical abuse as a predictor in HC and MDD samples controlling for MDD diagnosis*

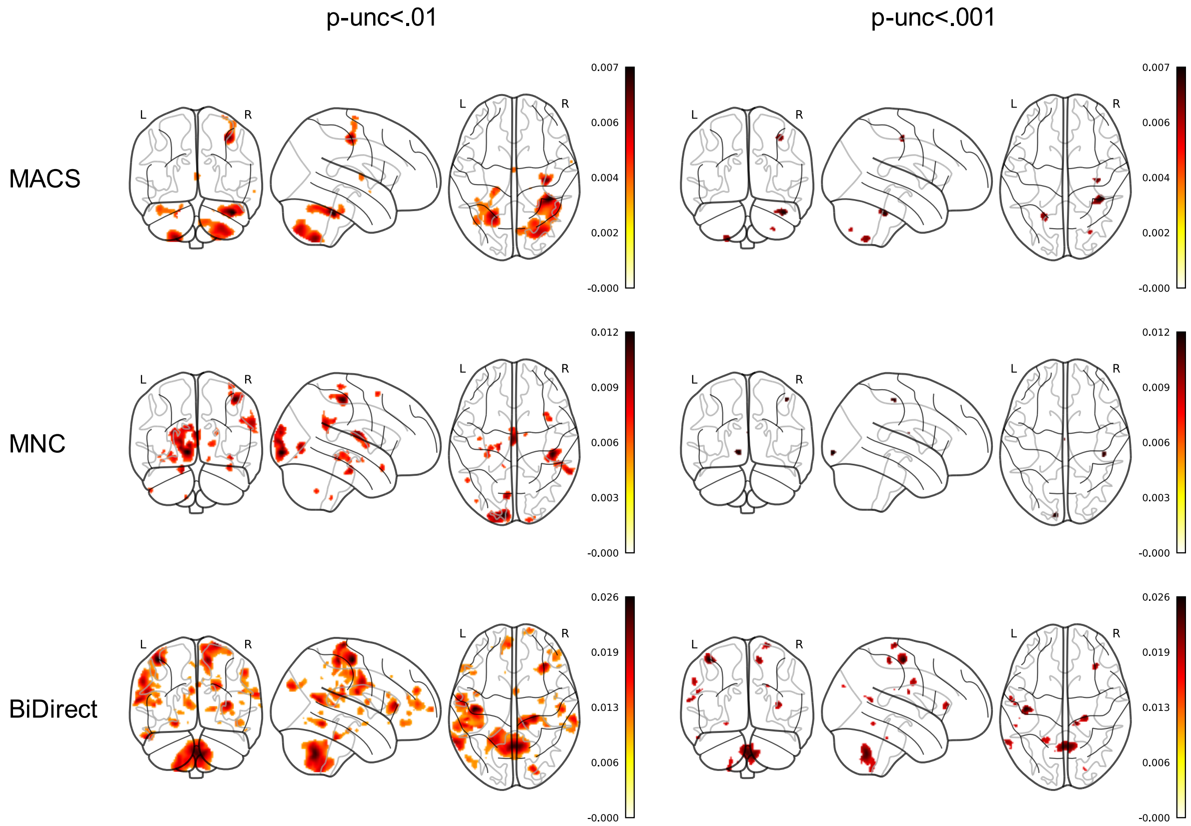

Note. Glass brains are shown with maximum intensity projections. Color bars represent the partial R^2 of the maltreatment predictor. Empty color bars indicate that no voxels survived the corresponding significance threshold. Results are shown thresholded using the uncorrected thresholds p_{unc}<.01 (left column) and p_{unc}<.001 (right column).

Figure S14

Significant clusters across cohort-wise analyses (replicability analysis) for ***model 9*** *– CTQ subscale sexual abuse as a predictor in HC and MDD samples controlling for MDD diagnosis*

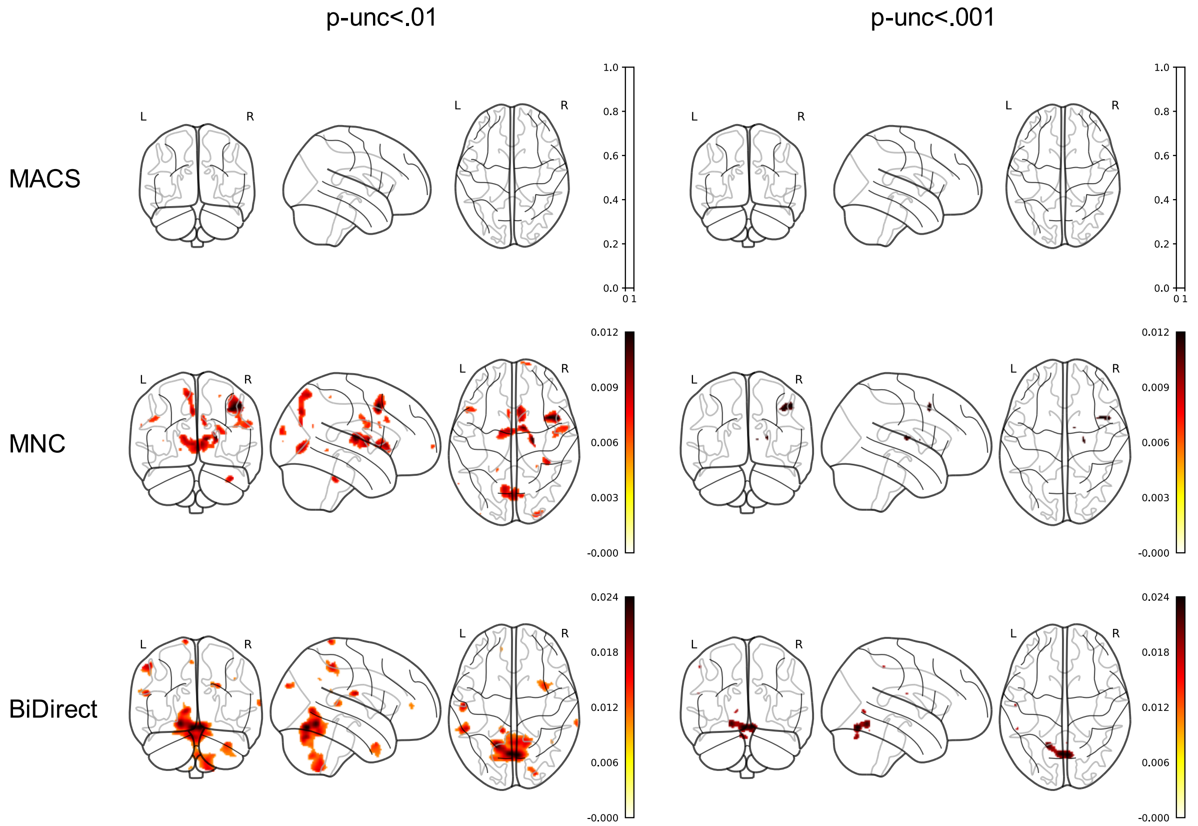

Note. Glass brains are shown with maximum intensity projections. Color bars represent the partial R^2 of the maltreatment predictor. Empty color bars indicate that no voxels survived the corresponding significance threshold. Results are shown thresholded using the uncorrected thresholds $p_{unc}<.01$ (left column) and $p_{unc}<.001$ (right column).

Figure S15

*Significant clusters across cohort-wise analyses (replicability analysis) for **model 10** – CTQ subscale emotional neglect as a predictor in HC and MDD samples controlling for MDD diagnosis*

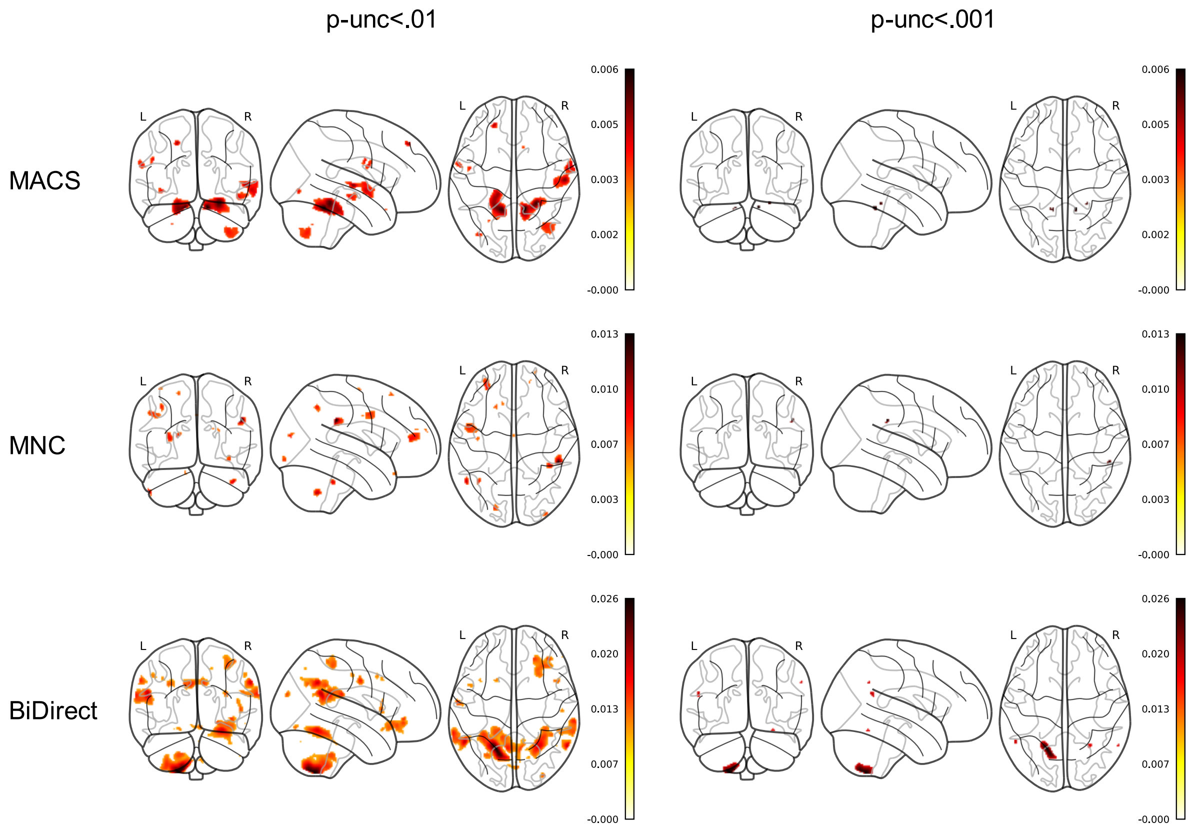

Note. Glass brains are shown with maximum intensity projections. Color bars represent the partial R^2 of the maltreatment predictor. Empty color bars indicate that no voxels survived the corresponding significance threshold. Results are shown thresholded using the uncorrected thresholds $p_{unc}<.01$ (left column) and $p_{unc}<.001$ (right column).

Figure S16

*Significant clusters across cohort-wise analyses (replicability analysis) for **model 11** – CTQ subscale physical neglect as a predictor in HC and MDD samples controlling for MDD diagnosis*

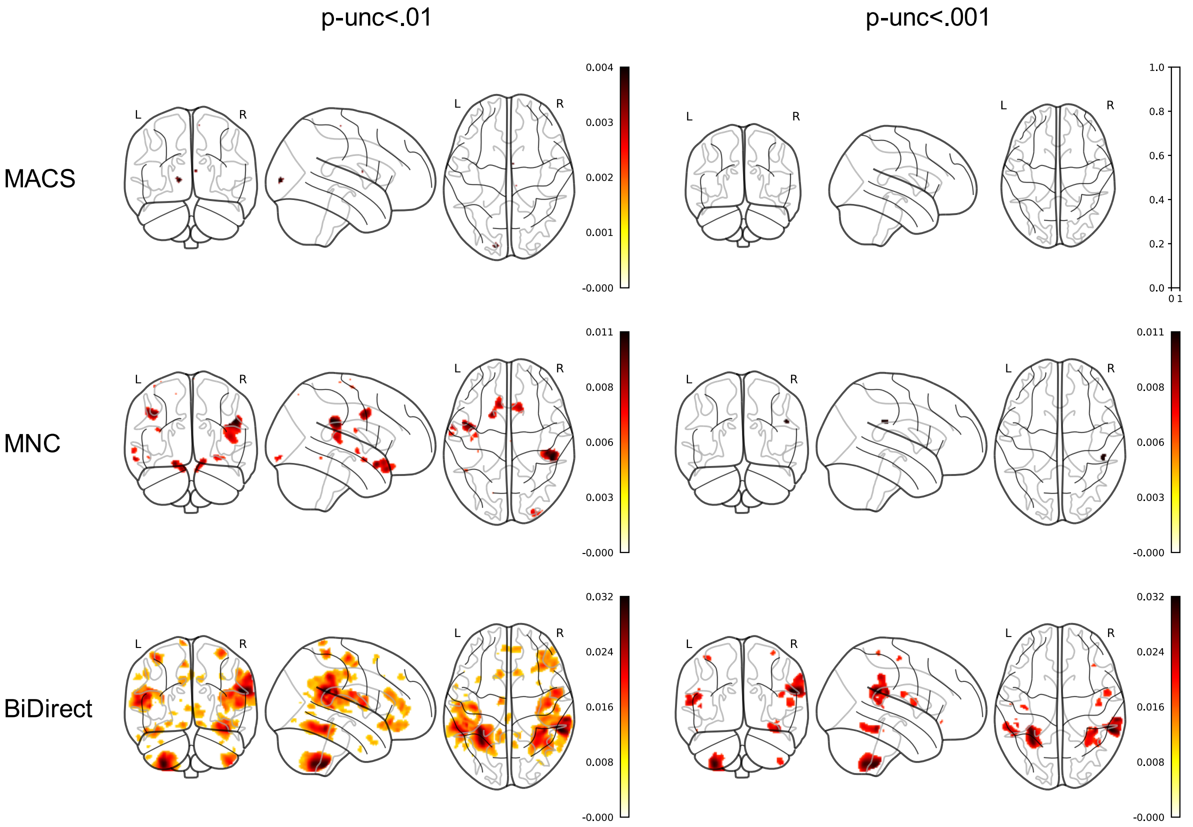

Note. Glass brains are shown with maximum intensity projections. Color bars represent the partial R^2 of the maltreatment predictor. Empty color bars indicate that no voxels survived the corresponding significance threshold. Results are shown thresholded using the uncorrected thresholds $p_{unc}<.01$ (left column) and $p_{unc}<.001$ (right column).

Figure S17

Significant clusters across cohort-wise analyses (replicability analysis) for ***model 12*** *– CTQ extreme severity compared to none in HC and MDD samples controlling for MDD diagnosis*

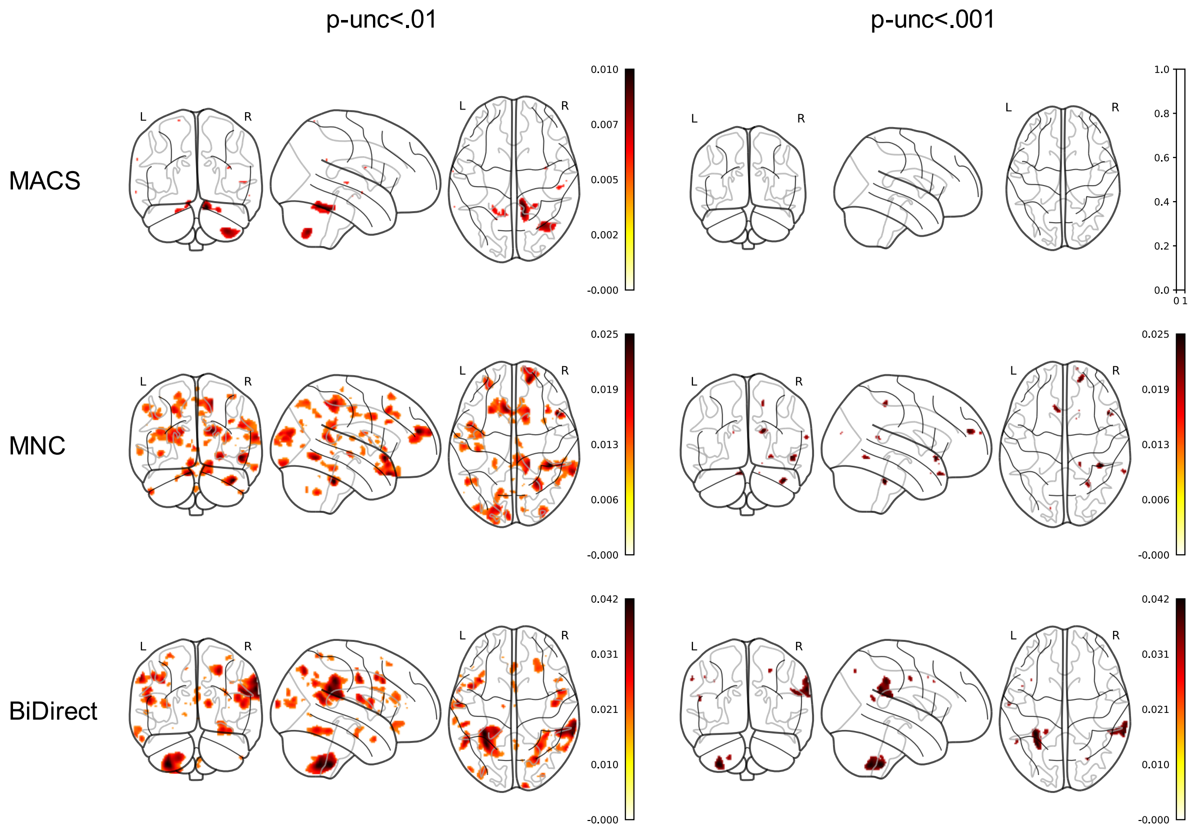

Note. Glass brains are shown with maximum intensity projections. Color bars represent the partial R^2 of the maltreatment predictor. Empty color bars indicate that no voxels survived the corresponding significance threshold. Results are shown thresholded using the uncorrected thresholds $p_{unc}<.01$ (left column) and $p_{unc}<.001$ (right column).

Figure S18

Significant clusters across cohort-wise analyses (replicability analysis) for ***model 13*** *– CTQ extreme severity compared to none in HC and MDD samples without controlling for MDD diagnosis*

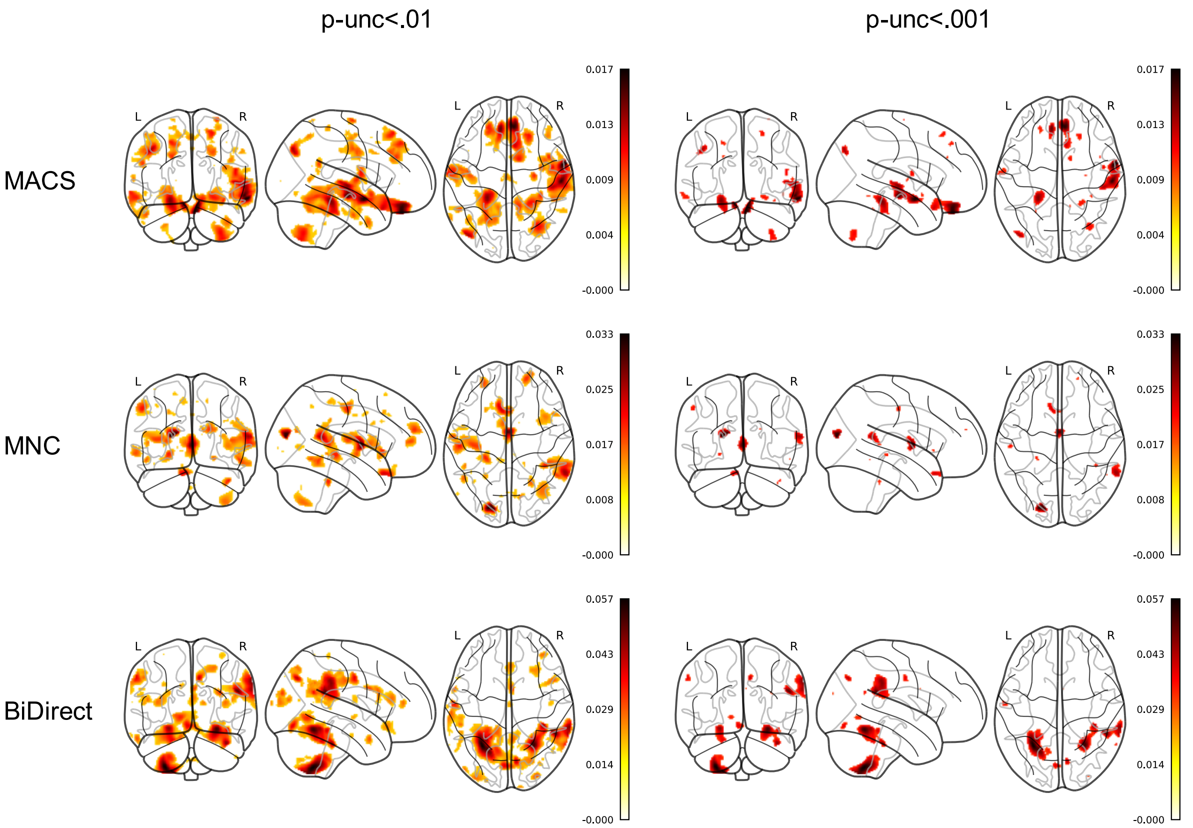

Note. Glass brains are shown with maximum intensity projections. Color bars represent the partial R^2 of the maltreatment predictor. Empty color bars indicate that no voxels survived the corresponding significance threshold. Results are shown thresholded using the uncorrected thresholds $p_{unc}<.01$ (left column) and $p_{unc}<.001$ (right column).

Figure S19

Significant clusters across cohort-wise analyses (replicability analysis) for ***model 14*** *– CTQ extreme severity compared to none in HC subsamples*

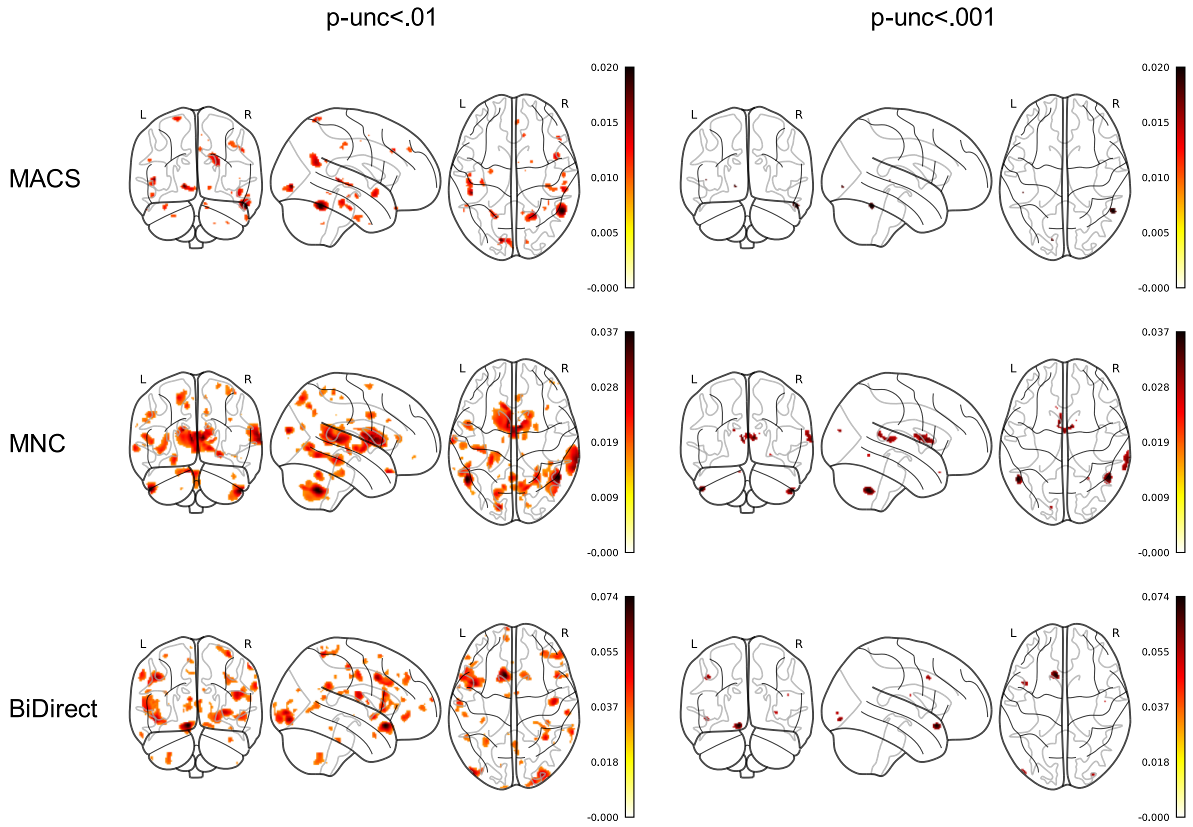

Note. Glass brains are shown with maximum intensity projections. Color bars represent the partial R^2 of the maltreatment predictor. Empty color bars indicate that no voxels survived the corresponding significance threshold. Results are shown thresholded using the uncorrected thresholds $p_{unc}<.01$ (left column) and $p_{unc}<.001$ (right column).

Figure S20

Significant clusters across cohort-wise analyses (replicability analysis) for ***model 15*** *– CTQ extreme severity compared to none in MDD subsamples*

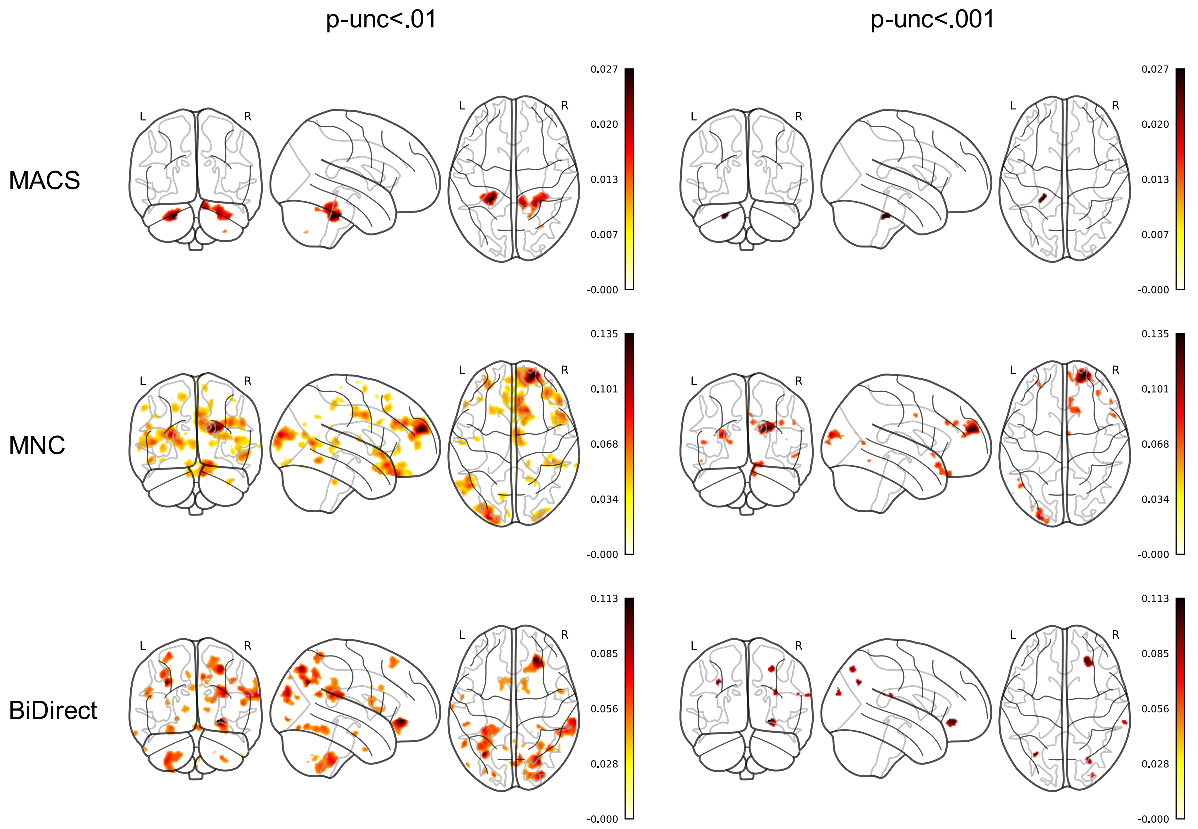

Note. Glass brains are shown with maximum intensity projections. Color bars represent the partial R^2 of the maltreatment predictor. Empty color bars indicate that no voxels survived the corresponding significance threshold. Results are shown thresholded using the uncorrected thresholds $p_{unc}<.01$ (left column) and $p_{unc}<.001$ (right column).

Figure S21

Significant clusters across cohort-wise analyses (replicability analysis) for ***model 16*** *– CTQ sum as a predictor in medication-naïve MDD subsamples*

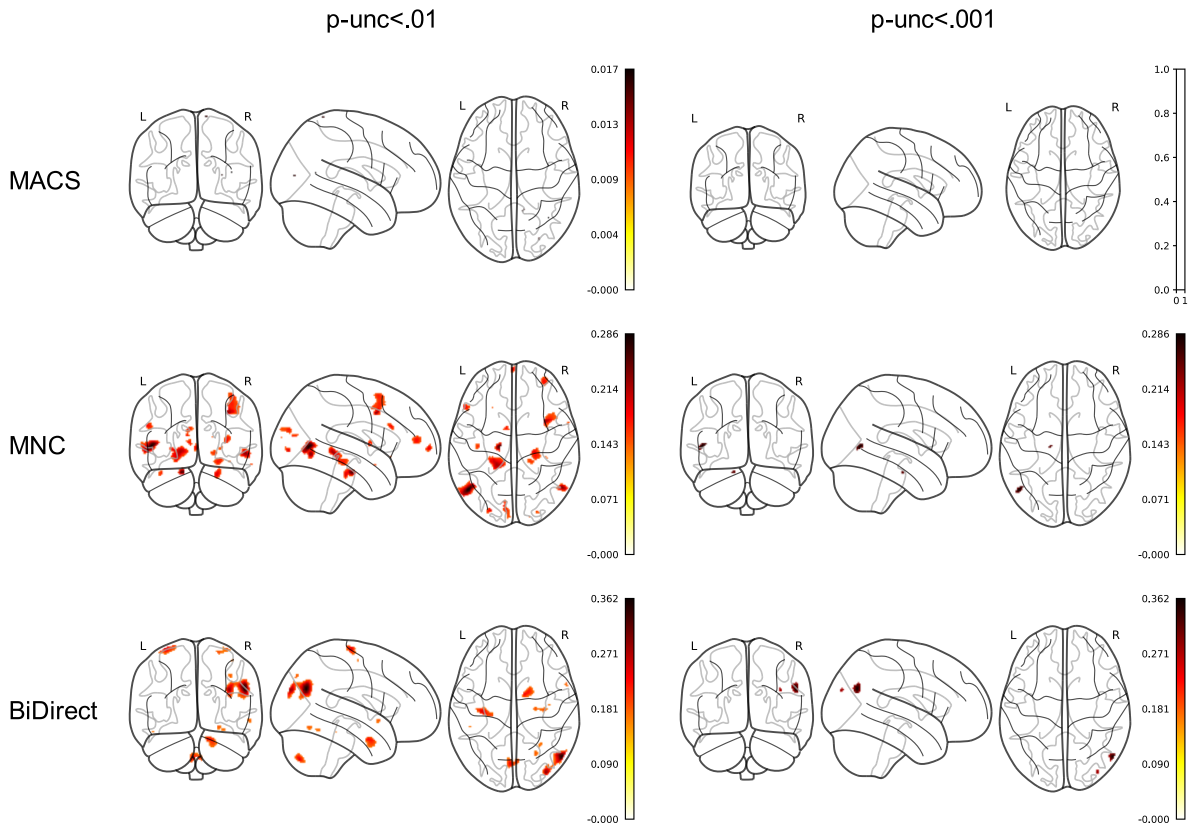

Note. Glass brains are shown with maximum intensity projections. Color bars represent the partial R^2 of the maltreatment predictor. Empty color bars indicate that no voxels survived the corresponding significance threshold. Results are shown thresholded using the uncorrected thresholds p_{unc}<.01 (left column) and p_{unc}<.001 (right column).

Figure S22

Significant clusters across cohort-wise analyses (replicability analysis) for ***model 17*** *– interaction term between CTQ sum and age as a predictor in HC subsamples*

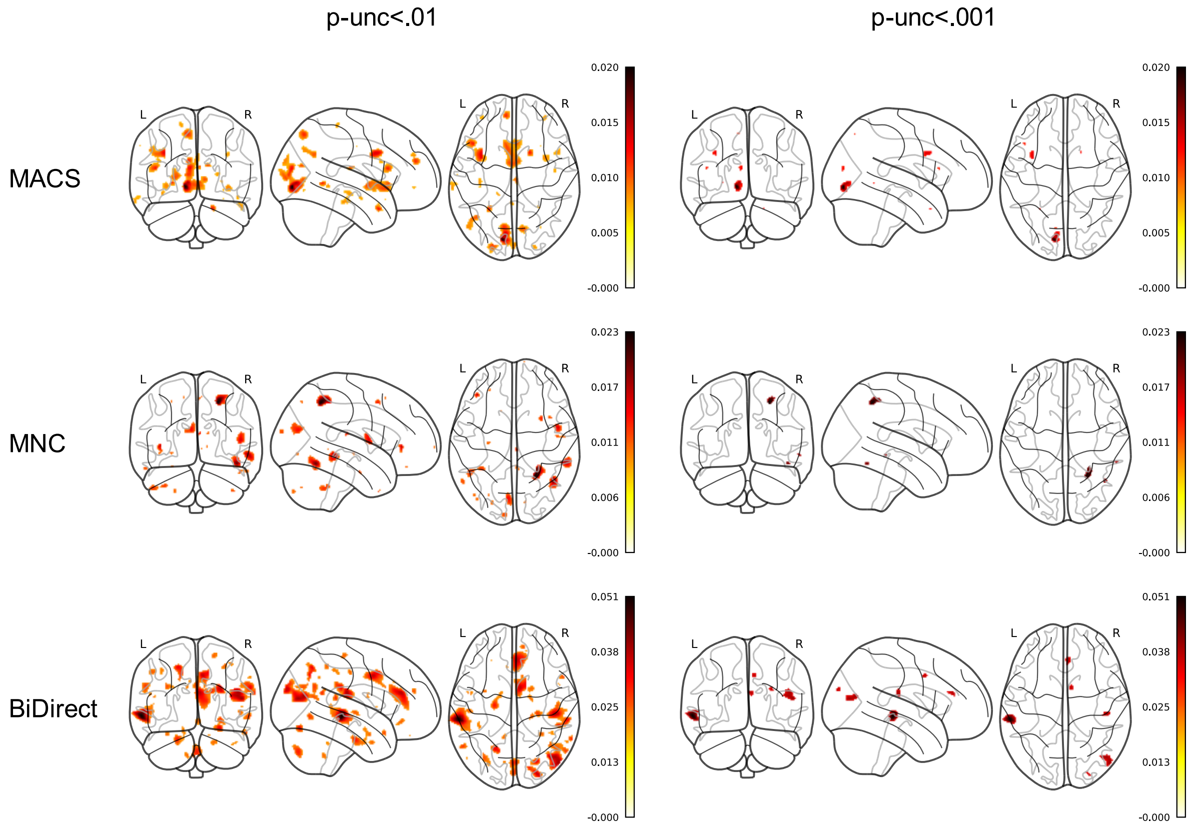

Note. Glass brains are shown with maximum intensity projections. Color bars represent the partial R^2 of the maltreatment predictor. Empty color bars indicate that no voxels survived the corresponding significance threshold. Results are shown thresholded using the uncorrected thresholds p_{unc}<.01 (left column) and p_{unc}<.001 (right column).

Figure S23

Significant clusters across cohort-wise analyses (replicability analysis) for ***model 18*** *– interaction term between CTQ sum and age as a predictor in MDD subsamples*

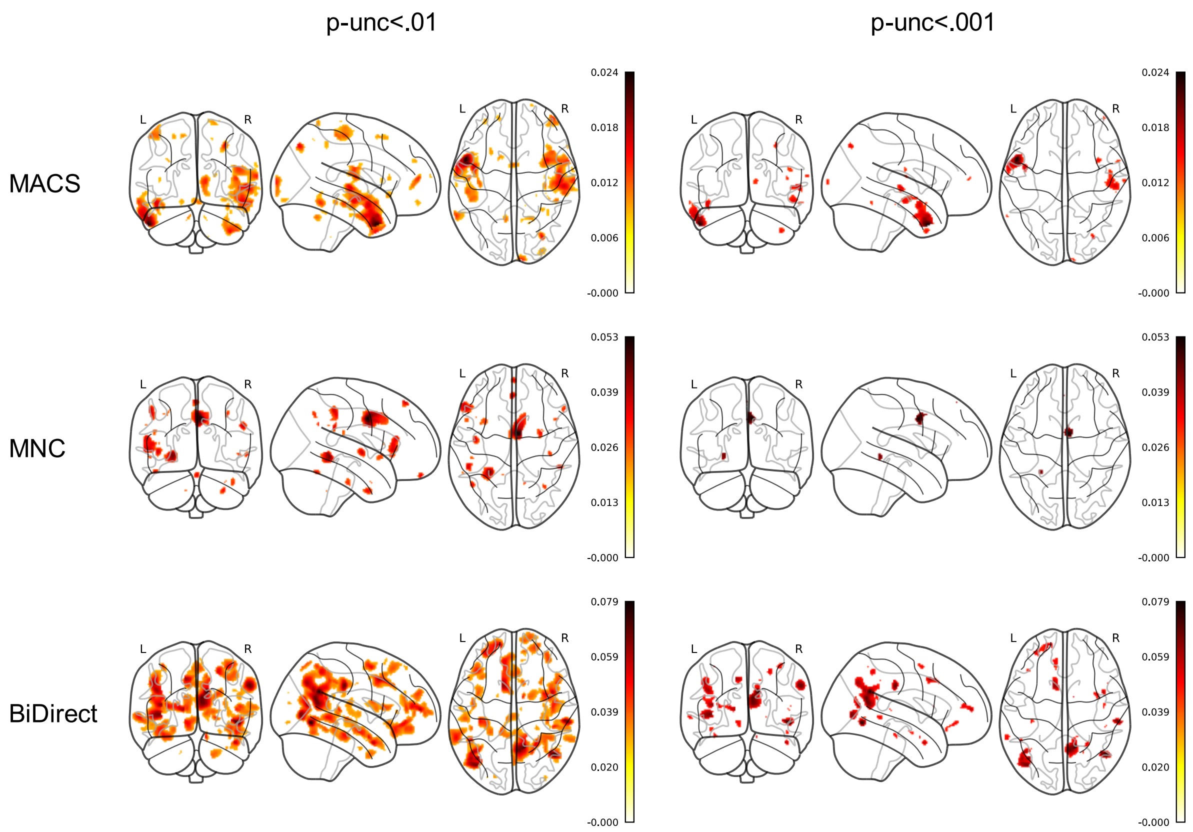

Note. Glass brains are shown with maximum intensity projections. Color bars represent the partial R^2 of the maltreatment predictor. Empty color bars indicate that no voxels survived the corresponding significance threshold. Results are shown thresholded using the uncorrected thresholds $p_{unc}<.01$ (left column) and $p_{unc}<.001$ (right column).

Figure S24

Significant clusters across cohort-wise analyses (replicability analysis) for ***model 1*** *– CTQ sum in HC and MDD samples controlling for MDD diagnosis –* ***sex-stratified for female subsample***

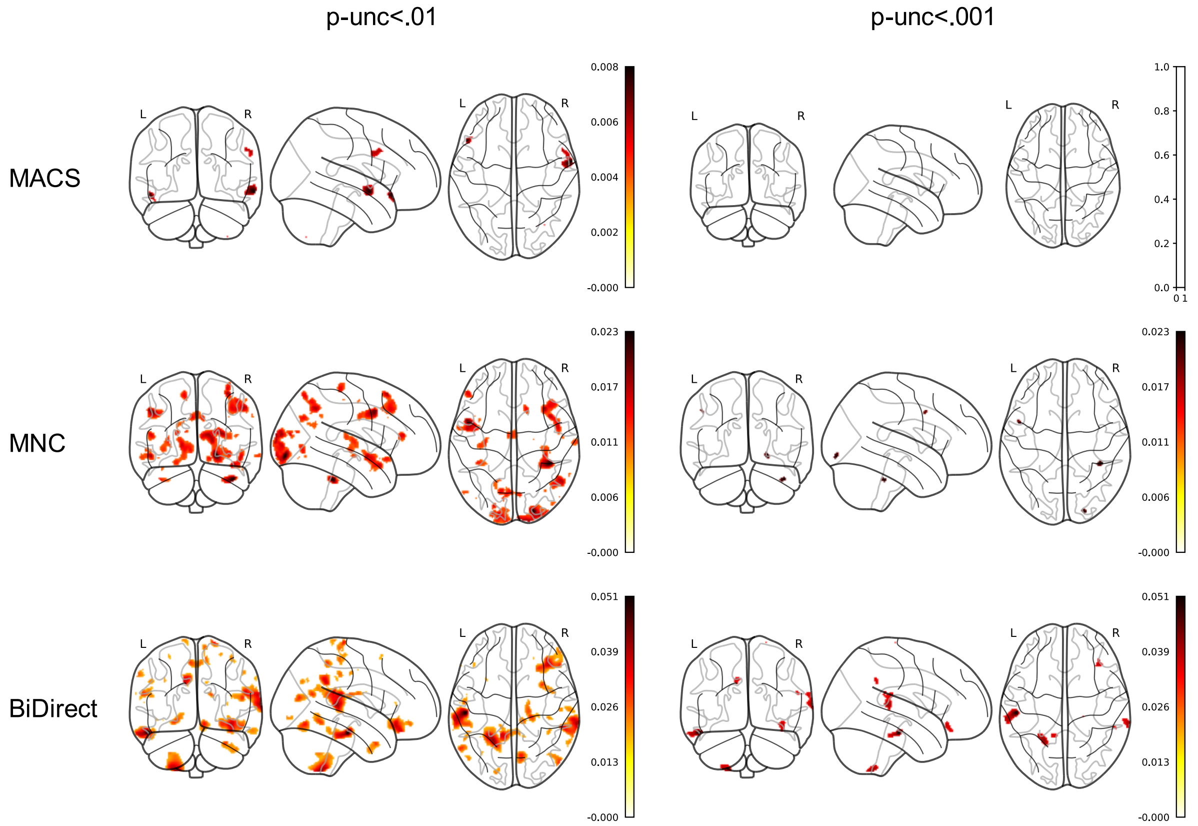

Note. Glass brains are shown with maximum intensity projections. Color bars represent the partial R^2 of the maltreatment predictor. Empty color bars indicate that no voxels survived the corresponding significance threshold. Results are shown thresholded using the uncorrected thresholds $p_{unc}<.01$ (left column) and $p_{unc}<.001$ (right column).

Figure S25

Significant clusters across cohort-wise analyses (replicability analysis) for ***model 2*** *– CTQ sum as a predictor in HC and MDD samples without controlling for MDD diagnosis –* ***sex-stratified for female subsample***

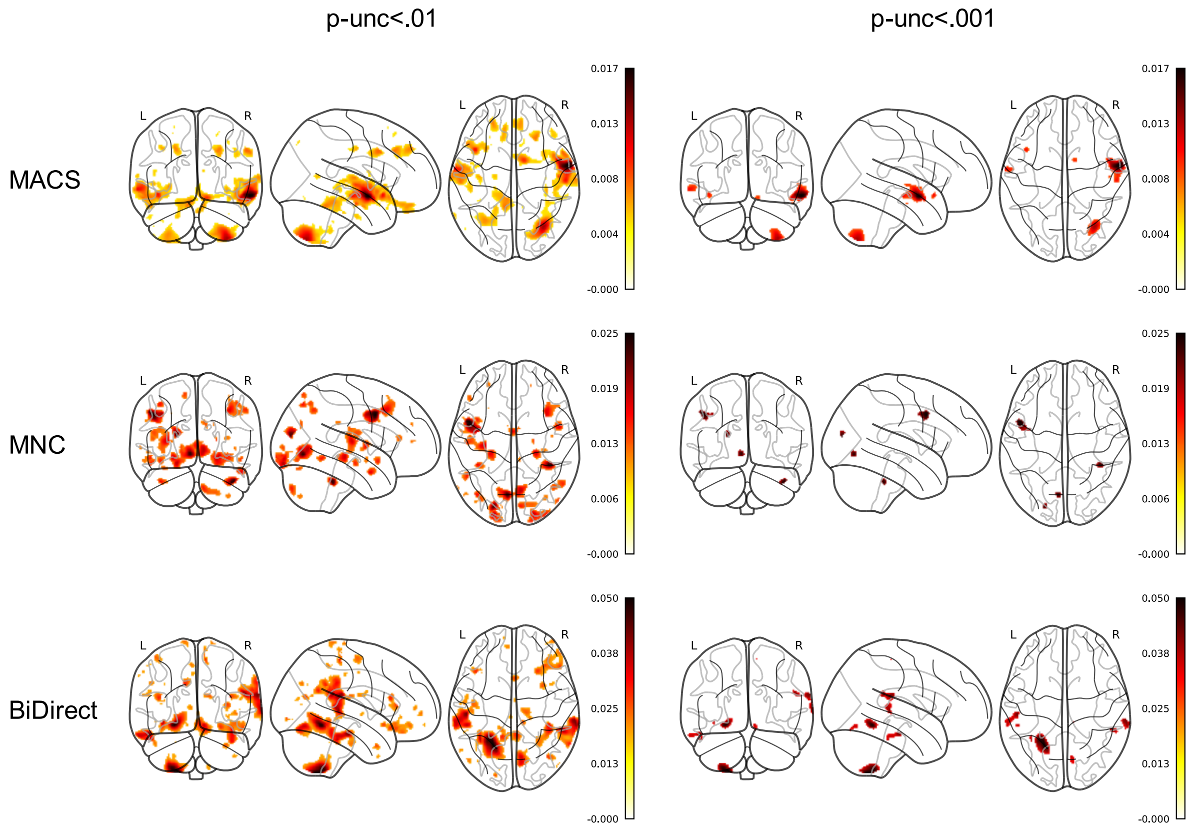

Note. Glass brains are shown with maximum intensity projections. Color bars represent the partial R^2 of the maltreatment predictor. Empty color bars indicate that no voxels survived the corresponding significance threshold. Results are shown thresholded using the uncorrected thresholds $p_{unc}<.01$ (left column) and $p_{unc}<.001$ (right column).

Figure S26

Significant clusters across cohort-wise analyses (replicability analysis) for ***model 3*** *– CTQ sum as a predictor in HC subsamples –* ***sex-stratified for female subsample***

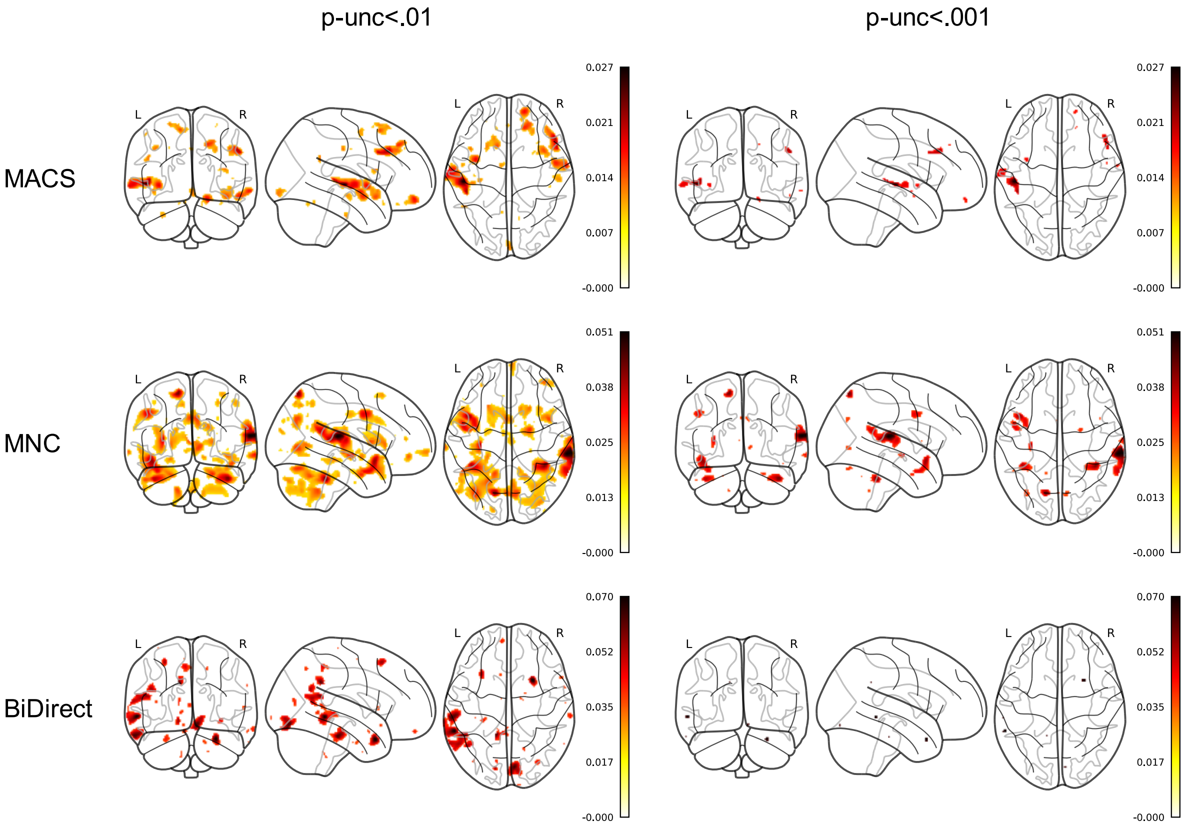

Note. Glass brains are shown with maximum intensity projections. Color bars represent the partial R^2 of the maltreatment predictor. Empty color bars indicate that no voxels survived the corresponding significance threshold. Results are shown thresholded using the uncorrected thresholds $p_{unc}<.01$ (left column) and $p_{unc}<.001$ (right column).

Figure S27

Significant clusters across cohort-wise analyses (replicability analysis) for ***model 4*** *– CTQ sum as a predictor in MDD subsamples –* ***sex-stratified for female subsample***

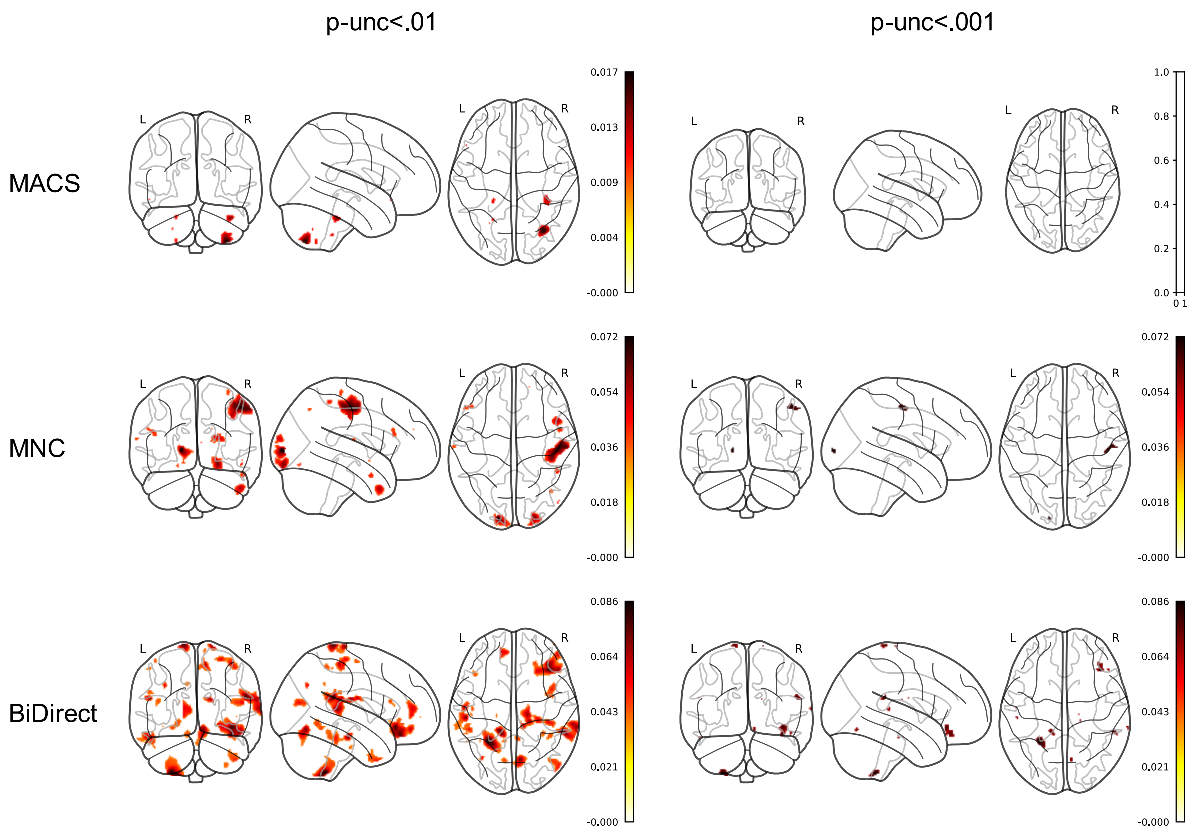

Note. Glass brains are shown with maximum intensity projections. Color bars represent the partial R^2 of the maltreatment predictor. Empty color bars indicate that no voxels survived the corresponding significance threshold. Results are shown thresholded using the uncorrected thresholds $p_{unc}<.01$ (left column) and $p_{unc}<.001$ (right column).

Figure S28

Significant clusters across cohort-wise analyses (replicability analysis) for ***model 5*** *– CTQ abuse subscales as a predictor in HC and MDD samples controlling for MDD diagnosis –* ***sex-stratified for female subsample***

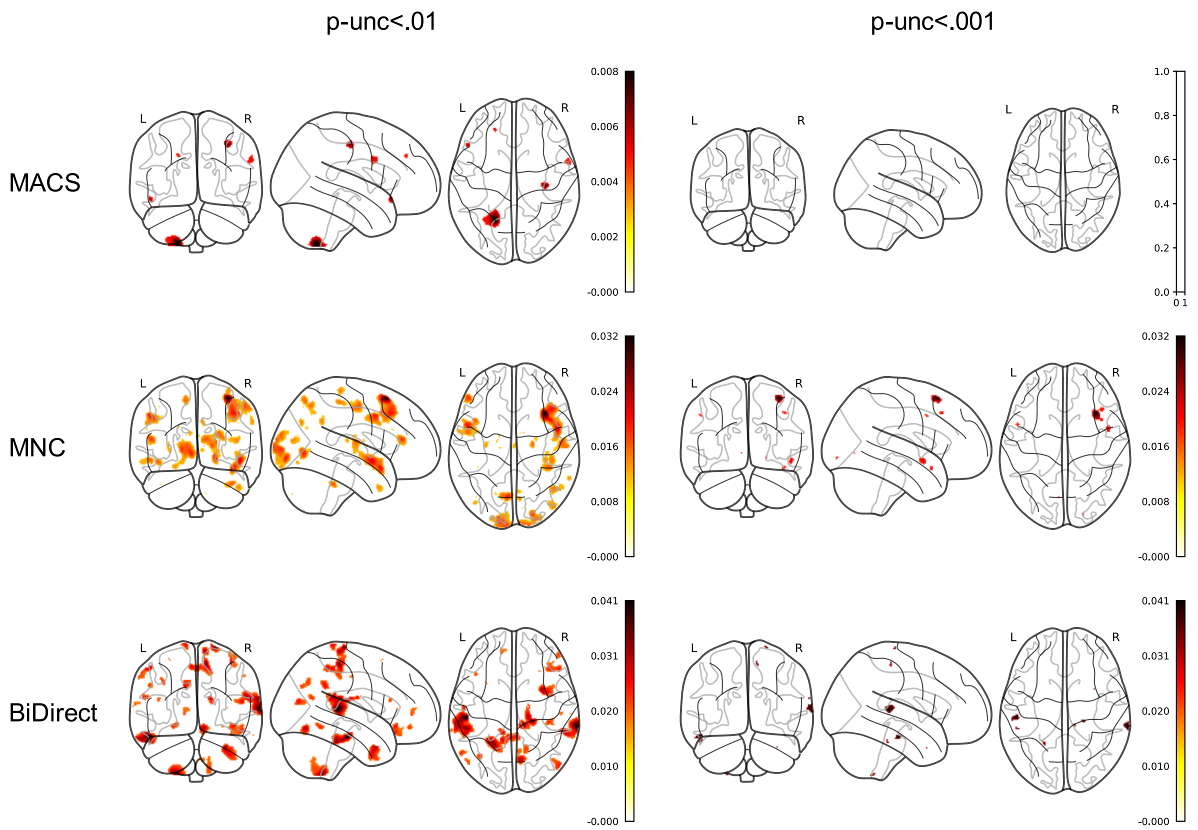

Note. Glass brains are shown with maximum intensity projections. Color bars represent the partial R^2 of the maltreatment predictor. Empty color bars indicate that no voxels survived the corresponding significance threshold. Results are shown thresholded using the uncorrected thresholds p_{unc}<.01 (left column) and p_{unc}<.001 (right column).

Figure S29

Significant clusters across cohort-wise analyses (replicability analysis) for ***model 6*** *– CTQ neglect subscales as a predictor in HC and MDD samples controlling for MDD diagnosis –* ***sex-stratified for female subsample***

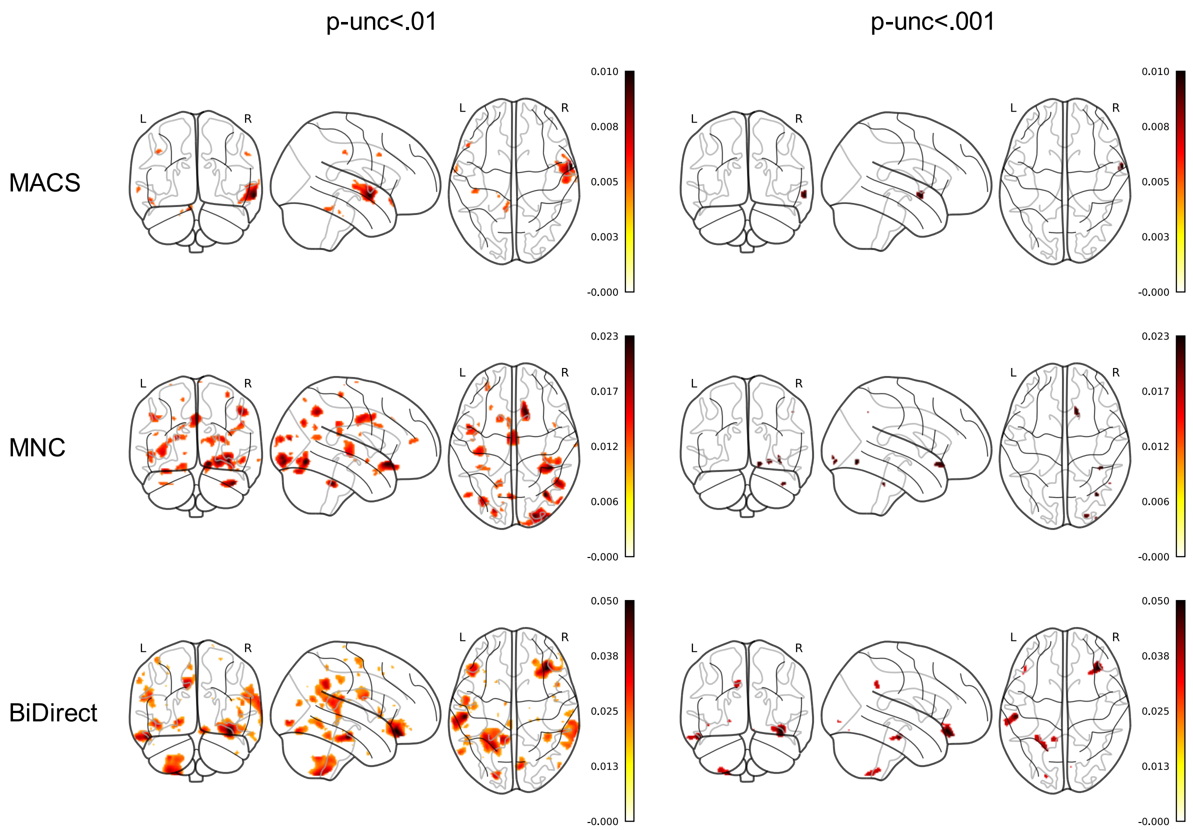

Note. Glass brains are shown with maximum intensity projections. Color bars represent the partial R^2 of the maltreatment predictor. Empty color bars indicate that no voxels survived the corresponding significance threshold. Results are shown thresholded using the uncorrected thresholds $p_{unc}<.01$ (left column) and $p_{unc}<.001$ (right column).

Figure S30

*Significant clusters across cohort-wise analyses (replicability analysis) for **model 7** – CTQ subscale emotional abuse as a predictor in HC and MDD samples controlling for MDD diagnosis – **sex-stratified for female subsample***

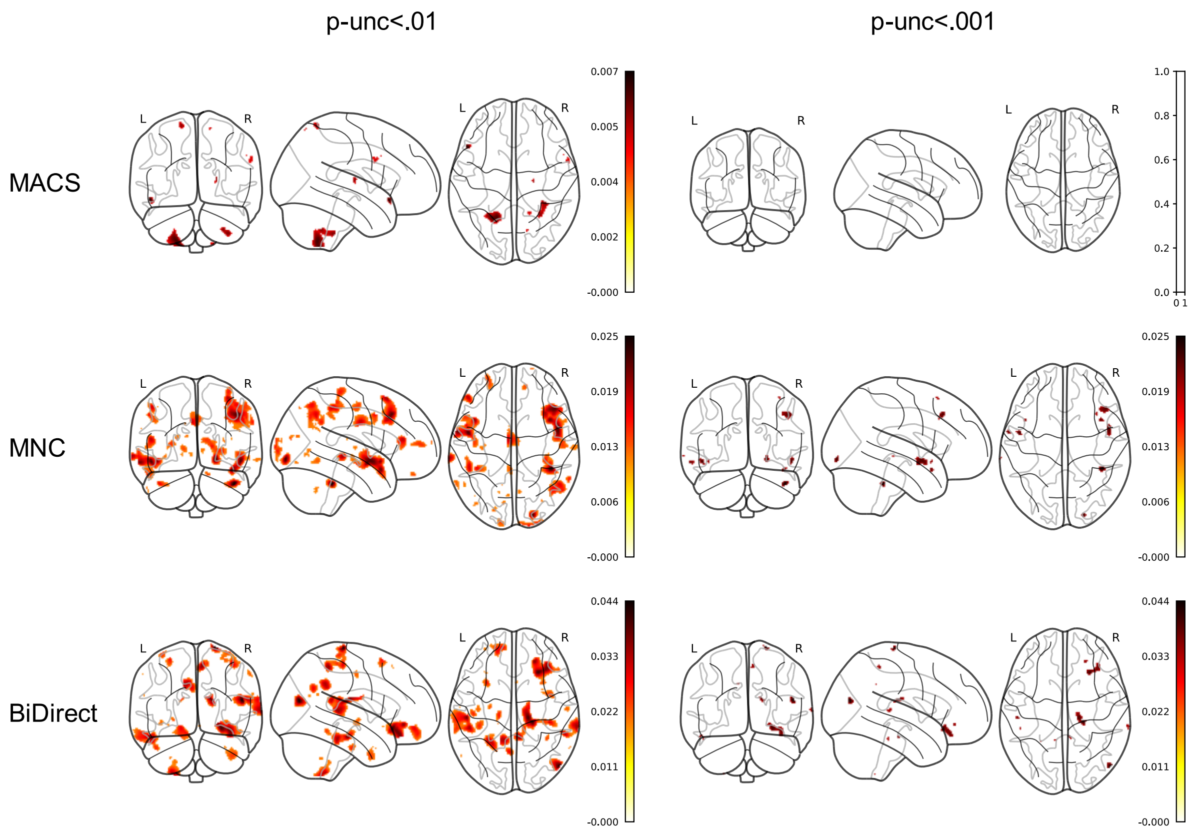

Note. Glass brains are shown with maximum intensity projections. Color bars represent the partial R^2 of the maltreatment predictor. Empty color bars indicate that no voxels survived the corresponding significance threshold. Results are shown thresholded using the uncorrected thresholds p_{unc}<.01 (left column) and p_{unc}<.001 (right column).

Figure S31

Significant clusters across cohort-wise analyses (replicability analysis) for ***model 8*** *– CTQ subscale physical abuse as a predictor in HC and MDD samples controlling for MDD diagnosis –* ***sex-stratified for female subsample***

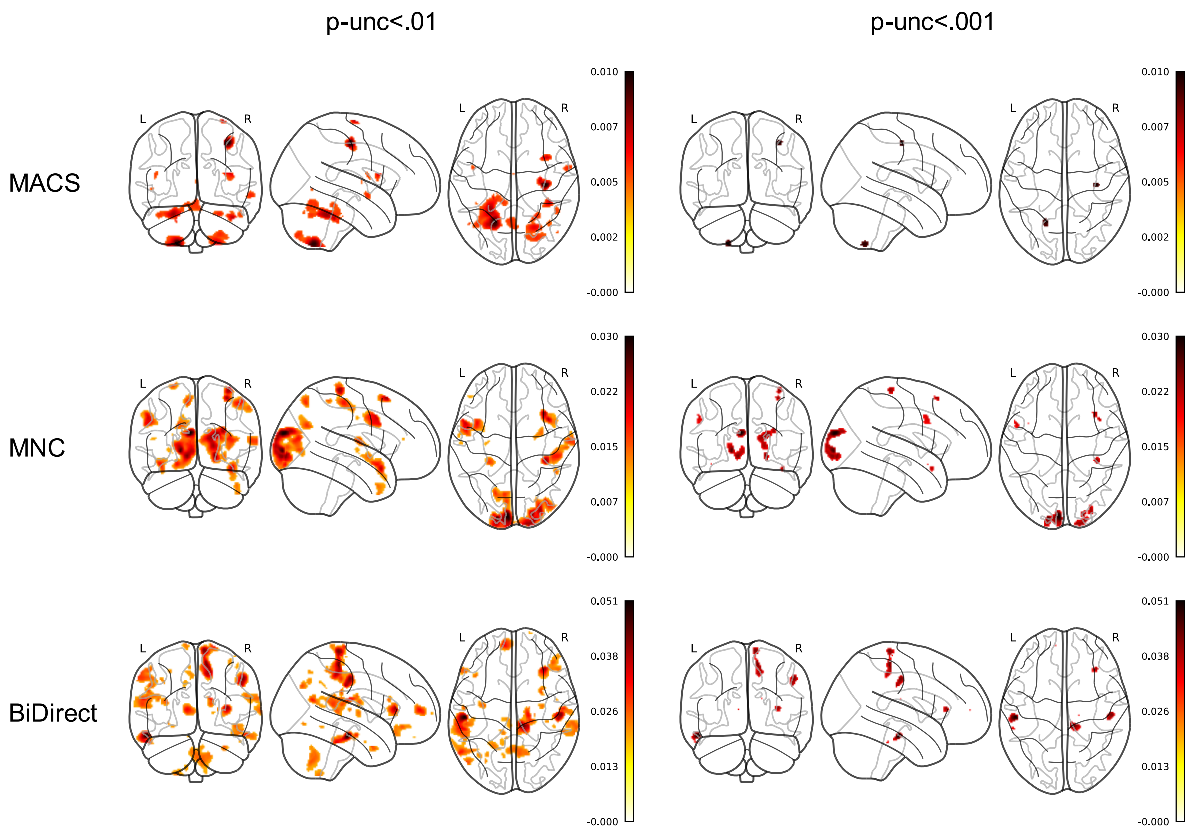

Note. Glass brains are shown with maximum intensity projections. Color bars represent the partial R^2 of the maltreatment predictor. Empty color bars indicate that no voxels survived the corresponding significance threshold. Results are shown thresholded using the uncorrected thresholds p_{unc}<.01 (left column) and p_{unc}<.001 (right column).

Figure S32

Significant clusters across cohort-wise analyses (replicability analysis) for ***model 9*** *– CTQ subscale sexual abuse as a predictor in HC and MDD samples controlling for MDD diagnosis –* ***sex-stratified for female subsample***

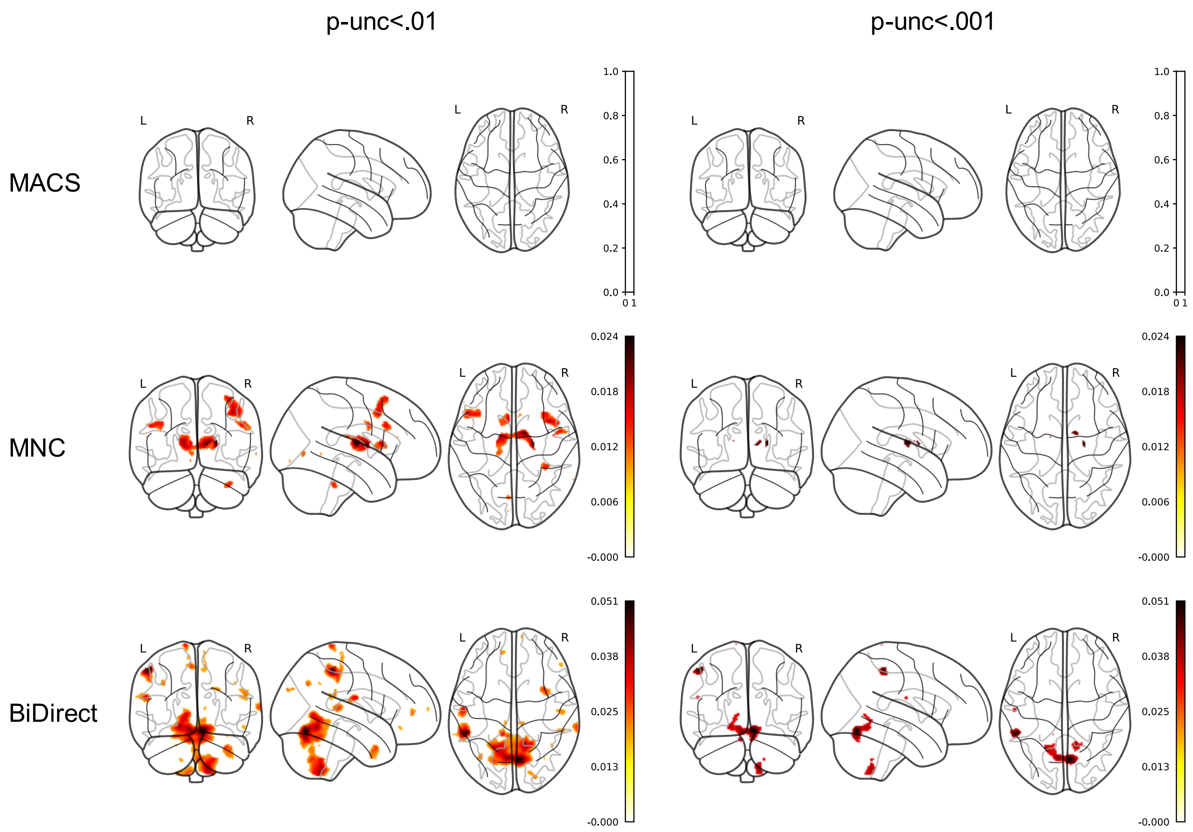

Note. Glass brains are shown with maximum intensity projections. Color bars represent the partial R^2 of the maltreatment predictor. Empty color bars indicate that no voxels survived the corresponding significance threshold. Results are shown thresholded using the uncorrected thresholds $p_{unc}<.01$ (left column) and $p_{unc}<.001$ (right column).

Figure S33

Significant clusters across cohort-wise analyses (replicability analysis) for ***model 10*** *– CTQ subscale emotional neglect as a predictor in HC and MDD samples controlling for MDD diagnosis –* ***sex-stratified for female subsample***

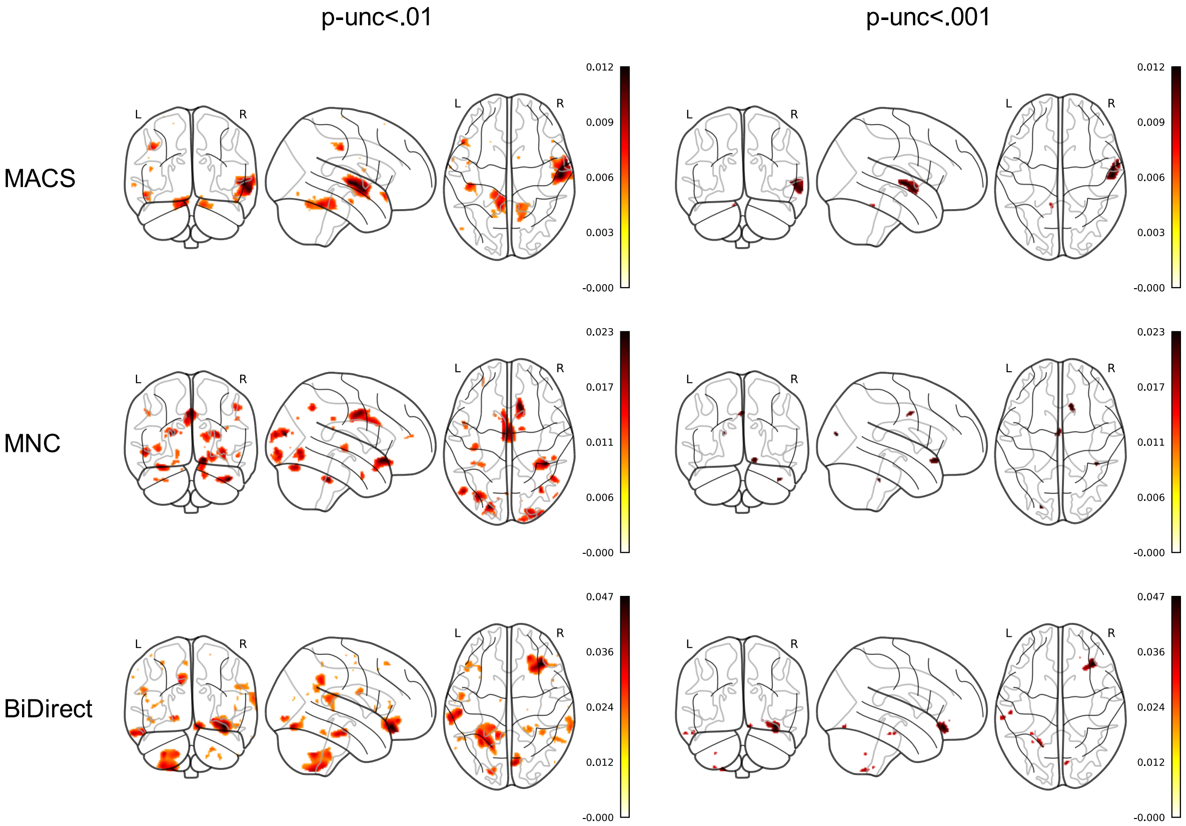

Note. Glass brains are shown with maximum intensity projections. Color bars represent the partial R^2 of the maltreatment predictor. Empty color bars indicate that no voxels survived the corresponding significance threshold. Results are shown thresholded using the uncorrected thresholds p_{unc}<.01 (left column) and p_{unc}<.001 (right column).

Figure S34

Significant clusters across cohort-wise analyses (replicability analysis) for ***model 11*** *– CTQ subscale physical neglect as a predictor in HC and MDD samples controlling for MDD diagnosis –* ***sex-stratified for female subsample***

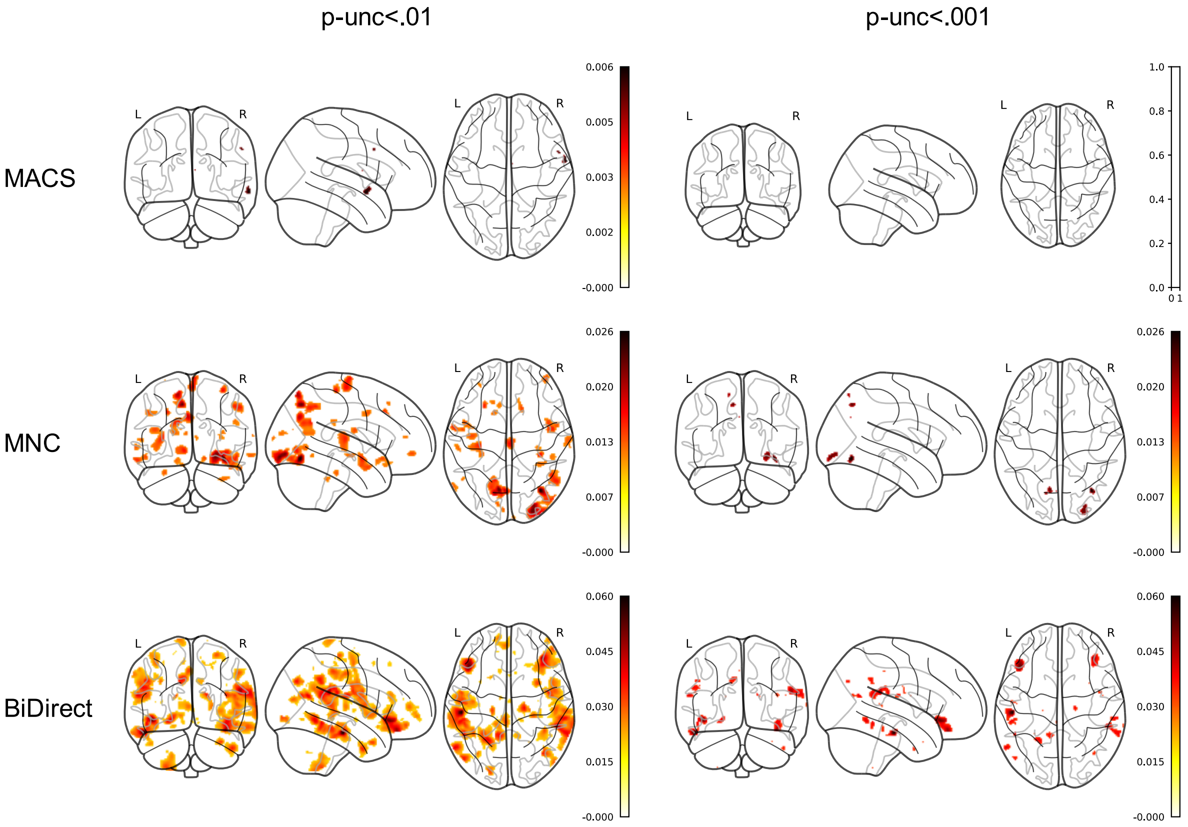

Note. Glass brains are shown with maximum intensity projections. Color bars represent the partial R^2 of the maltreatment predictor. Empty color bars indicate that no voxels survived the corresponding significance threshold. Results are shown thresholded using the uncorrected thresholds $p_{unc}<.01$ (left column) and $p_{unc}<.001$ (right column).

Figure S35

Significant clusters across cohort-wise analyses (replicability analysis) for ***model 12*** *– CTQ extreme severity compared to none in HC and MDD samples controlling for MDD diagnosis –* ***sex-stratified for female subsample***

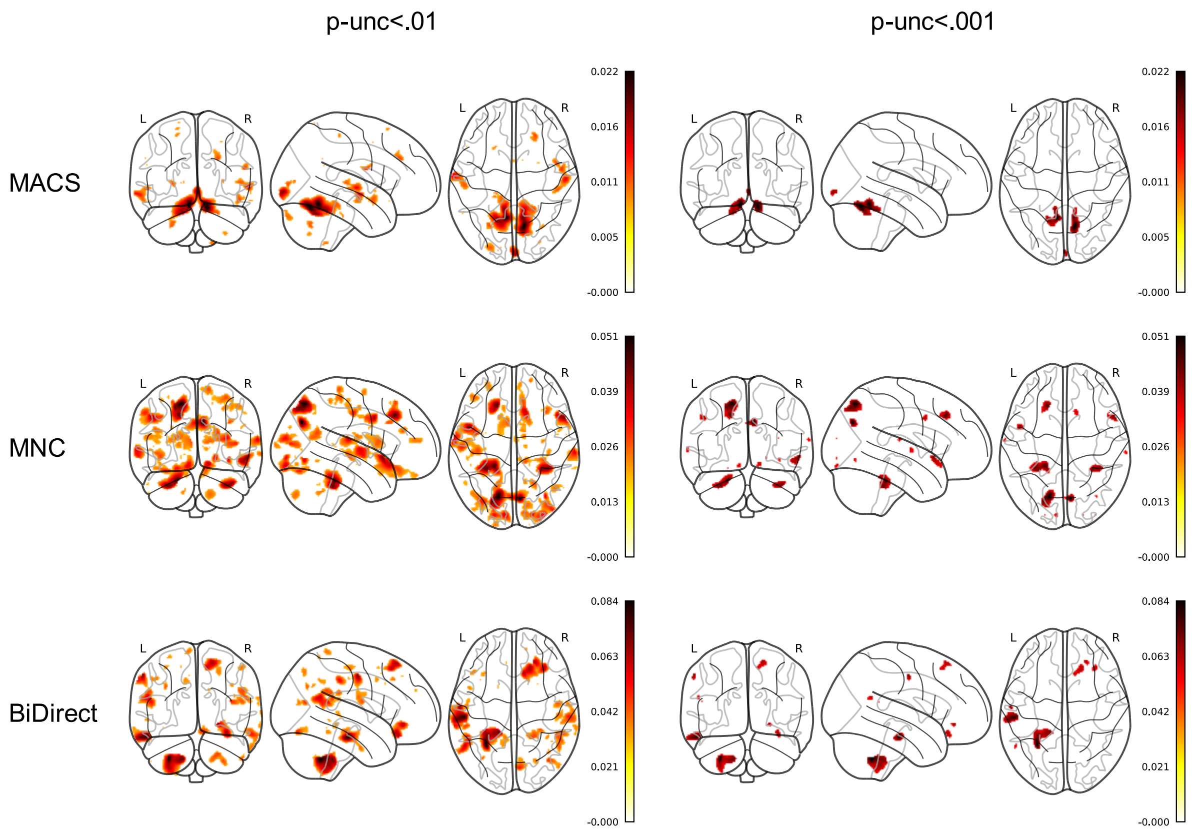

Note. Glass brains are shown with maximum intensity projections. Color bars represent the partial R^2 of the maltreatment predictor. Empty color bars indicate that no voxels survived the corresponding significance threshold. Results are shown thresholded using the uncorrected thresholds $p_{unc}<.01$ (left column) and $p_{unc}<.001$ (right column).

Figure S36

*Significant clusters across cohort-wise analyses (replicability analysis) for **model 13** – CTQ extreme severity compared to none in HC and MDD samples without controlling for MDD diagnosis – **sex-stratified for female subsample***

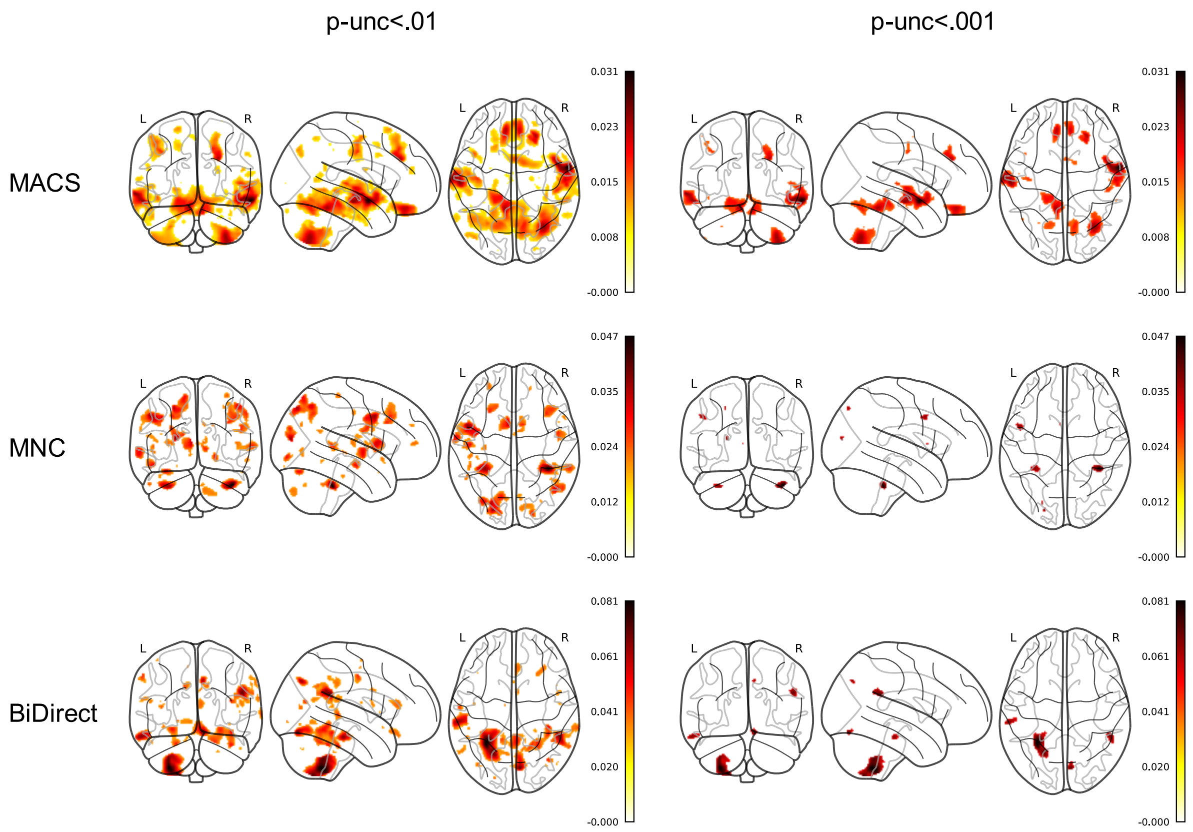

Note. Glass brains are shown with maximum intensity projections. Color bars represent the partial R^2 of the maltreatment predictor. Empty color bars indicate that no voxels survived the corresponding significance threshold. Results are shown thresholded using the uncorrected thresholds $p_{unc}<.01$ (left column) and $p_{unc}<.001$ (right column).

Figure S37

*Significant clusters across cohort-wise analyses (replicability analysis) for **model 14** – CTQ extreme severity compared to none in HC subsamples – **sex-stratified for female subsample***

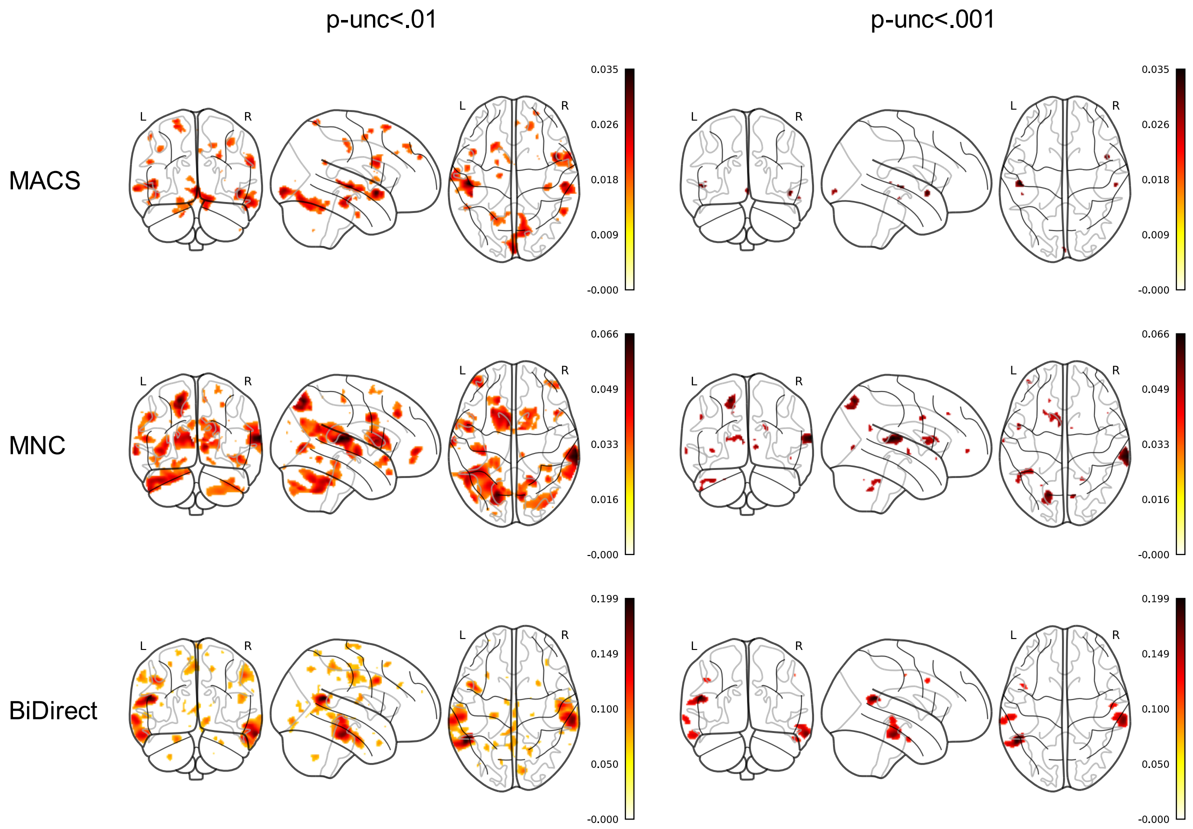

Note. Glass brains are shown with maximum intensity projections. Color bars represent the partial R^2 of the maltreatment predictor. Empty color bars indicate that no voxels survived the corresponding significance threshold. Results are shown thresholded using the uncorrected thresholds $p_{unc}<.01$ (left column) and $p_{unc}<.001$ (right column).

Figure S38

Significant clusters across cohort-wise analyses (replicability analysis) for ***model 15*** *– CTQ extreme severity compared to none in MDD subsamples –* ***sex-stratified for female subsample***

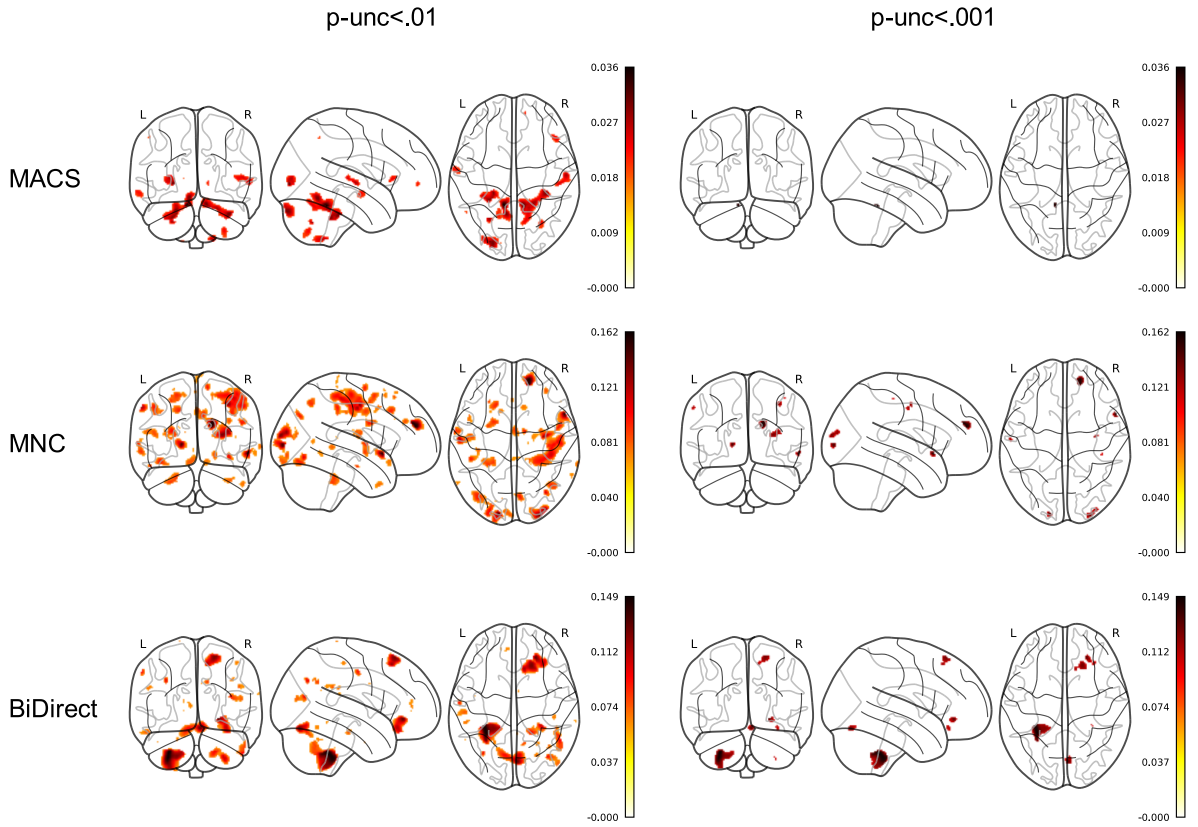

Note. Glass brains are shown with maximum intensity projections. Color bars represent the partial R^2 of the maltreatment predictor. Empty color bars indicate that no voxels survived the corresponding significance threshold. Results are shown thresholded using the uncorrected thresholds $p_{unc}<.01$ (left column) and $p_{unc}<.001$ (right column).

Figure S39

Significant clusters across cohort-wise analyses (replicability analysis) for ***model 16*** *– CTQ sum as a predictor in medication-naïve MDD subsamples –* ***sex-stratified for female subsample***

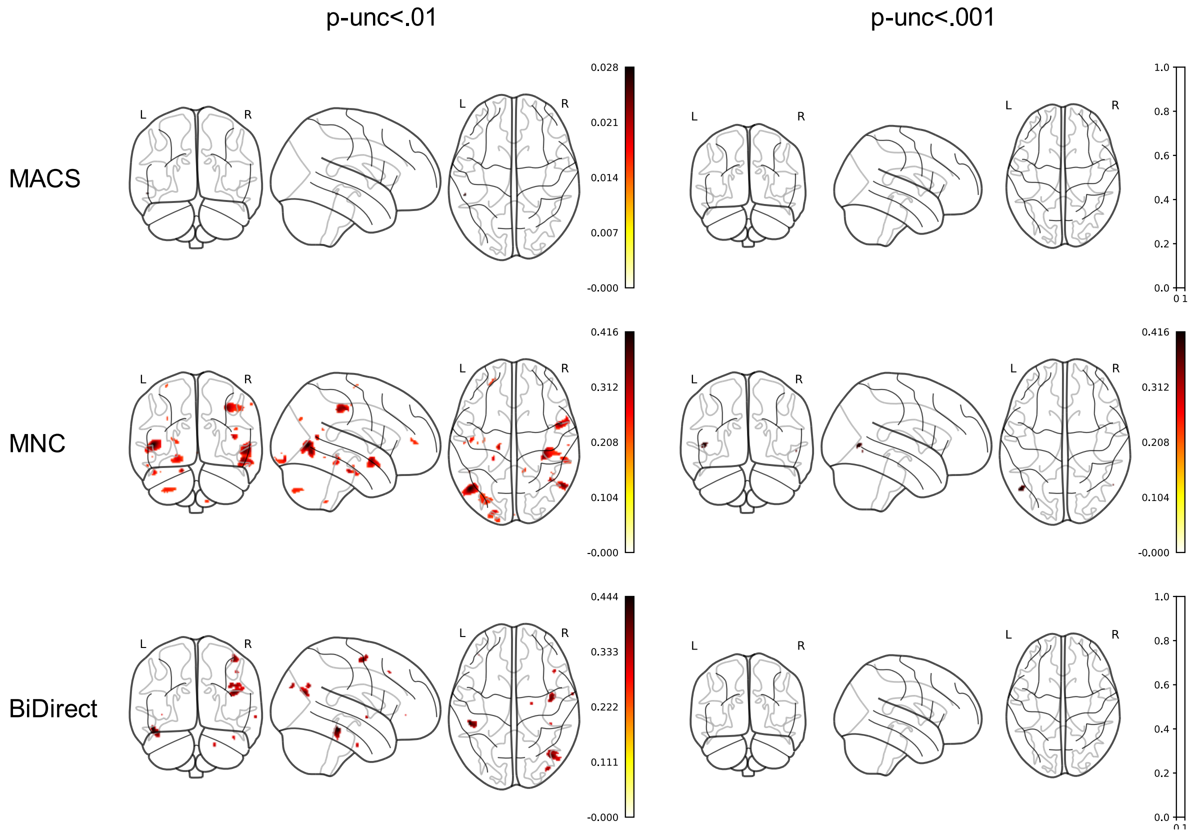

Note. Glass brains are shown with maximum intensity projections. Color bars represent the partial R^2 of the maltreatment predictor. Empty color bars indicate that no voxels survived the corresponding significance threshold. Results are shown thresholded using the uncorrected thresholds $p_{unc}<.01$ (left column) and $p_{unc}<.001$ (right column).

Figure S40

Significant clusters across cohort-wise analyses (replicability analysis) for ***model 17*** *– interaction term between CTQ sum and age as a predictor in HC subsamples –* ***sex-stratified for female subsample***

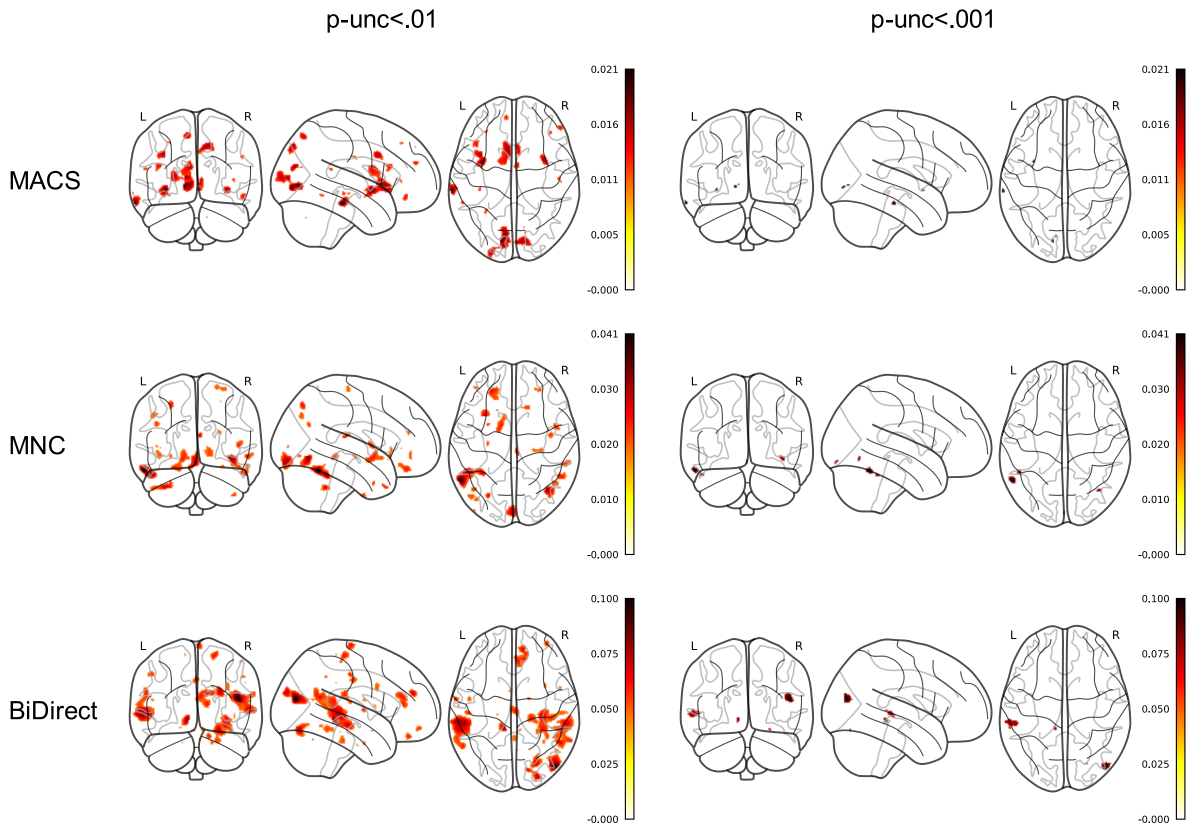

Note. Glass brains are shown with maximum intensity projections. Color bars represent the partial R^2 of the maltreatment predictor. Empty color bars indicate that no voxels survived the corresponding significance threshold. Results are shown thresholded using the uncorrected thresholds p_{unc}<.01 (left column) and p_{unc}<.001 (right column).

Figure S41

Significant clusters across cohort-wise analyses (replicability analysis) for ***model 18*** *– interaction term between CTQ sum and age as a predictor in MDD subsamples –* ***sex-stratified for female subsample***

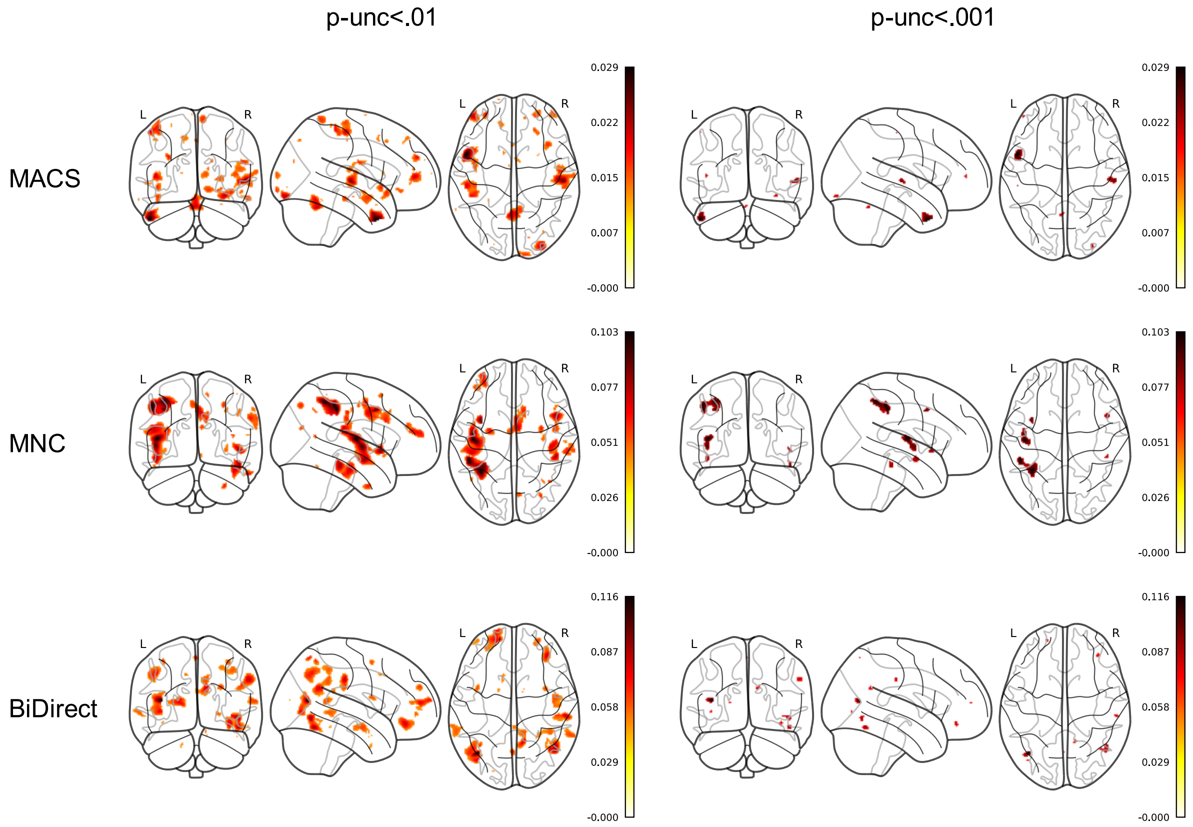

Note. Glass brains are shown with maximum intensity projections. Color bars represent the partial R^2 of the maltreatment predictor. Empty color bars indicate that no voxels survived the corresponding significance threshold. Results are shown thresholded using the uncorrected thresholds p_{unc}<.01 (left column) and p_{unc}<.001 (right column).

Figure S42

*Significant clusters across cohort-wise analyses (replicability analysis) for **model 1** – CTQ sum in HC and MDD samples controlling for MDD diagnosis – **sex-stratified for male subsample***

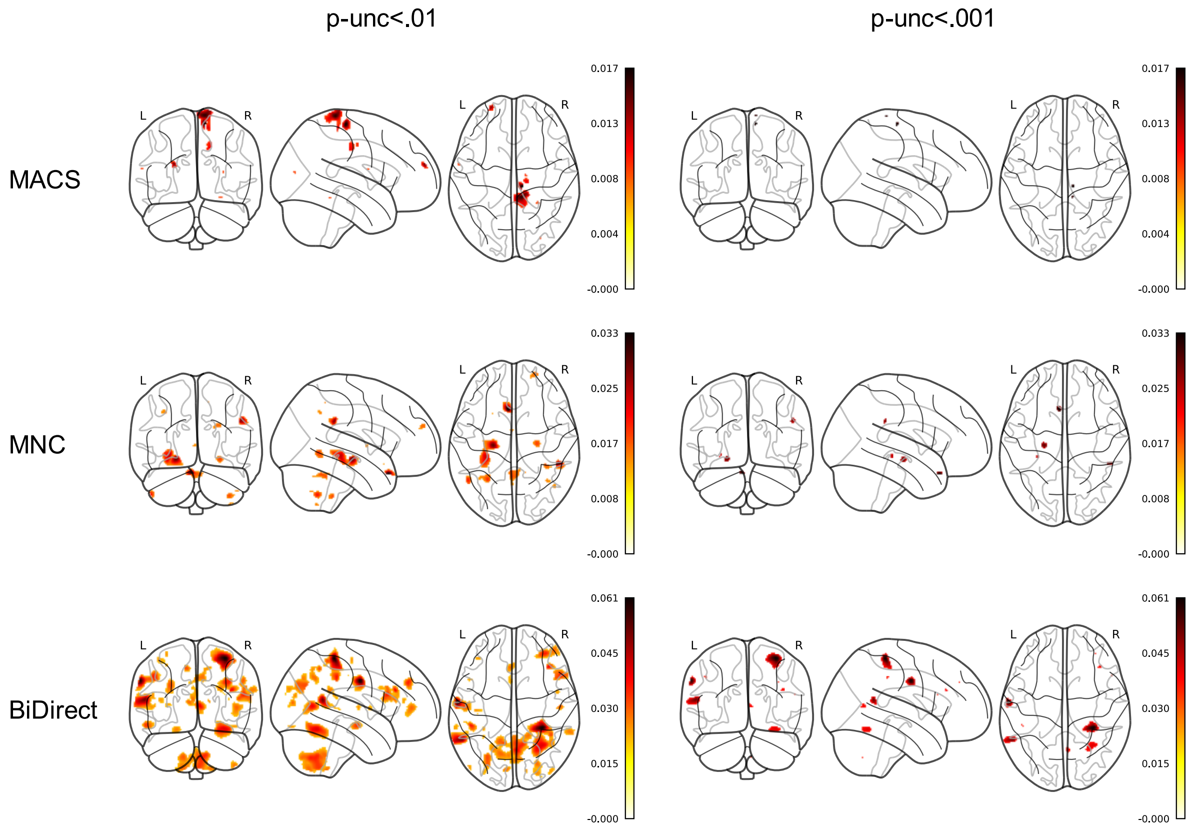

Note. Glass brains are shown with maximum intensity projections. Color bars represent the partial R^2 of the maltreatment predictor. Empty color bars indicate that no voxels survived the corresponding significance threshold. Results are shown thresholded using the uncorrected thresholds $p_{unc}<.01$ (left column) and $p_{unc}<.001$ (right column).

Figure S43

Significant clusters across cohort-wise analyses (replicability analysis) for ***model 2*** *– CTQ sum as a predictor in HC and MDD samples without controlling for MDD diagnosis –* ***sex-stratified for male subsample***

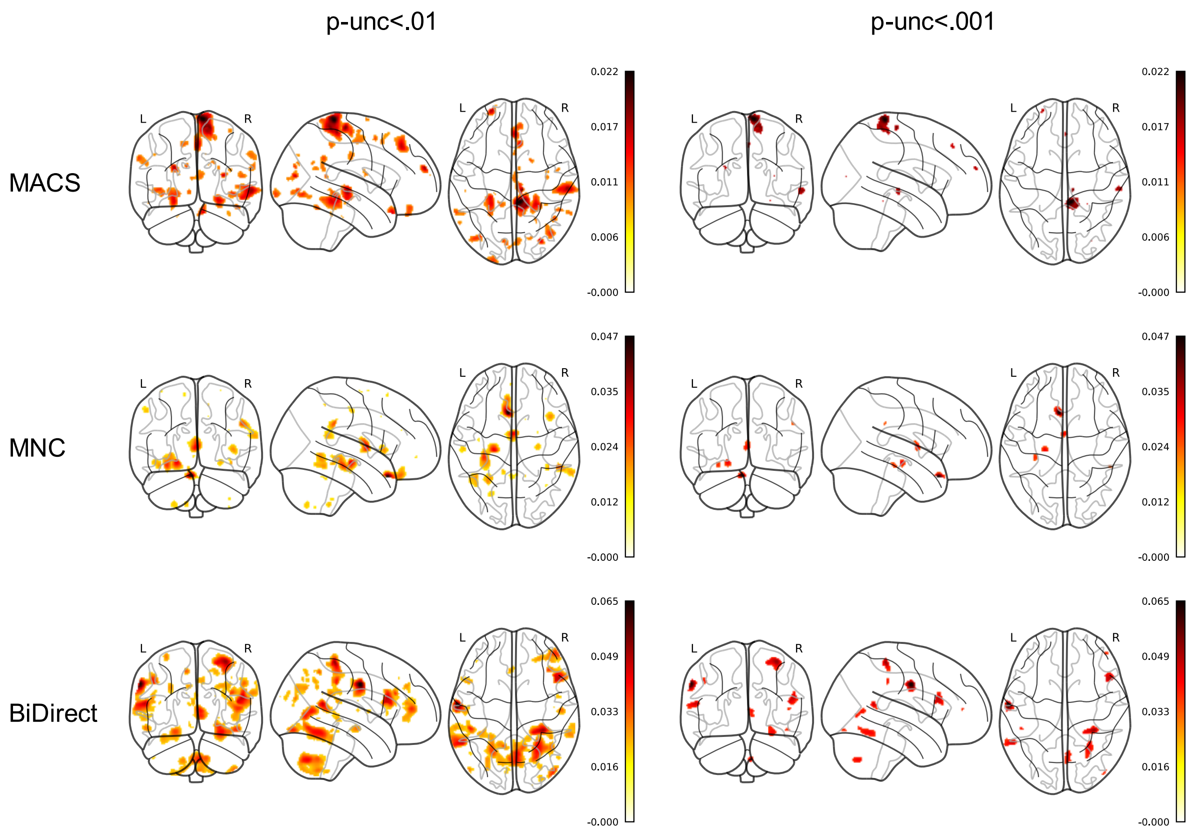

Note. Glass brains are shown with maximum intensity projections. Color bars represent the partial R^2 of the maltreatment predictor. Empty color bars indicate that no voxels survived the corresponding significance threshold. Results are shown thresholded using the uncorrected thresholds $p_{unc}<.01$ (left column) and $p_{unc}<.001$ (right column).

Figure S44

*Significant clusters across cohort-wise analyses (replicability analysis) for **model 3** – CTQ sum as a predictor in HC subsamples – **sex-stratified for male subsample***

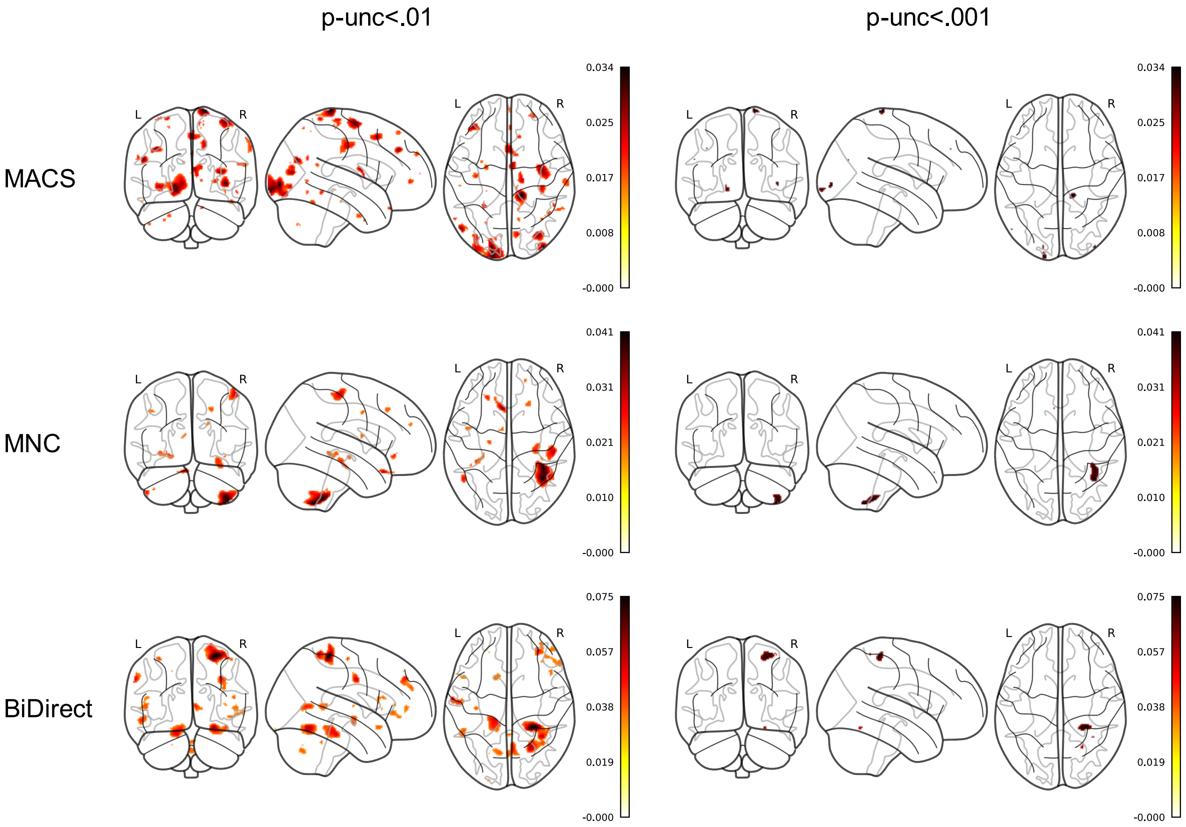

Note. Glass brains are shown with maximum intensity projections. Color bars represent the partial R^2 of the maltreatment predictor. Empty color bars indicate that no voxels survived the corresponding significance threshold. Results are shown thresholded using the uncorrected thresholds $p_{unc}<.01$ (left column) and $p_{unc}<.001$ (right column).

Figure S45

*Significant clusters across cohort-wise analyses (replicability analysis) for **model 4** – CTQ sum as a predictor in MDD subsamples – **sex-stratified for male subsample***

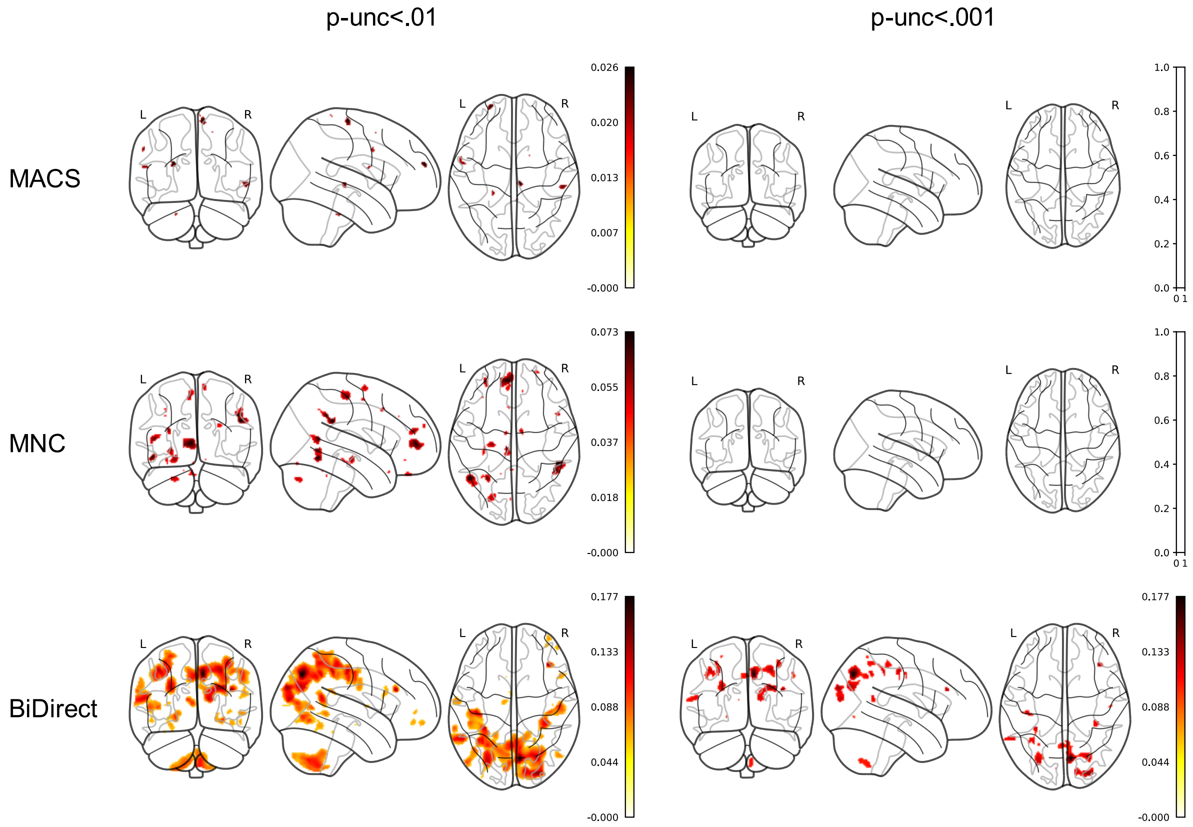

Note. Glass brains are shown with maximum intensity projections. Color bars represent the partial R^2 of the maltreatment predictor. Empty color bars indicate that no voxels survived the corresponding significance threshold. Results are shown thresholded using the uncorrected thresholds $p_{unc}<.01$ (left column) and $p_{unc}<.001$ (right column).

Figure S46

Significant clusters across cohort-wise analyses (replicability analysis) for ***model 5*** *– CTQ abuse subscales as a predictor in HC and MDD samples controlling for MDD diagnosis –* ***sex-stratified for male subsample***

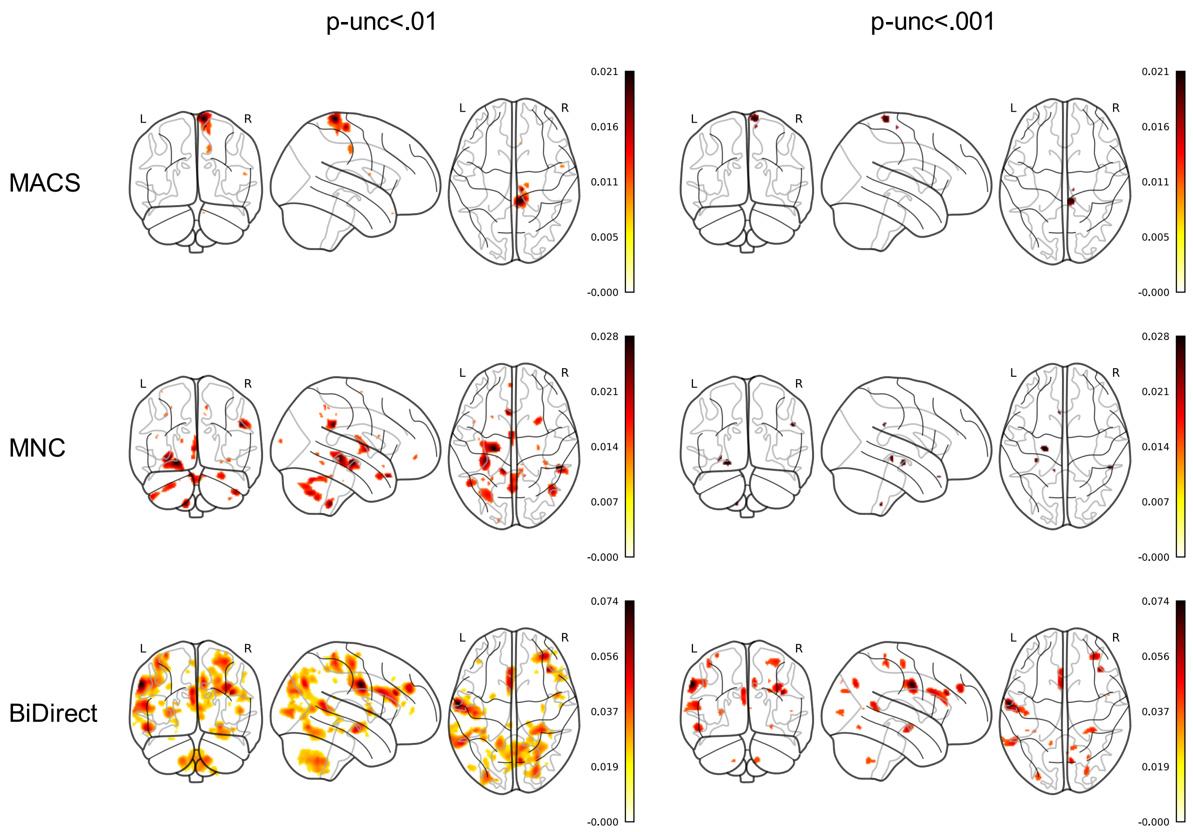

Note. Glass brains are shown with maximum intensity projections. Color bars represent the partial R^2 of the maltreatment predictor. Empty color bars indicate that no voxels survived the corresponding significance threshold. Results are shown thresholded using the uncorrected thresholds $p_{unc}<.01$ (left column) and $p_{unc}<.001$ (right column).

Figure S47

Significant clusters across cohort-wise analyses (replicability analysis) for ***model 6*** *– CTQ neglect subscales as a predictor in HC and MDD samples controlling for MDD diagnosis –* ***sex-stratified for male subsample***

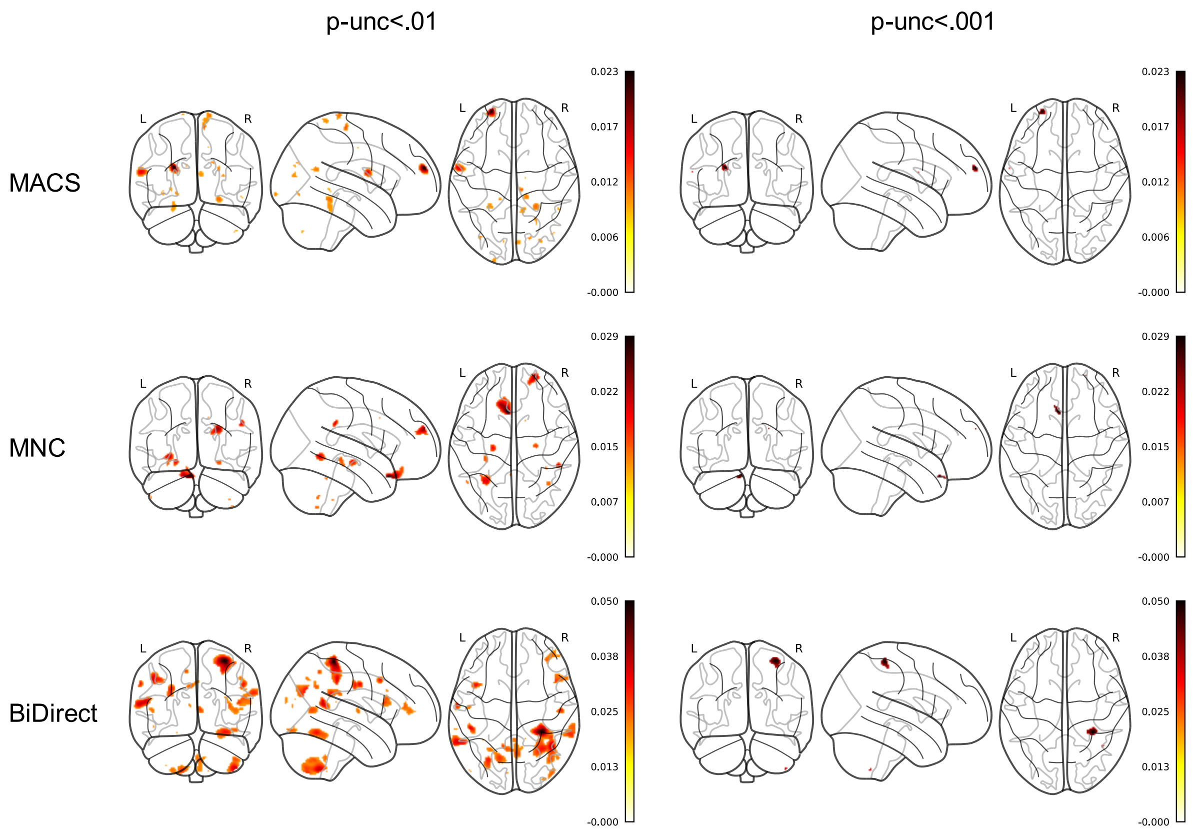

Note. Glass brains are shown with maximum intensity projections. Color bars represent the partial R^2 of the maltreatment predictor. Empty color bars indicate that no voxels survived the corresponding significance threshold. Results are shown thresholded using the uncorrected thresholds $p_{unc}<.01$ (left column) and $p_{unc}<.001$ (right column).

Figure S48

*Significant clusters across cohort-wise analyses (replicability analysis) for **model 7** – CTQ subscale emotional abuse as a predictor in HC and MDD samples controlling for MDD diagnosis – **sex-stratified for male subsample***

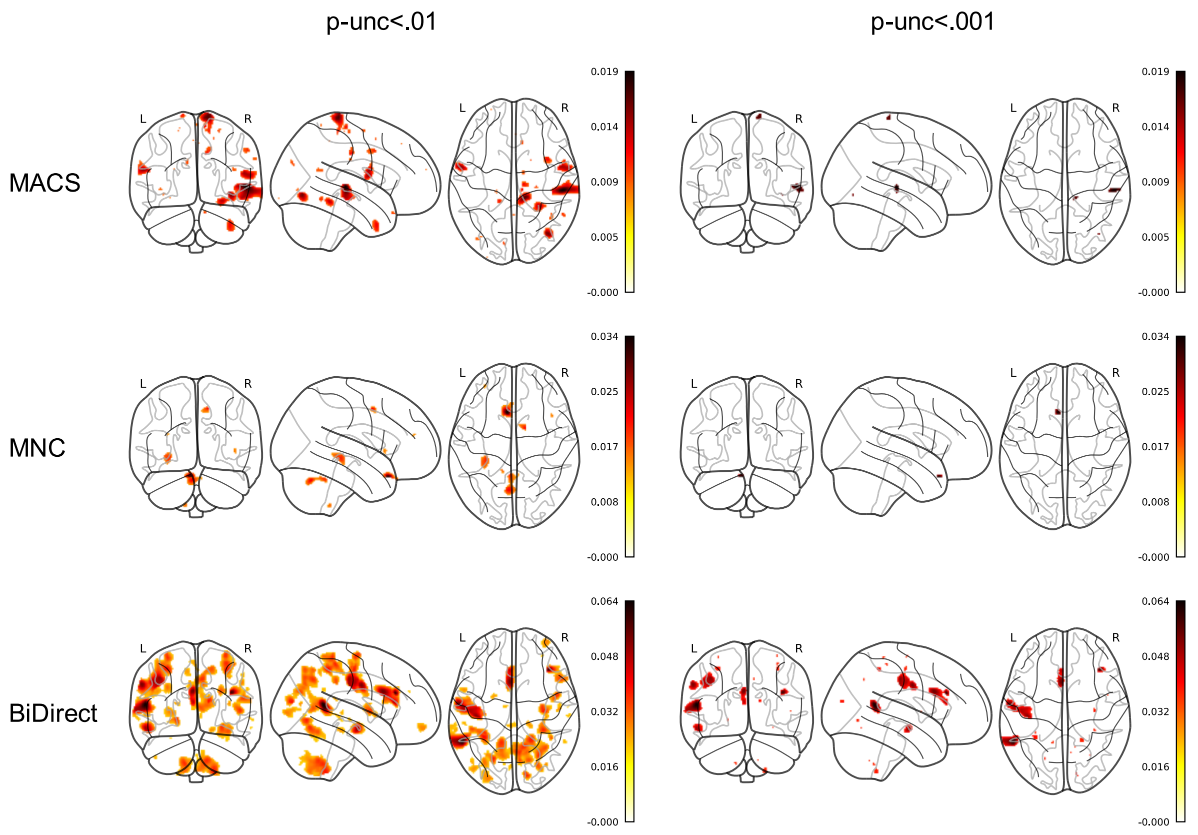

Note. Glass brains are shown with maximum intensity projections. Color bars represent the partial R^2 of the maltreatment predictor. Empty color bars indicate that no voxels survived the corresponding significance threshold. Results are shown thresholded using the uncorrected thresholds p_{unc}<.01 (left column) and p_{unc}<.001 (right column).

Figure S49

*Significant clusters across cohort-wise analyses (replicability analysis) for **model 8** – CTQ subscale physical abuse as a predictor in HC and MDD samples controlling for MDD diagnosis – **sex-stratified for male subsample***

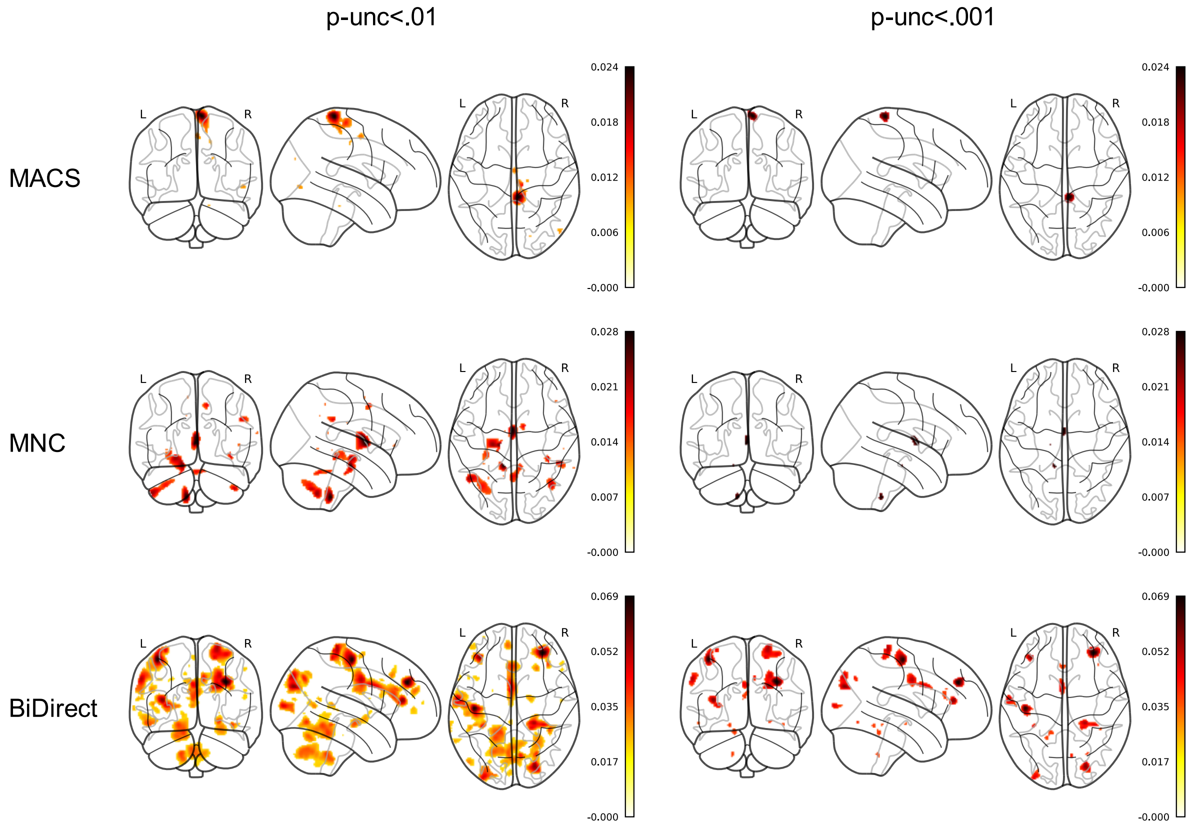

Note. Glass brains are shown with maximum intensity projections. Color bars represent the partial R^2 of the maltreatment predictor. Empty color bars indicate that no voxels survived the corresponding significance threshold. Results are shown thresholded using the uncorrected thresholds $p_{unc}<.01$ (left column) and $p_{unc}<.001$ (right column).

Figure S50

Significant clusters across cohort-wise analyses (replicability analysis) for ***model 9*** *– CTQ subscale sexual abuse as a predictor in HC and MDD samples controlling for MDD diagnosis –* ***sex-stratified for male subsample***

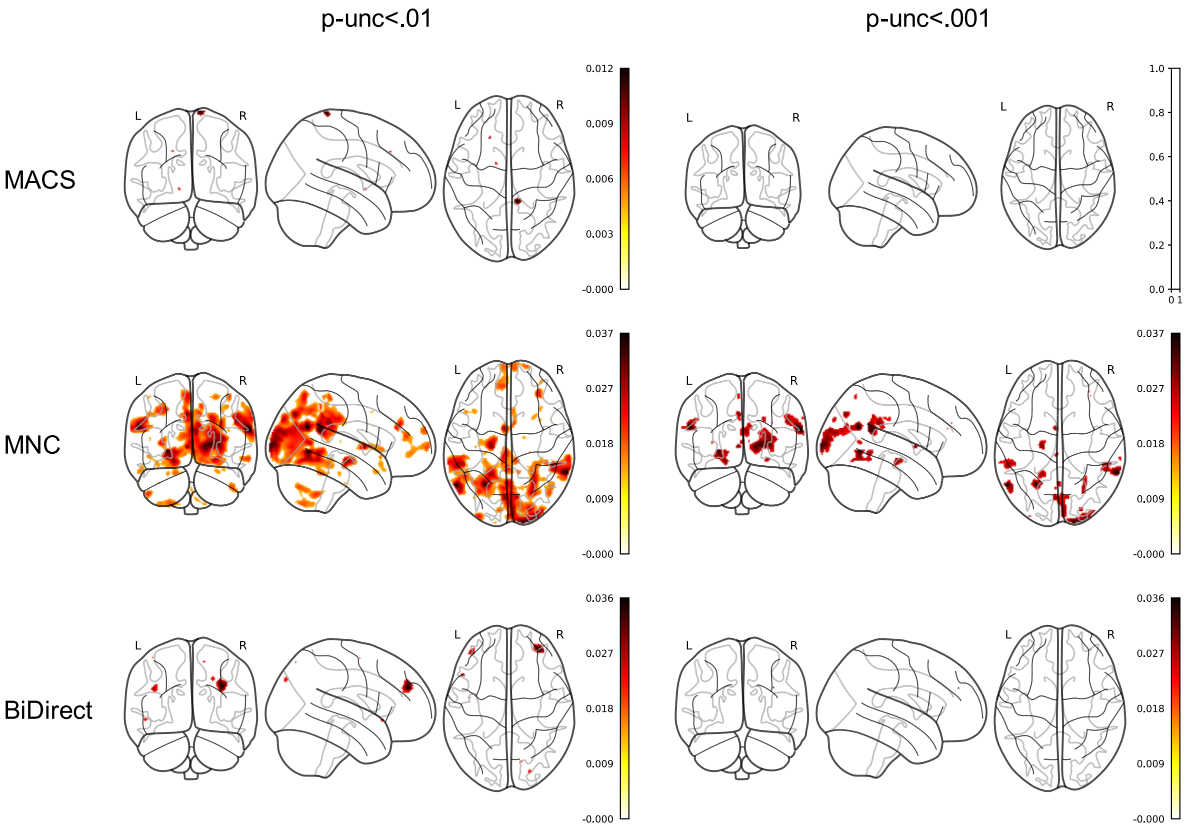

Note. Glass brains are shown with maximum intensity projections. Color bars represent the partial R^2 of the maltreatment predictor. Empty color bars indicate that no voxels survived the corresponding significance threshold. Results are shown thresholded using the uncorrected thresholds p_{unc}<.01 (left column) and p_{unc}<.001 (right column).

Figure S51

*Significant clusters across cohort-wise analyses (replicability analysis) for **model 10** – CTQ subscale emotional neglect as a predictor in HC and MDD samples controlling for MDD diagnosis – **sex-stratified for male subsample***

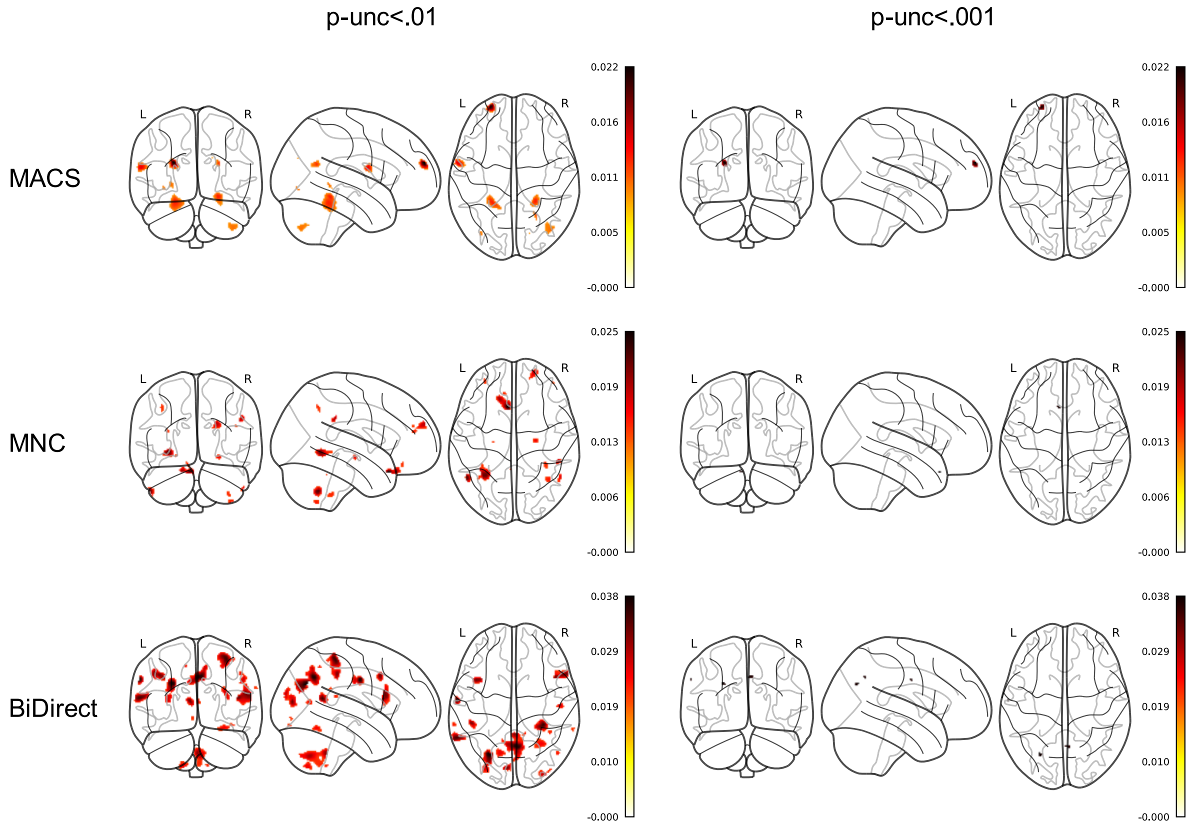

Note. Glass brains are shown with maximum intensity projections. Color bars represent the partial R^2 of the maltreatment predictor. Empty color bars indicate that no voxels survived the corresponding significance threshold. Results are shown thresholded using the uncorrected thresholds p_{unc}<.01 (left column) and p_{unc}<.001 (right column).

Figure S52

Significant clusters across cohort-wise analyses (replicability analysis) for ***model 11*** *– CTQ subscale physical neglect as a predictor in HC and MDD samples controlling for MDD diagnosis –* ***sex-stratified for male subsample***

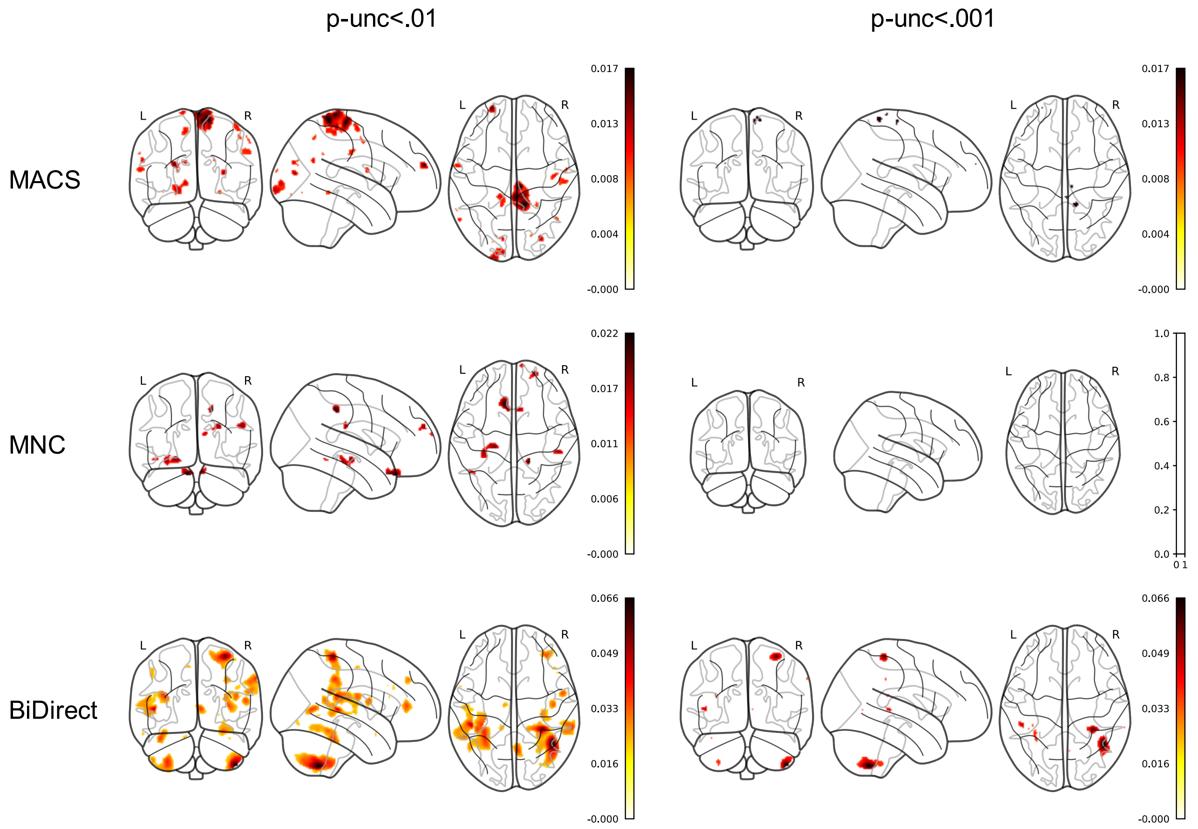

Note. Glass brains are shown with maximum intensity projections. Color bars represent the partial R^2 of the maltreatment predictor. Empty color bars indicate that no voxels survived the corresponding significance threshold. Results are shown thresholded using the uncorrected thresholds p_{unc}<.01 (left column) and p_{unc}<.001 (right column).

Figure S53

*Significant clusters across cohort-wise analyses (replicability analysis) for **model 12** – CTQ extreme severity compared to none in HC and MDD samples controlling for MDD diagnosis – **sex-stratified for male subsample***

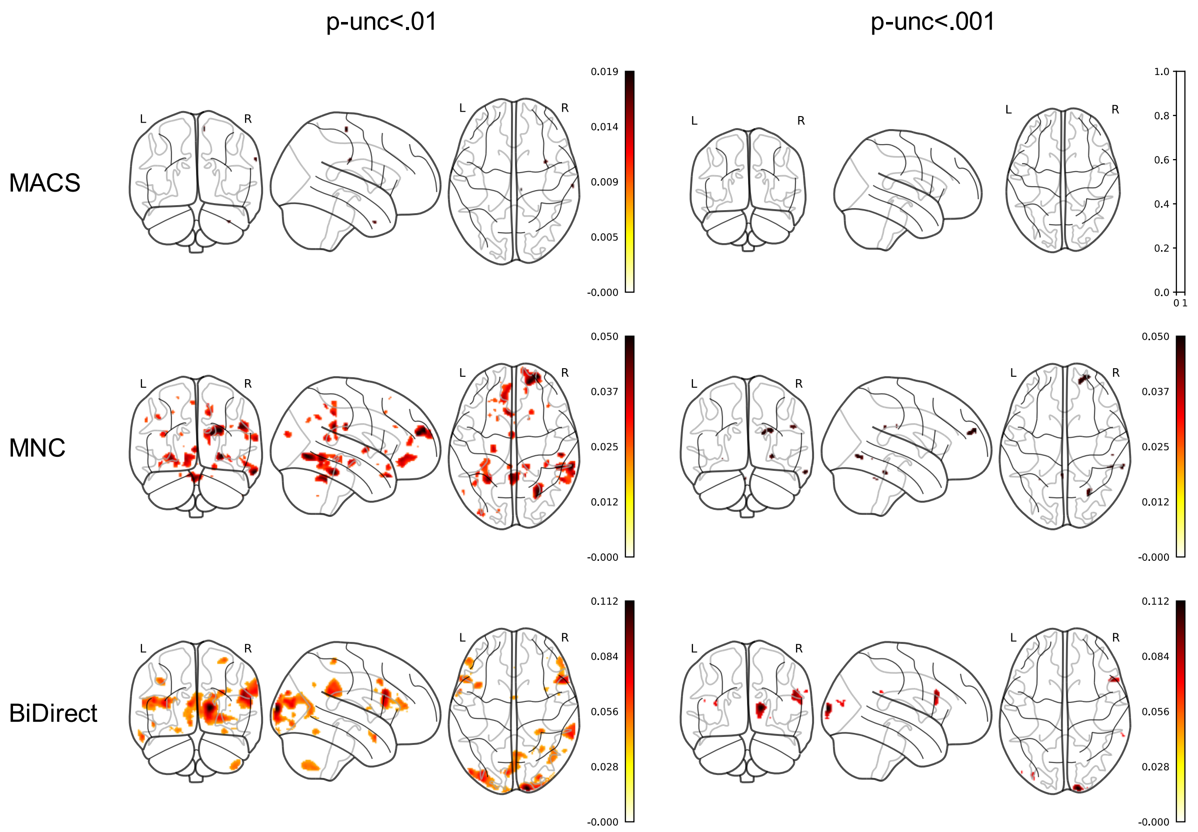

Note. Glass brains are shown with maximum intensity projections. Color bars represent the partial R^2 of the maltreatment predictor. Empty color bars indicate that no voxels survived the corresponding significance threshold. Results are shown thresholded using the uncorrected thresholds p_{unc}<.01 (left column) and p_{unc}<.001 (right column).

Figure S54

*Significant clusters across cohort-wise analyses (replicability analysis) for **model 13** – CTQ extreme severity compared to none in HC and MDD samples without controlling for MDD diagnosis – **sex-stratified for male subsample***

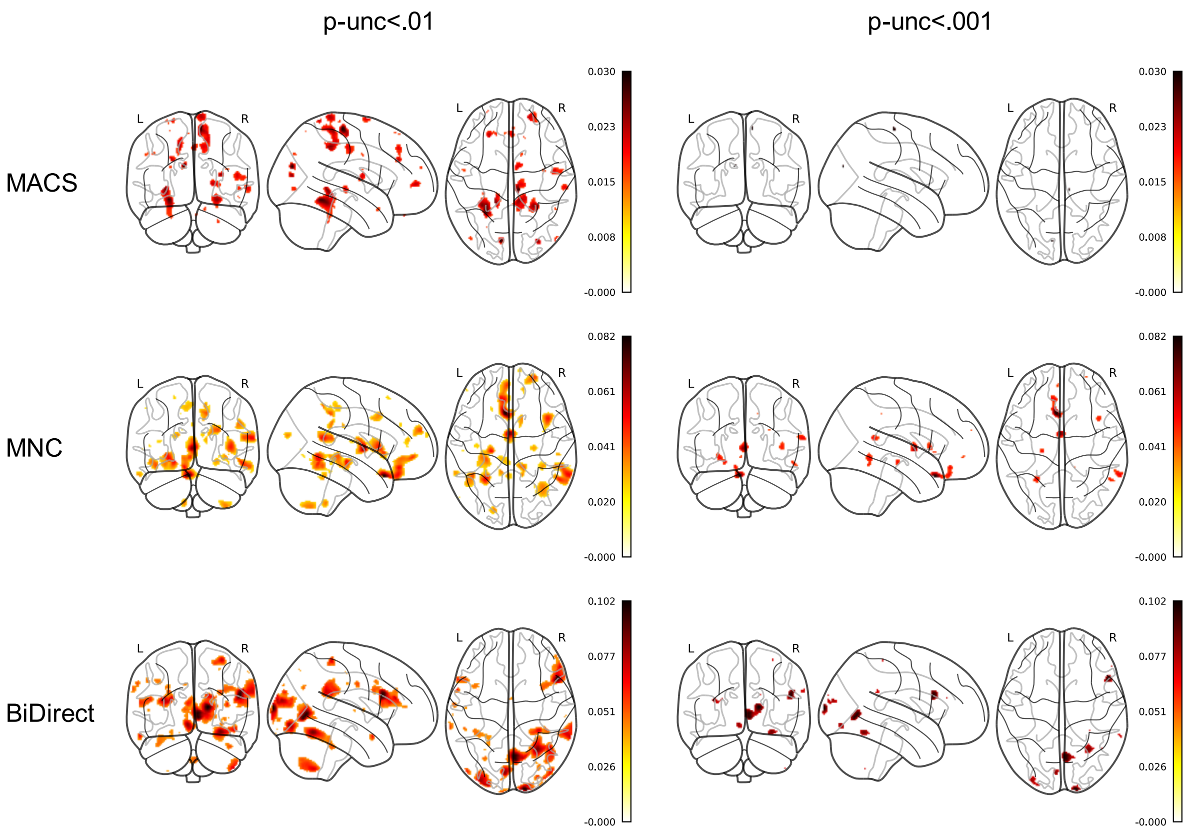

Note. Glass brains are shown with maximum intensity projections. Color bars represent the partial R^2 of the maltreatment predictor. Empty color bars indicate that no voxels survived the corresponding significance threshold. Results are shown thresholded using the uncorrected thresholds p_{unc}<.01 (left column) and p_{unc}<.001 (right column).

Figure S55

Significant clusters across cohort-wise analyses (replicability analysis) for ***model 14*** *– CTQ extreme severity compared to none in HC subsamples –* ***sex-stratified for male subsample***

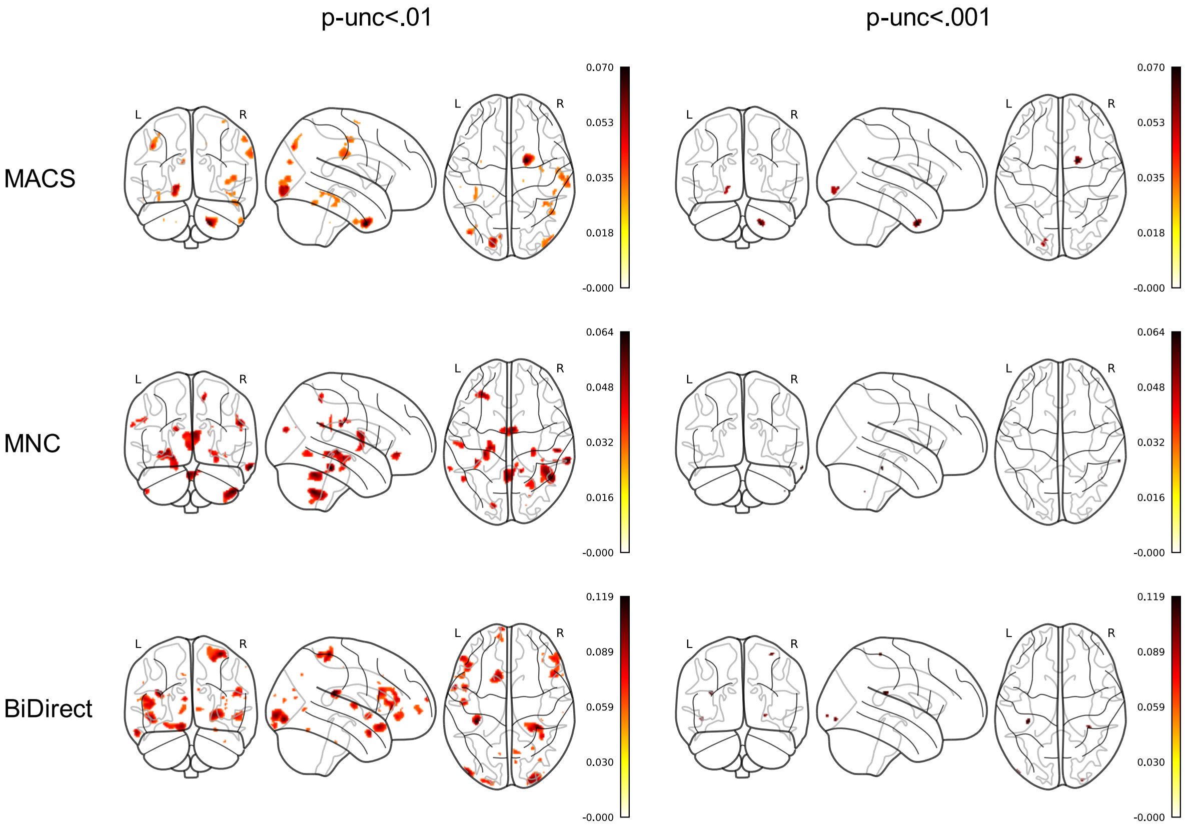

Note. Glass brains are shown with maximum intensity projections. Color bars represent the partial R^2 of the maltreatment predictor. Empty color bars indicate that no voxels survived the corresponding significance threshold. Results are shown thresholded using the uncorrected thresholds $p_{unc}<.01$ (left column) and $p_{unc}<.001$ (right column).

Figure S56

*Significant clusters across cohort-wise analyses (replicability analysis) for **model 15** – CTQ extreme severity compared to none in MDD subsamples – **sex-stratified for male subsample***

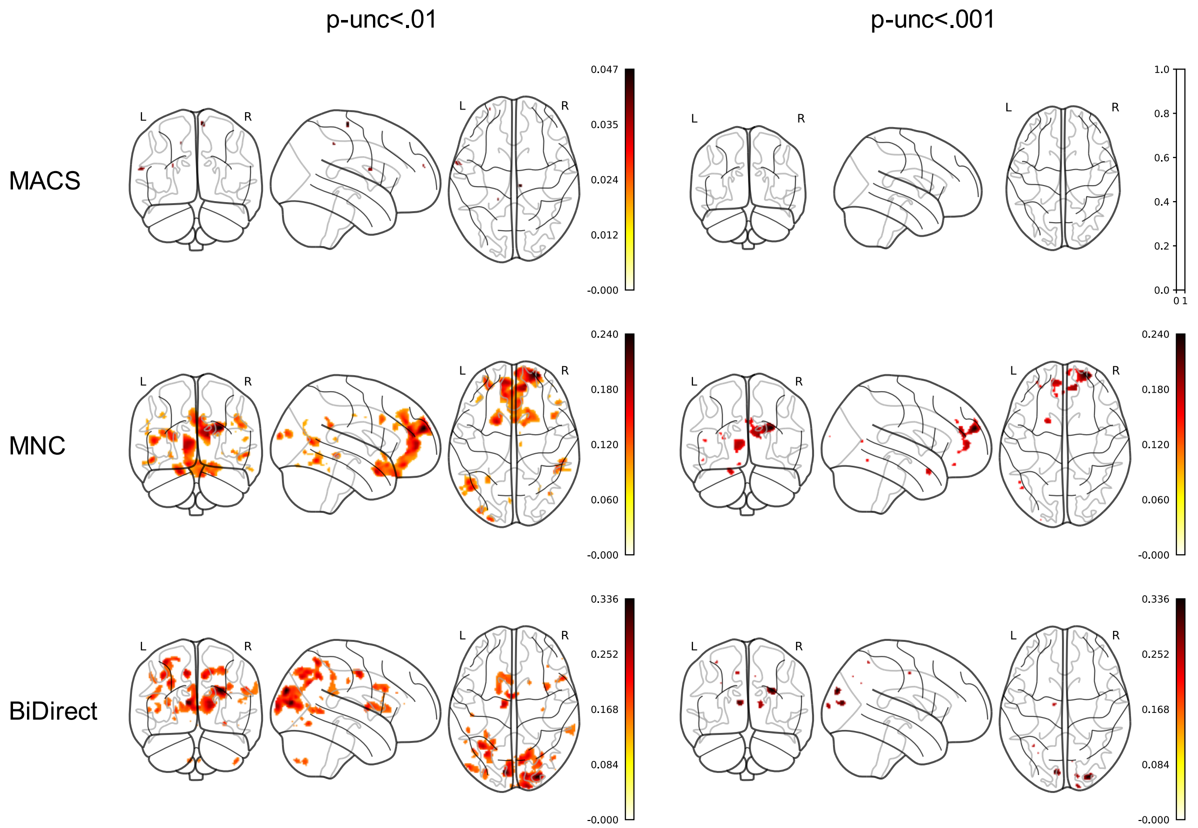

Note. Glass brains are shown with maximum intensity projections. Color bars represent the partial R^2 of the maltreatment predictor. Empty color bars indicate that no voxels survived the corresponding significance threshold. Results are shown thresholded using the uncorrected thresholds p_{unc}<.01 (left column) and p_{unc}<.001 (right column).

Figure S57

Significant clusters across cohort-wise analyses (replicability analysis) for ***model 16*** *– CTQ sum as a predictor in medication-naïve MDD subsamples –* ***sex-stratified for male subsample***

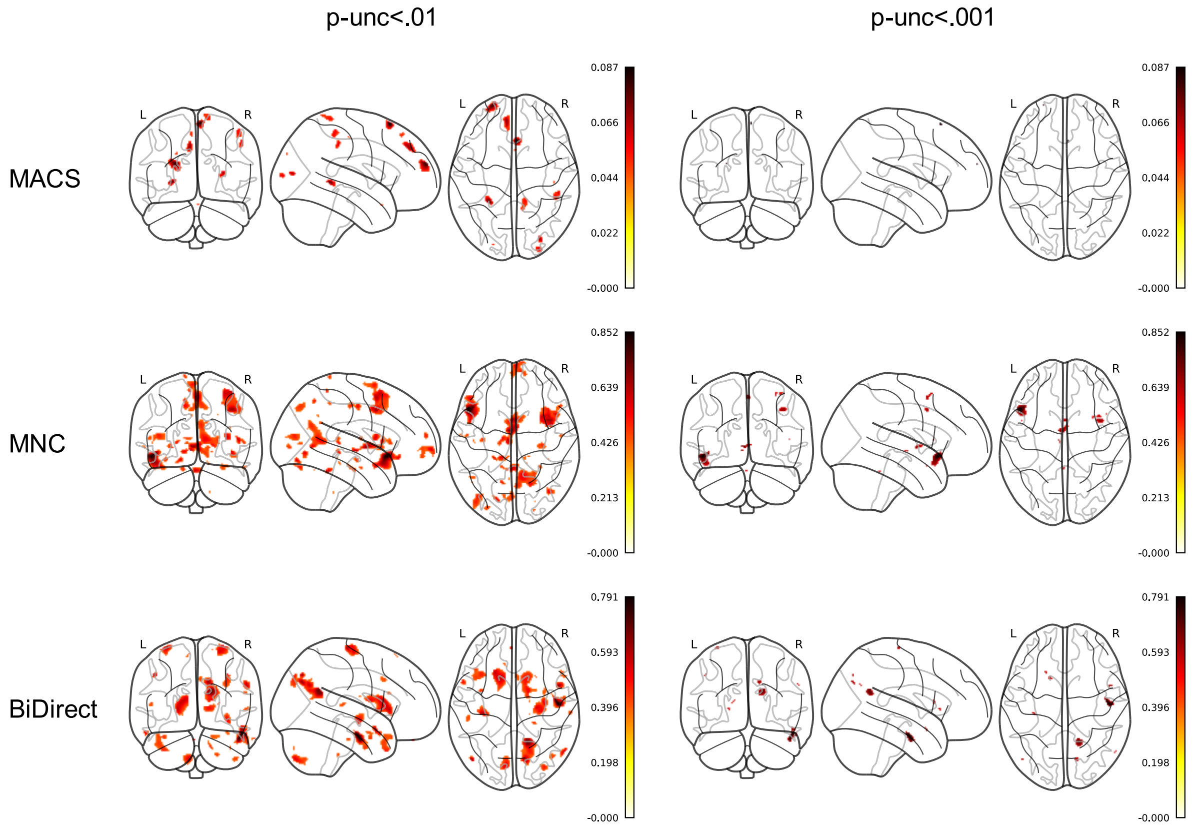

Note. Glass brains are shown with maximum intensity projections. Color bars represent the partial R^2 of the maltreatment predictor. Empty color bars indicate that no voxels survived the corresponding significance threshold. Results are shown thresholded using the uncorrected thresholds $p_{unc}<.01$ (left column) and $p_{unc}<.001$ (right column).

Figure S58: *Significant clusters across cohort-wise analyses (replicability analysis) for* ***model 17*** *– interaction term between CTQ sum and age as a predictor in HC subsamples –* ***sex-stratified for male subsample***

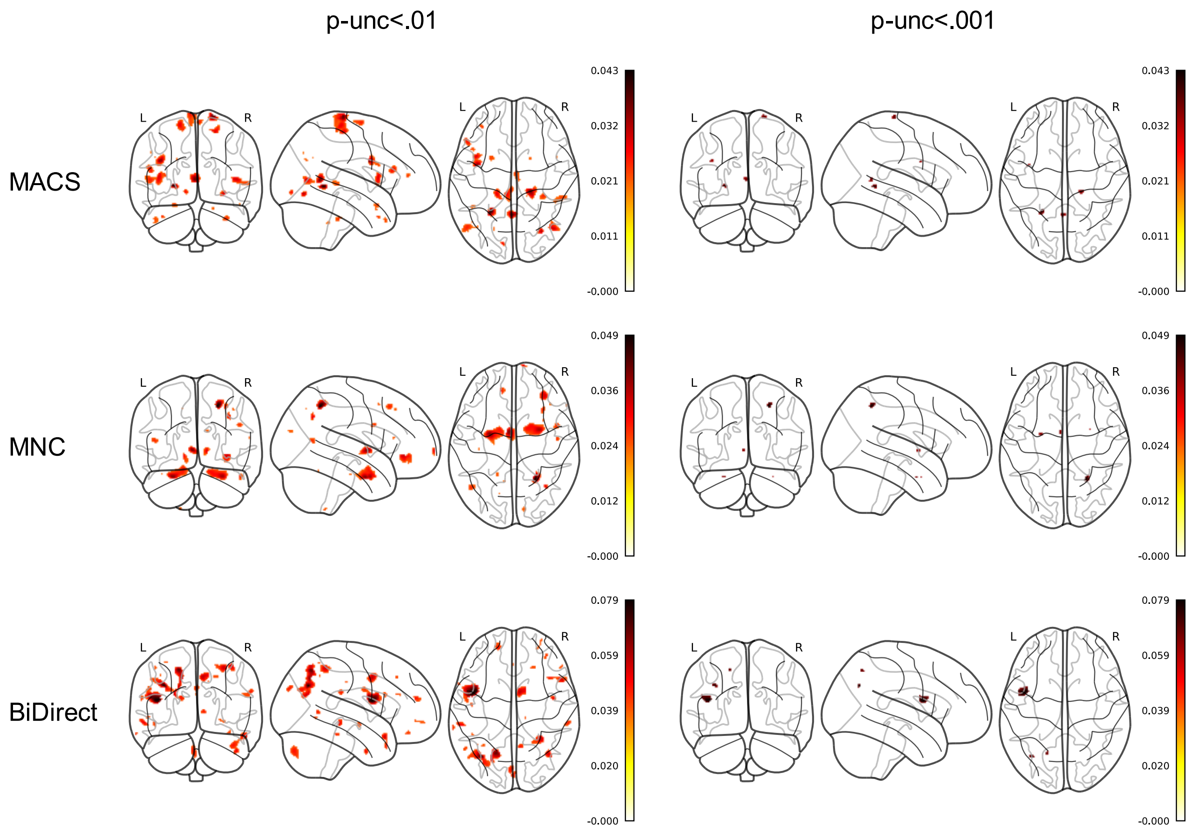

Note. Glass brains are shown with maximum intensity projections. Color bars represent the partial R^2 of the maltreatment predictor. Empty color bars indicate that no voxels survived the corresponding significance threshold. Results are shown thresholded using the uncorrected thresholds p_{unc}<.01 (left column) and p_{unc}<.001 (right column).

Figure S59

Significant clusters across cohort-wise analyses (replicability analysis) for ***model 18*** *– interaction term between CTQ sum and age as a predictor in MDD subsamples –* ***sex-stratified for male subsample***

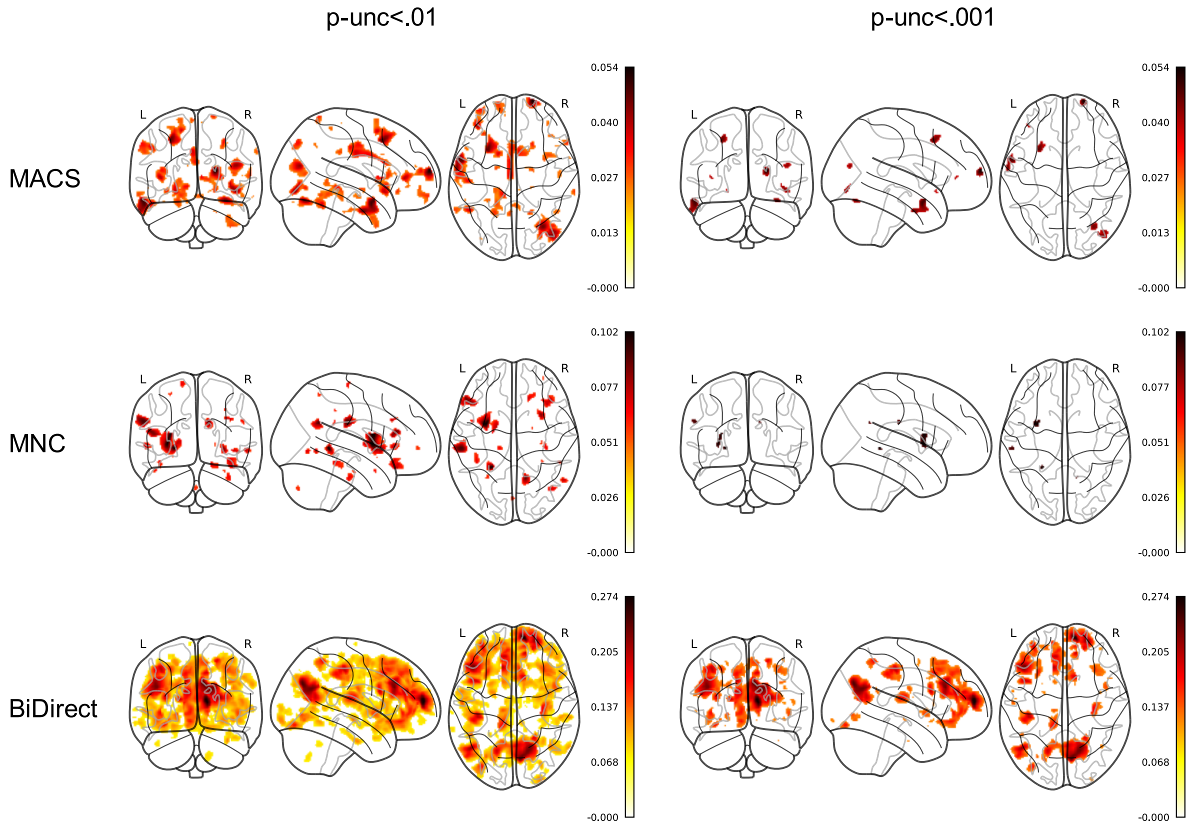

Note. Glass brains are shown with maximum intensity projections. Color bars represent the partial R^2 of the maltreatment predictor. Empty color bars indicate that no voxels survived the corresponding significance threshold. Results are shown thresholded using the uncorrected thresholds $p_{unc}<.01$ (left column) and $p_{unc}<.001$ (right column).

Figure S60

Permuted null distribution of significance overlap at uncorrected thresholds of p<.01 and p<.001, exemplary for Model 1 (not stratified by sex)

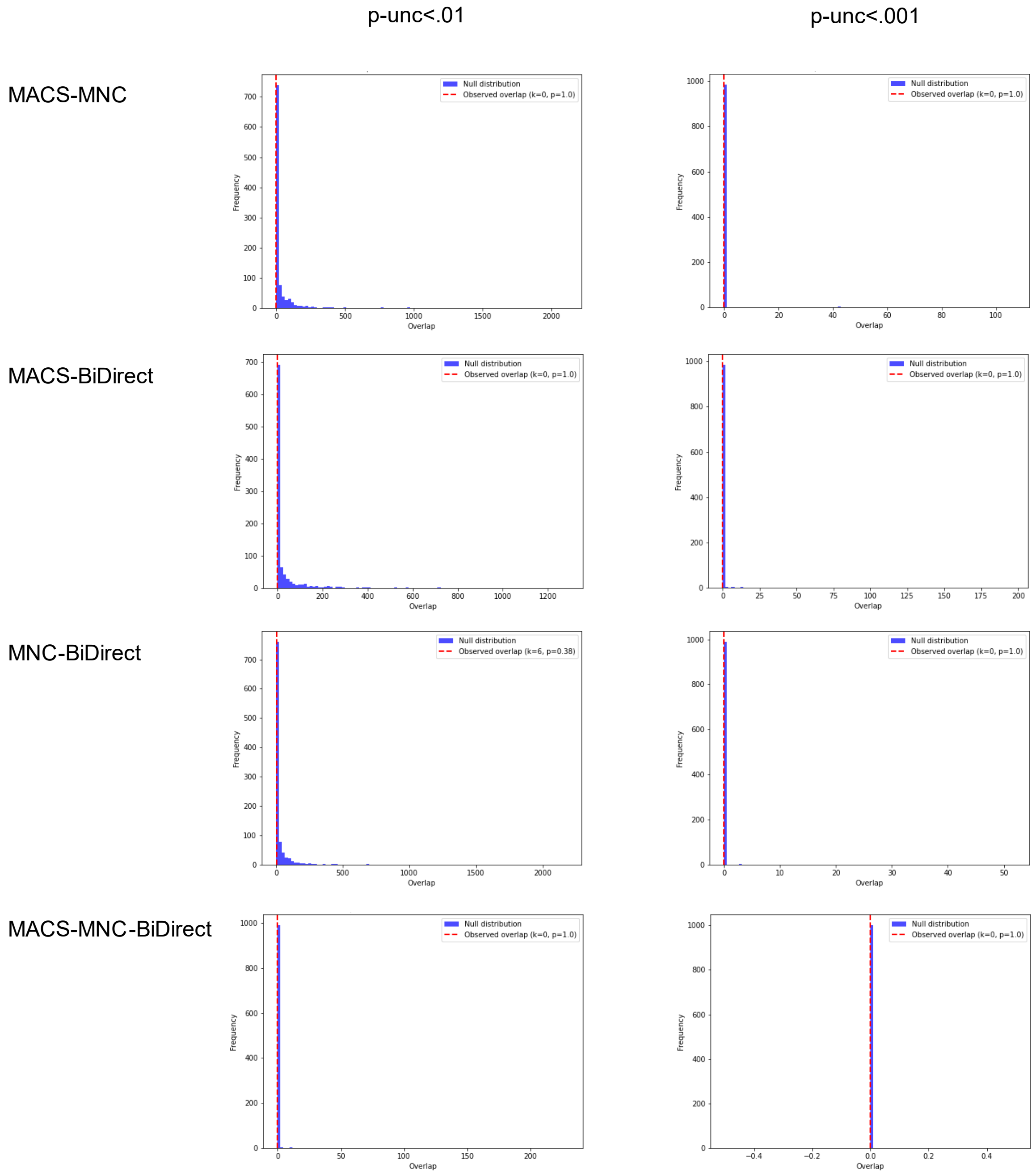

Supplement: Supplementary file 1 — Supplementary Information [file 41467_2025_62374_MOESM1_ESM.pdf]
