# Supplementary material for: Gray matter correlates of childhood maltreatment lack replicability in a multi-cohort brain-wide association study
Source: Nat Commun. 2025 Sep 16;16:8290. doi: 10.1038/s41467-025-62374-w (PMC12441139; doi:10.1038/s41467-025-62374-w)
Supplement: Supplementary file 3 — Reporting Summary [file 41467_2025_62374_MOESM3_ESM.pdf]

Corresponding author(s): Janik Goltermann

Last updated by author(s): Jul 9, 2025

Reporting Summary

Nature Portfolio wishes to improve the reproducibility of the work that we publish. This form provides structure for consistency and transparency in reporting. For further information on Nature Portfolio policies, see our Editorial Policies and the Editorial Policy Checklist.

Statistics

For all statistical analyses, confirm that the following items are present in the figure legend, table legend, main text, or Methods section.

n/a	Confirmed	
☐	☒	The exact sample size (*n*) for each experimental group/condition, given as a discrete number and unit of measurement
☐	☒	A statement on whether measurements were taken from distinct samples or whether the same sample was measured repeatedly
☐	☒	The statistical test(s) used AND whether they are one- or two-sided<br>*Only common tests should be described solely by name; describe more complex techniques in the Methods section.*
☐	☒	A description of all covariates tested
☐	☒	A description of any assumptions or corrections, such as tests of normality and adjustment for multiple comparisons
☐	☒	A full description of the statistical parameters including central tendency (e.g. means) or other basic estimates (e.g. regression coefficient) AND variation (e.g. standard deviation) or associated estimates of uncertainty (e.g. confidence intervals)
☐	☒	For null hypothesis testing, the test statistic (e.g. *F*, *t*, *r*) with confidence intervals, effect sizes, degrees of freedom and *P* value noted<br>*Give P values as exact values whenever suitable.*
☒	☐	For Bayesian analysis, information on the choice of priors and Markov chain Monte Carlo settings
☒	☐	For hierarchical and complex designs, identification of the appropriate level for tests and full reporting of outcomes
☐	☒	Estimates of effect sizes (e.g. Cohen's *d*, Pearson's *r*), indicating how they were calculated

Our web collection on statistics for biologists contains articles on many of the points above.

Software and code

Policy information about availability of computer code

Data collection	NA
Data analysis	Analysis was conducted using python version 3.9.12. Most central packages utilized were the following: nilearn version 0.9.1, neuroCombat version 0.2.12, atlasreader version 0.1.2. A comprehensive list of utilized packages is given in the analysis script which is made publicly available at https://osf.io/j8d9r/?view_only=9edf436ab18f4e8db9ef4c71c4ac356c

For manuscripts utilizing custom algorithms or software that are central to the research but not yet described in published literature, software must be made available to editors and reviewers. We strongly encourage code deposition in a community repository (e.g. GitHub). See the Nature Portfolio guidelines for submitting code & software for further information.

Data

Policy information about availability of data

All manuscripts must include a data availability statement. This statement should provide the following information, where applicable:

- Accession codes, unique identifiers, or web links for publicly available datasets
- A description of any restrictions on data availability
- For clinical datasets or third party data, please ensure that the statement adheres to our policy

Source data for all Tables and Figures are provided as a Source Data file. For all figures containing brain plots, source data are provided as NIfTI files. These files contain the statistical values and thresholds underlying each brain visualization. Comprehensive non-thresholded statistical estimates for all analyses are made

openly available via the OSF (https://osf.io/j8d9r/?view_only=9edf436ab18f4e8db9ef4c71c4ac356c). Individual raw data is not published due to current EU data protection regulations and the sensitive nature of clinical MRI data butdata but can be made available in form of summary statistics or anonymized aggregation of voxel-wise data upon reasonable request to the corresponding author, within four weeks, depending on the required data or results derivates.

Research involving human participants, their data, or biological material

Policy information about studies with human participants or human data. See also policy information about sex, gender (identity/presentation), and sexual orientation and race, ethnicity and racism.

Reporting on sex and gender	With 'sex' we refer to the biological sex of participants (self-reported). For the MACS sample this information was additionally verified using genetic data.
Reporting on race, ethnicity, or other socially relevant groupings	The utilized datasets define Western-European ethnicity as a general inclusion criterion as these ongoing studies were originally built to investigate genetic effects in the context of mental health. The inclusion of Western-European samples is fully disclosed in our manuscript (supplementary information) and related limitations regarding the generalizability of our findings are discussed within the discussion section of our manuscript.
Population characteristics	See manuscript and supplementary information file
Recruitment	Recruitment strategies were manifold, including recruiting inpatients at local psychiatric hospitals and advertising at local ambulatory health care institutions and newspapers. A selection bias cannot be ruled out. However, the utilization of a variety of recruitment strategies is likely to minimize potential selection bias (e.g., close cooperation with psychiatric hospitals enabled recruitment of severe cases of depression in inpatients).
Ethics oversight	The utilized studies were approved by the ethics committees of the Medical Faculty at the University of Münster (MACS: AZ 2014-422-b-S; MNC: AZ 2007-307-f-S; BiDirect: AZ 2009-391-f-S) and by the Medical Faculty of the University of Marburg (MACS: 07/14).

Note that full information on the approval of the study protocol must also be provided in the manuscript.

Field-specific reporting

Please select the one below that is the best fit for your research. If you are not sure, read the appropriate sections before making your selection.

☒ Life sciences ☐ Behavioural & social sciences ☐ Ecological, evolutionary & environmental sciences

For a reference copy of the document with all sections, see nature.com/documents/nr-reporting-summary-flat.pdf

Life sciences study design

All studies must disclose on these points even when the disclosure is negative.

Sample size	A convenience sample size was used (maximum available sample from existing datasets) in order to maximize the statistical power. The achieved sample size represents the largest voxel-wise investigation of neural correlates of childhood maltreatment that is not a meta-analysis. Based on the works by Marek and Tervo-Clemmens et al. (2022, Nature), Liu et al. (2023, Nature Human Behaviour) our large sample should be sufficiently powered to detect even small effects (e.g., corresponding to r=.1), which are common in mental health neuroimaging.
Data exclusions	Data exclusions are comprehensively described within the supplementary information. These were not pre-established prior to data collection.
Replication	The assessment of the replicability of our findings was at the heart of this research project and is comprehensively described within the manuscript.
Randomization	NA (covariates explained within the manuscript)
Blinding	No randomization was conducted and blinding was not possible. Due to highly standardized measurement of variables, this is not assumed to be problematic (particularly, MRI assessments are not subject to researcher bias).

Reporting for specific materials, systems and methods

We require information from authors about some types of materials, experimental systems and methods used in many studies. Here, indicate whether each material, system or method listed is relevant to your study. If you are not sure if a list item applies to your research, read the appropriate section before selecting a response.

Materials & experimental systems

n/a	Involved in the study
☒	☐ Antibodies
☒	☐ Eukaryotic cell lines
☒	☐ Palaeontology and archaeology
☒	☐ Animals and other organisms
☒	☐ Clinical data
☒	☐ Dual use research of concern
☒	☐ Plants

Methods

n/a	Involved in the study
☒	☐ ChIP-seq
☒	☐ Flow cytometry
☐	☒ MRI-based neuroimaging

Plants

Seed stocks: NA

Novel plant genotypes: NA

Authentication: NA

Magnetic resonance imaging

Experimental design

Design type: Structural MRI (no design, no task)

Design specifications: NA

Behavioral performance measures: NA

Acquisition

Imaging type(s): T1-weighted structural imaging of gray matter structure

Field strength: 3T

Sequence & imaging parameters: MACS: Fast gradient echo sequences (MPRAGE) including the following parameters. Münster: Prisma (Siemens, Erlangen, Germany), 192 sagittal slices, TR=2130ms, TE=2.28ms, inversion time=900ms, FA=8°, resulting in a voxel size of 1x1x1mm³. Marburg: Tim Trio (Siemens, Erlangen, Germany), 176 sagittal slices, TR=1900ms, TE=2.26ms, inversion time=900ms, FA=9°, resulting in a voxel size of 1x1x1mm³.

MNC: Gyroscan Intera 3 T (Philips Medical Systems, Best, NL) with fast gradient echo sequence (turbo field echo), with a repetition time of 7.4 ms, echo time = 3.4 ms, flip angle = 9°, two signal averages, inversion prepulse every 814.5 ms, acquired over a field of view of 256 (feet-head) x 204 (anterior-posterior) x 160 (right-left) mm

BiDirect: turbo field echo imaging was acquired with 160 sagittal slices with a thickness of 2 mm (reconstructed to 1 mm), resulting in a voxel size of 1 × 1 × 1 mm (TR = 7.26 ms, TE = 3.56 ms, 9° flip angle, matrix dimension 256 × 256, FOV = 256 × 256 mm).

Area of acquisition: All analyses were conducted on a whole brain level

Diffusion MRI: ☐ Used ☒ Not used

Preprocessing

Preprocessing software: Image preprocessing was conducted using the CAT12-toolbox (Gaser et al., 2022; https://neuro-jena.github.io/cat/, version r1720) using default parameters equally for all cohorts

Normalization: linear (12-parameter affine) and non-linear transformations, within a unified model including high-dimensional geodesic shooting normalization

Normalization template | Images were normalized in MNI space

Noise and artifact removal | Standardized quality assurance protocols were applied including the check homogeneity function of CAT12 and visual inspection of statistical outliers

Volume censoring | NA

Statistical modeling & inference

Model type and settings | Standard mass-univariate general linear models were used for the cross-sectional data (no within-subject factors), including group-based predictors and continuous predictors

Effect(s) tested | A series of statistical models was conducted. These are comprehensively described within the manuscript

Specify type of analysis: [x] Whole brain [ ] ROI-based [ ] Both

Statistic type for inference (See Eklund et al. 2016) | Statistical inference was done based on different statistical thresholds: Voxel-wise FWE-corrected p<.05, uncorrected p<.001 and uncorrected p<.01. P-values were calculated based on brain-wide t-maps.

Correction | Voxel-level FWE correction based on max-T method, as implemented in the nilearn python package (permuted_ols function)

Models & analysis

n/a	Involved in the study
[x]	[ ] Functional and/or effective connectivity
[x]	[ ] Graph analysis
[x]	[ ] Multivariate modeling or predictive analysis
